# Beyond the vaccines: a glance at the small molecule and peptide-based anti-COVID19 arsenal

**DOI:** 10.1186/s12929-022-00847-6

**Published:** 2022-09-06

**Authors:** Kunal Nepali, Ram Sharma, Sachin Sharma, Amandeep Thakur, Jing-Ping Liou

**Affiliations:** 1 School of Pharmacy, College of Pharmacy, Taipei Medical University, 250 Wuxing Street, Taipei, 11031 Taiwan; 2 TMU Research Center for Drug Discovery, Taipei Medical University, Taipei, 11031 Taiwan

**Keywords:** Coronaviruses, COVID-19, SARS-CoV-2, ACE-2, TMPRSS2 drug repurposing, Medicinal chemistry, Viral replication, Viral entry, RdRp inhibitors, 3CLpro inhibitors, PLpro inhibitors, TMPRSS2 inhibitors, Peptides, Small molecule inhibitors, Vaccines, Monoclonal antibodies

## Abstract

Unprecedented efforts of the researchers have been witnessed in the recent past towards the development of vaccine platforms for the control of the COVID-19 pandemic. Albeit, vaccination stands as a practical strategy to prevent SARS-CoV-2 infection, supplementing the anti-COVID19 arsenal with therapeutic options such as small molecules/peptides and antibodies is being conceived as a prudent strategy to tackle the emerging SARS-CoV-2 variants. Noteworthy to mention that collective efforts from numerous teams have led to the generation of a voluminous library composed of chemically and mechanistically diverse small molecules as anti-COVID19 scaffolds. This review article presents an overview of medicinal chemistry campaigns and drug repurposing programs that culminated in the identification of a plethora of small molecule-based anti-COVID19 drugs mediating their antiviral effects through inhibition of proteases, S protein, RdRp, ACE2, TMPRSS2, cathepsin and other targets. In light of the evidence ascertaining the potential of small molecule drugs to approach conserved proteins required for the viral replication of all coronaviruses, accelerated FDA approvals are anticipated for small molecules for the treatment of COVID19 shortly. Though the recent attempts invested in this direction in pursuit of enrichment of the anti-COVID-19 armoury (chemical tools) are praiseworthy, some strategies need to be implemented to extract conclusive benefits of the recently reported small molecule viz. (i) detailed preclinical investigation of the generated anti-COVID19 scaffolds (ii) in-vitro profiling of the inhibitors against the emerging SARS-CoV-2 variants (iii) development of assays enabling rapid screening of the libraries of anti-COVID19 scaffold (iv) leveraging the applications of machine learning based predictive models to expedite the anti-COVID19 drug discovery campaign (v) design of antibody–drug conjugates.

**Supplementary Information:**

The online version contains supplementary material available at 10.1186/s12929-022-00847-6.

## Background

The world is currently in the throes of the pandemic coronavirus infection, called Coronavirus disease-2019 (COVID-2019). COVID-2019 is a baffling respiratory infectious disease evoked by severe acute respiratory syndrome coronavirus 2 (SARS-CoV-2) [1–3]. The genetic sequencing of the virus identifies it to be a novel beta-coronavirus, an enveloped, positive single-strand RNA virus, named 2019 novel coronavirus (2019-nCoV). Closely linked to the SARS virus, SARS-CoV-2 bears characteristic “crown-like” spikes on its surface and belongs to the Orthocoronavirinae subfamily. It is believed that a person-to-person transmission may occur through droplet or contact [4–11]. The infection starts with the entry of the virus, through identification of receptor, priming of surface protein followed by endocytosis, and fusion of membrane. Specifically, the interaction of the receptor binding domain (RBD) of the viral spike glycoprotein with ACE2 leads to the entry of the virus into the host cells (viral entry stage). This is followed by the initiation of RNA replication (viral replication stage) and then the release of new virions to infect other cells in the host [12, 228].

**Fig. 1:**
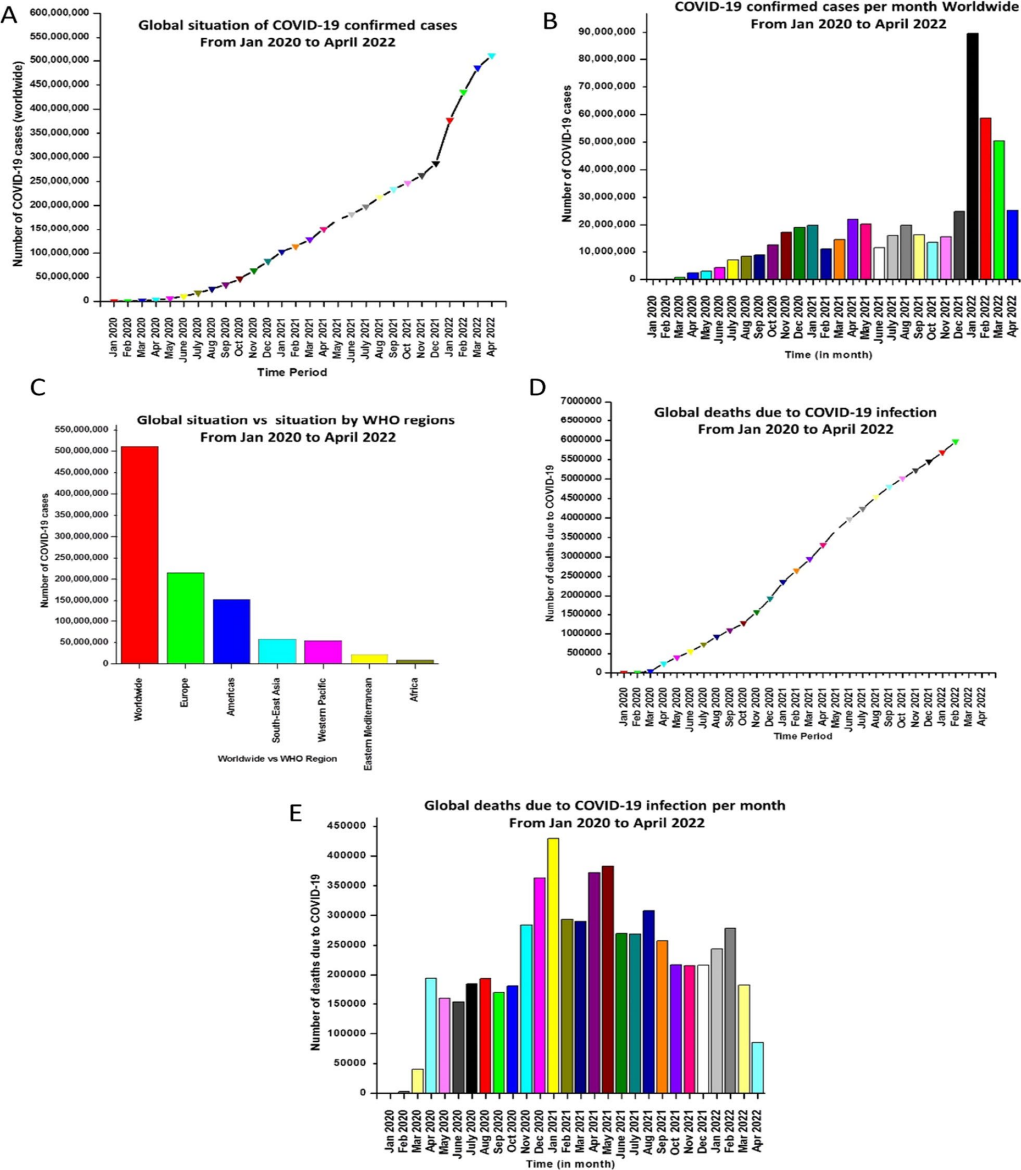
**A** Cumulative COVID19 confirmed cases, worldwide **B** COVID19 Confirmed cases per month (from Jan 2020 to April 2022) **C** COVID19 cases distribution Worldwide vs WHO Regions **D** Global cumulative deaths due to COVID19 infection **E** Deaths due to COVID19 infection per month (data collected from Jan 2020 to April 2022)

The current global scenario marred by huge numbers of infected people (Fig. 1) [249] called for an urgent demand to develop affordable vaccines to render active acquired immunity to the vulnerable population. Subsequently, many laboratories initiated the important pursuit of fabricating vaccines against COVID19. Resultantly, the efforts invested culminated in the furnishment of different vaccine platforms (mRNA-based vaccines, recombinant protein-based vaccines, inactivated virus and protein subunit-based vaccines and viral vector-based vaccines). Gratifyingly, timely actions from the biotech industry led to the approval of several vaccines. Owing to the documented progress (initial /interim reports and final disclosures) in the context of vaccine efficacy, it appeared that vaccination is the most prudent approach to end the pandemic. However, reduced susceptibility to vaccine-induced immunity owing to the emergence of multiple SARS-CoV-2 variants, particularly the rise of omicron, has emerged as a major threat in the context of the clinical utility of COVID-19 vaccines [13]. Moreover, a recently published systematic review and meta-regression study published in Lancet revealed that the efficacy or the effectiveness of the vaccine against infection and symptomatic disease decreased by approximately 20–30 percentage points within 6 months. Waning immunity has been assumed to be partly responsible for the decreased efficacy. Thus, the factors responsible for the rapid waning in vaccine immunity are required to be clearly understood to develop efficacious vaccines [14, 229, 230]. Collectively, factors such as SARS-CoV-2 variants, vaccine inequity and the need for booster doses to restore the vaccine effectiveness make it clear that the battle against this notorious virus is far from over [15]. In this context, efforts are required to be directed towards the development of new vaccines that can elicit durable protection and are capable of tackling the SARS CoV-2 variants. In addition, it is also envisioned that looking beyond the vaccines and supplementing the anti-COVID19 arsenal with other therapeutic options might turn out to be a prudent strategy. Noteworthy to mention that there is an ongoing wave in the field of anti-COVID19 therapeutic modality development towards monoclonal antibodies, peptide-based drugs and small molecule inhibitors. For antibody-based anti-COVID19 therapeutic development, antibodies derived from SARS patients that target RBDs on spike surface proteins and neutralize various SARS-CoVs with similar RBDs have been explored [16]. Explorations were also directed towards antibodies that target the external part of the surface subunit of SARS-CoV-2 (S1A/NTD) in the quest to prepare broad-spectrum antibodies against SARS-CoVs [17, 18]. Resultantly, the armoury of monoclonal antibodies (mAbs) for COVID-19 is presently loaded with a handful of options such as sotrovimab (VIR-7831, an antibody drug), Lenzilumab (an engineered anti-human granulocyte macrophage colony-stimulating factor mAb), Bamlanivimab (a recombinant human IgG1 mAb antibody), Casirivimab & Imdevimab (REGEN-COV, neutralizing antibody), Tixagevimab & cilgavimib (AZD7442, antibody cocktail), BRII-196/BRII-198 (SARS-CoV-2 negativing mAb combination remedy), CERC002 (a fully human mAb against LIGHT or TNFSF14), SAB185 (polyclonal antibodies), Regdanvimab (recombinant human mAb), Sarilumab (IL-6) receptor antagonist) and Tocilizumab (anti-IL-6 receptor mAb) [19]. Gratifyingly, the results of the investigations on monoclonal antibodies targeting SARS-CoV-2 were optimistic that led to several U.S. FDA approvals (Tables 1, 2) for emergency use of this therapeutic modality for the treatment of COVID-19. Casirivimab—Imdevimab, Bamlanivimab—Etesevimab, Sotrovimab, Tocilizumab, Tixagevimab—Cilgavimib and Bebtelovimab represents the FDA-approved mAbs for use in COVID-19 [20]. Albeit, the documented progress in this field is encouraging, continued investigations are required to attain a clear understanding of the therapeutic characteristics of these tools to extract more conclusive benefits in the context of their therapeutic or prophylactic applications against SARS-CoV-2 [19].

**Table 1:** FDA approvals (small molecules) [20]

Drugs	FDA approval status	Mechanism	Indications
Remdesivir	2020/05/01 EUA 2020/10/22 (Formal medicine certificate)	– Developer—Gilead – Brand Name—Veklury – Type—nucleoside analogue prodrug (small molecule) – Mechanism—Inhibits the replication of the virus (RNA polymerase inhibitor) – Activity profile—Remdesivir (GS-5734) demonstrates inhibitory potential towards SARS-CoV-2 in vitro. The studies conducted in animal models revealed that the inhibitor is endowed with the ability to inhibit MERS-CoV, SARS-CoV-1, and SARS-CoV-2 replication in animal models [27]	Approved for hospitalized patients, Adults and children (> 12 yr) (EUA) hospital, Children (3.5–40 kg or < 12 yr and > 3.5 kg) [20]
Baricitinib	2020/11/19 EUA 2021/07/28 EUA (can be used alone)	– Developer—Eli Lilly – Brand Name—Olumiant – Type—Small molecule – Mechanism—oral selective Janus Kinase 1/2 inhibitor – Activity profile—Baricitinib demonstrates antiviral and anti-inflammatory properties. Also, Barcitinib suppresses the overstimulation of the immune system [28] – A study results revealed that baricitinib—remdesivir combination was more efficacious than remdesivir alone in terms of reduction in the recovery time. Also, the cocktail of drugs caused improvement in clinical status among COVID-19 patients receiving high-flow oxygen or noninvasive ventilation [29]	Hospitalized, severe cases, adult and child (> 2 yr) [20]
Nirmatrelvir and ritonavir (Oral)	12/22/2021 EUA	– Developer—Pfizer – Brand Name—Paxlovid (nirmatrelvir and ritonavir) – Type—small molecule inhibitors – Mechanism—Paxlovid is a SARS-CoV-2-3CL protease inhibitor composed of nirmatrelvir and ritonavir. It inhibits viral replication at a proteolysis stage occurring before viral RNA replication. Nirmatrelvir is packaged with a strong cytochrome P450 3A4 inhibitor (ritonavir) which is used to boost HIV protease inhibitors – Activity profile—In comparison to the placebo, Paxlovid (PF-07321332; ritonavir) reduced the risk of hospitalization risk or death by 89% in high-risk adults with COVID-19 [30, 31]	Severe high risk and mild to medium, adult and child (> 12 yr or 40 kg) [20]
Molnupiravir	12/23/2021 EUA	– Developer—Merck – Brand name—Lagevrio – Type- Small*-*molecule nucleoside antiviral prodrug – Mechanism—Molnupiravir acts by increasing the frequency of viral RNA mutations frequency and leads to impairment of the SARS-CoV-2 replication [32] – Activity profile—effective reduction of viral load and complete prevention of virus transmission through direct contact with untreated animals was evidenced with Molnupiravir [33]	Severe high risk, mild and medium, adult [20]

**Table 2:** FDA approvals (monoclonal antibodies) [20]

Casirivimab and Imdevimab	2020/11/21 EUA 2021/08/10 EUA (post exposure prophylaxis)	– Developer—Regeneron – Brand name—REGEN-COV – Type—Neutralizing antibody – Mechanism—binds to RBD of the spike protein of SARS-CoV-2 and blocks its attachment to human ACE2 receptors, thereby preventing the entry of virus in the human cells. [34] – Activity profile—A cocktail of mAbs (casirivimab and imdevimab), REGEN-COV reduces the viral load (SARS-CoV-2). The combination also decreases the number of medical visits of COVID-19 patients. [35]	Mild to medium, adult and child (> 12 yr) Severe and high risk of adult and child (> 12 yr or 40 kg) prevention [20]
Bamlanivimab and Etesevimab	2021/02/09 EUA 2021/06/25 (Suspension) EUA 2021/08/27 (resume) EUA 2021/09/16 EUA (Post-exposure prophylaxis)	– Developer—Eli Lilly – Type—Neutralizing antibody – Mechanism—binds to RBD of the spike protein of SARS-CoV-2 – Activity profile—the combination could lower the incidence of hospitalization and death (COVID-19 related) as compared to placebo was evidenced with the combination of bamlanivimab plus etesevimab. Treatment with the combination also led to a decline in the SARS-CoV-2 viral load [36]	Mild to medium, adult and child (> 12 yr) Severe and high risk of adult and child (> 12 yr or 40 kg), prophylaxis [20]
Sotrovimab	2021/05/26 EUA	– Developer—GSK/ Vir Biotechnology – Brand name—Xevudy – Type—Neutralizing antibody – Mechanism—the antibody binds to a highly conserved epitope on the spike (S) protein (SARS-CoV-2) [37] – Activity Profile—In a study, treatment with the antibody reduced the risk of a composite end point of all-cause hospitalization or death [38]	Medium to severe, Adult and child (> 12 yr) [20]
Tocilizumab	2021/06/24 EUA	– Developer—Genentech – Brand Name—Actemra – Type—IL-6 monoclonal antibody – Mechanism—competitive inhibition of IL-6 binding to its receptor (IL-6R). [39] – Activity—Treatment with IL-6 monoclonal antibody reduces the progression likelihood to the composite outcome of mechanical ventilation or death in COVID-19 hospitalized patients with pneumonia [39]. Tocilizumab attenuates the inflammatory response in the cytokine storm [40]	Hospitalized, severe cases, adult and child (> 2 yr) [20]
Tixagevimab and& Cilgavimib	12/08/2021 EUA	– Developer—AsterZeneca – Brand name—Evusheld – Type—Neutralizing antibody – Mechanism—the antibody binds to the spike protein (non-overlapping portions, SARS-CoV-2) – Activity profile—The combination of antibodies (Tixagevimab plus cilgavimab) administered IM lowered the incidence of symptomatic COVID-19 in comparison to placebo. Administration of combination (every six months, Tixagevimab and cilgavimab) to eligible patients is recommended in case SARS-CoV-2 is in circulation. The cocktail of neutralizing antibodies is efficacious against the delta variant of SARS-CoV-2 [41]	Adult and child (> 12 yr or 40 kg), Prophylaxis [20]
Bebtelovimab	02/11/2022 EUA	– Developer—Eli Lilly – Type—Neutralizing IgG1 monoclonal antibody – Mechanism—Binds to spike protein of SARS-CoV-2 – Activity profile—Studies have demonstrated the retention of neutralization efficacy against all variants of SARS-CoV-2 except Mu variant. Notably, bebtelovimab retains the efficacy against SARS-CoV-2 omicron variants [42, 43]	Severe high risk and mild to medium, adult and child (> 12 yr or 40 kg) [20]

**Fig. 2:**
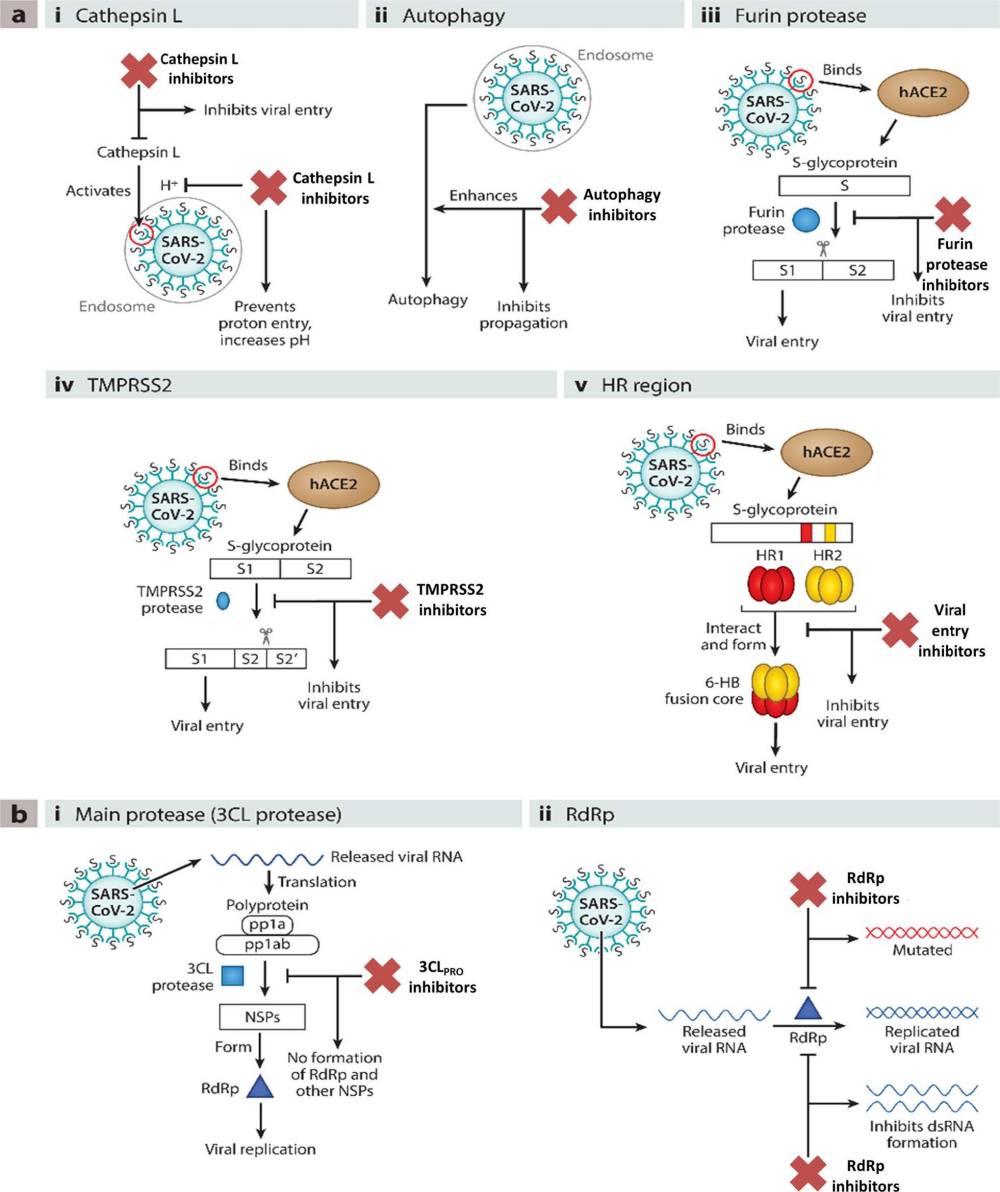
Key targets for the design of anti-COVID19 scaffolds. **a** i. Cathepsin L ii. Autophagy iii. Furin protease iv. TMPRSS2 v. HR region.** b** i. Main Protease (2CL protease) ii) RdR_P_ [11] (Reprinted with permission from Annu. Rev. Pharmacol. Toxicol. 2021; 61:465–93

Noteworthy to mention that the recent past has witnessed the utmost proficiency of the drug discovery enthusiasts in leveraging the list of existing therapeutic targets for the construction of pragmatically designed tractable anti-COVID 19 scaffolds (Additional file 1 and Fig. 2). Aligned with the usual trends in small molecule drug discovery endeavours, the task of developing effective therapeutics for COVID19 required a clear-cut understanding of the nature of the virus, sequence features (SARS-CoV-2), structural insights into virus–receptor interactions and the pathogenesis of the disease [11]. Pleasingly, exhaustive explorations were conducted to unravel the coronavirus structure [21, 231–234] and the insights gained through the aforementioned studies spurred the medicinal chemist to conduct numerous drug discovery endeavours to target viral entry as well as viral replication stage in pursuit of developing antiviral drug candidates (Fig. 2). Resultantly, several strategies were employed to steer the wheels of anti-COVID19 drug discovery campaigns. Specifically, 3C-like protease (3CLpro), papain-like protease (PLpro) and non-structural proteins (NSPs) encoded by SARS-CoV-2 that plays an imperative role in virus replication were exhaustively investigated as prudent targets for the construction of libraries of anti-COVID19 drugs [22]. Delightfully, the substantial efforts invested in this direction have paid dividends and enriched the chemical toolbox with a plethora of 3CLpro and PLpro inhibitors endowed with striking antiviral efficacy against COVID19. Notably, the research groups exercised a blend of classical drug design strategies as well as innovative approaches to constructing 3CLpro and PLpro inhibitors. In numerous instances, the medicinal chemist leveraged the crystallographic information for SARS CoV-1 3CL_PRO_ and PLpro for the design of new scaffolds (anti-SARS CoV-2 3CLpro inhibitors). Notably, in the quest to expand the size of the structure pool, pragmatically designed structural optimization programs (3CLpro and PLpro inhibitors) were conducted to fabricate covalently bound reversible/irreversible inhibitors, peptidomimetic/nonpeptidomimetic type frameworks and peptide-drug conjugates (PDCs) to deliver drug payloads. Importantly, high throughput drug screening program, as well as machine-based learning, proved to be a boon to the medicinal chemist in expediting some of the aforementioned drug discovery endeavours. RNA-dependent RNA polymerase (RdRp) and helicase as targets for COVID19 drugs have also been the subject of explorations [23, 235, 236]. It has been well established that the viral spike glycoprotein S protein binding to host cell angiotensin-converting enzyme 2 (ACE2) [24, 25] is responsible for SARS-CoV-2 entry into host cells. Also, S protein binding to the cellular transmembrane serine protease (TMPRSS2) promotes the virus entry into the host cells. In light of these disclosures, the inclination of the chemist has also been evidenced toward ACE2 and TMPRSS2 as important targets for the design of anti-COVID19 drugs. Encouragingly, attempts to integrate COVID19 drug discovery campaigns with artificial intelligence-based approaches have also been made. Also, researchers have exhaustively exercised the “smart approach” of not initiating from scratch for drug discovery and shifted to a fast track mode (drug repurposing) to pinpoint anti-COVID19 scaffolds. In numerous studies, the team had a clear plan to commence with the task of anti-COVID19 scaffold identification by utilizing the already existing drugs as viable starting points. Thus, the strategy of drug repurposing was comprehensively leveraged to identify anti-COVID19 scaffolds. The expected reduction in research and development costs, as well as drug development timeline, clubbed with the availability of information about their pharmacology, dosage, possible toxicity and formulation were the main reasons behind the initiation of numerous drug repurposing based endeavours. Resultantly, a plethora of SARS-CoV2 entry as well as replication inhibitors were identified through several high-throughput drug repurposing assays [23, 26, 237, 238]. Important to mention that the FDA approvals of Lagevrio (molnupiravir, Small*-*molecule nucleoside) and Paxlovid (nirmatrelvir and ritonavir, protease inhibitors) exerted a catalytic influence in spurring the researchers to commence with more programs for the generation of small molecule inhibitors (new drug design strategies and repurposing of small molecule inhibitors). Overall, the medicinal chemist has gone an extra mile in generating numerous promising anti-COVID19 scaffolds by employing an expedited drug discovery approach involving iterative cycles of structure design, synthesis, and profiling to generate anti-COVID19 scaffolds.

**Fig. 3:**
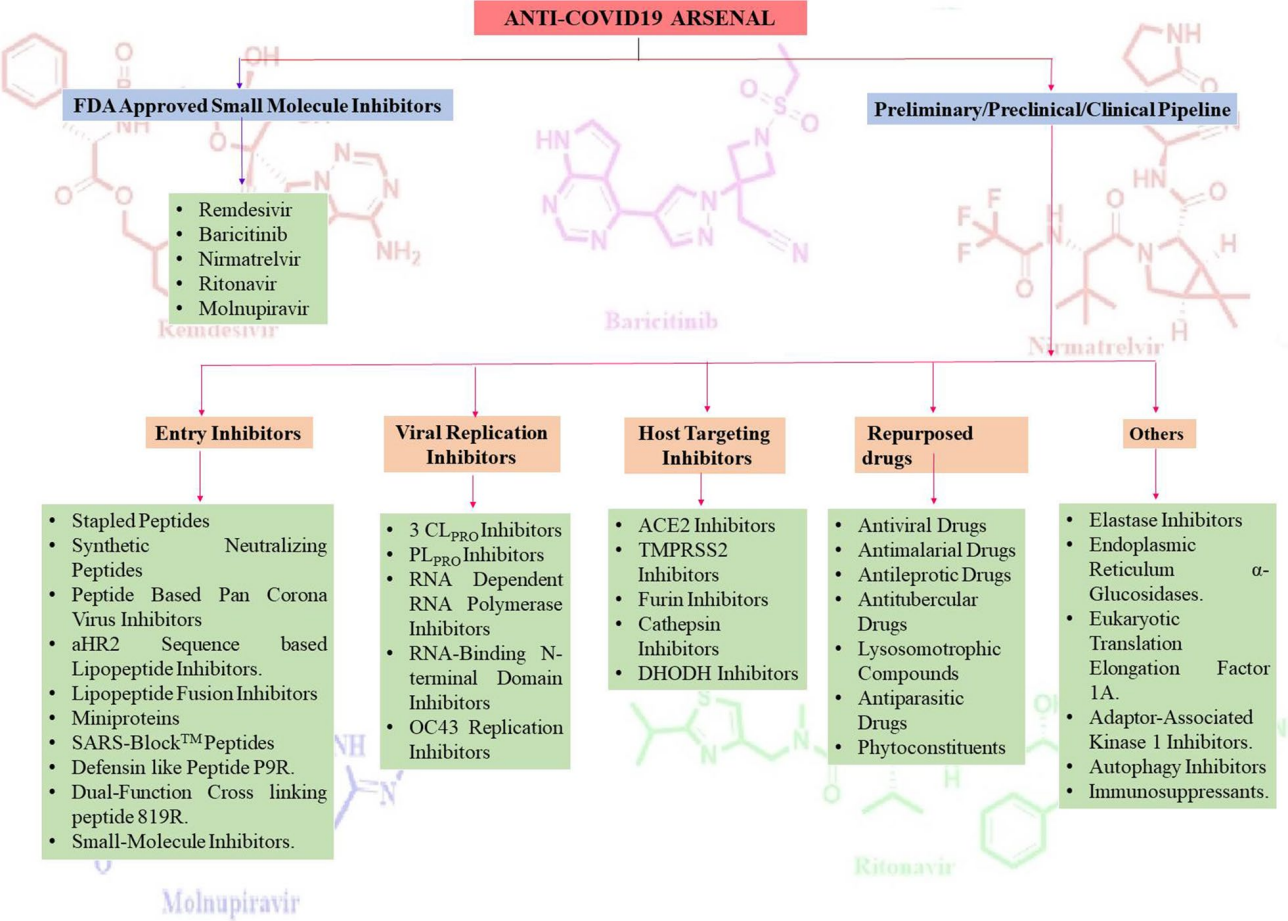
A glance at the anti-COVID19 arsenal

This review article presents an overview of small molecule-based anti-COVID-19 drugs generated through medicinal chemistry campaigns. Encouragingly, stupendous efforts of the interdisciplinary teams in this direction of research have led to the creation of a compendium of several classes of inhibitors targeting S protein, PLpro, 3CLpro and RdRp as well as scaffolds demonstrating the potential to interact with the host proteins, such as ACE2 and TMPRSS2 (Fig. 3). In addition, cathepsin inhibitors for the prevention of viral entry and immunomodulatory drugs were also furnished. Pleasingly, some of the scaffolds have demonstrated the promise to emerge as therapeutics in the future. The compilation also encompasses a detailed discussion of the robust drug design strategies that were employed for the scaffold fabrication to address the potential targets for COVID-19. The key notions regarding the established structure–activity relationship studies ascertaining the impact of structural variation on entry or replication of the virus are also presented. In addition, sections on drug repurposing as well as peptides-based entry inhibitors are also covered. In light of the optimistic findings of the aforementioned drug discovery campaigns, a fast-track expedited approach is required to be employed for the commencement of additional programs centred on the development of small molecule and peptide-based therapeutics for the treatment of COVID-19. There is no denying the fact that the small molecules are being considered underdogs hitherto in this fight (small molecule versus vaccines), however, the evidenced potential of small molecule drugs to approach conserved proteins required for the viral replication of all coronaviruses indicates that they might be able to tackle the mutant SARS-CoV-2 strains [15].

## Protease inhibitors

The causative virus of COVID-19, SARS CoV-2, has striking genetic similarity to SARS CoV-1 in the context of two overlapping large polyproteins that gets cleaved by 3CL_PRO_ at specific sites. For coronavirus replication, this cleavage by 3CL_PRO_ is an imperative post-translation processing step. Notably, the catalytic domain for the protein cleavage (CoV-1 and CoV-2–3CL_pro_ sequences) is identical [44]. Indeed, a report by Dai et al. revealed that the crystallographic information for SARS CoV-1 3CL_PRO_ can be leveraged by the medicinal chemist to design 3CL protease inhibitors as antiviral agents for SARS-CoV-2 [45]. The existing cysteine protease inhibitors can be delved into two categories: covalently bound reversible and covalently bound irreversible inhibitors. Inhibitors of the former class require the presence of electrophilic carbon that reversibly interacts with the sulphur atom of an active site cysteine. This interaction leads to the formation of a covalently bound tetrahedral complex. Forth, as per the studies conducted for the exploration of some inhibitors (X-ray crystallography and NMR), this charged protein–ligand transition state gets stabilized due to the oxyanion hole that exists in the active site [46, 239]. Several functional groups have been utilized for the construction of covalently bound reversible inhibitors such as aldehydes, ketones, nitriles, cyclic ketones, thio- or oxymethylketones, 1, 2-dicarbonyl motifs and amidomethylketones, [44]. To furnish the latter class (covalently bound irreversible inhibitors), chemically reactive tags such as chloromethylketones have been employed. Albeit being quite effective in terms of potent protease inhibition, their high chemical reactivity hinders their therapeutic growth owing to safety concerns [47]. To accelerate the scaffold discovery program of the irreversible type inhibitors, some other chemical classes have also been explored such as acyloxymethylketones which were found to be endowed with low chemical reactivity and high potency toward the enzyme inhibition [48, 240].

**Fig. 4:**
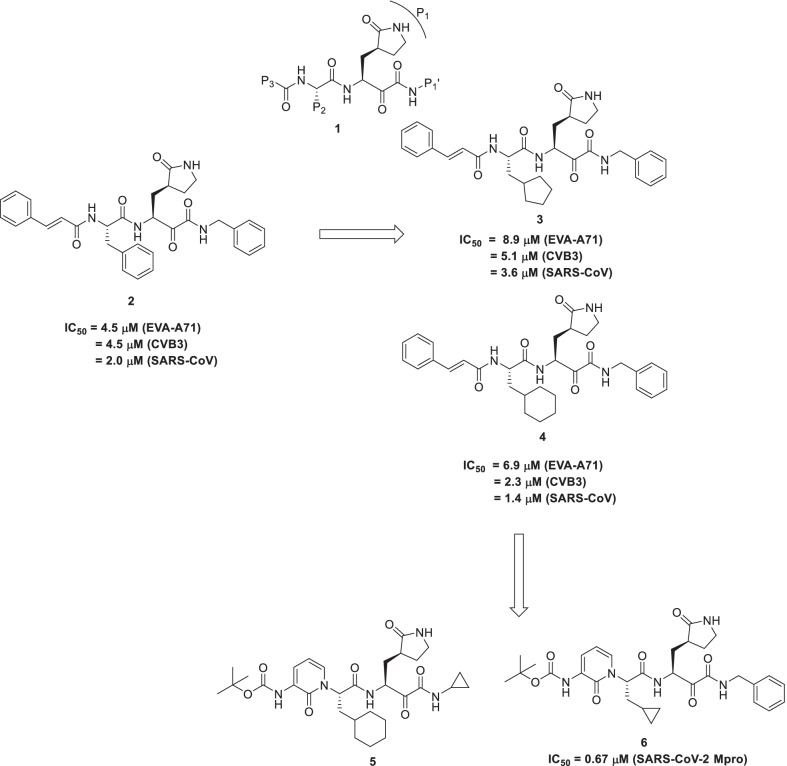
Peptidomimetic α-ketoamides as 3CLpro inhibitors

Recently, Zhang et al. reported the structure-based design of peptidomimetic α-ketoamides as inhibitors of main and 3C proteases. The scaffold construction campaign (**General structure, 1**) commenced with the synthesis of a chemical architecture **2** composed of a cinnamoyl N-cap (P3 position), a benzyl group (P2 position), the glutamine lactam (P1 position) and benzyl (P1′ position). Observation of the crystal structure of compound **2** in a complex with SARS-CoV Mpro, HCoV-NL63 Mpro, and CVB3 3Cpro) revealed that α-keto-carbon was covalently linked to the active-site Cys of the protease. Resultantly, it was observed that contrary to the S configuration in the CVB3 3Cpro complex, the thiohemiketal was in the R configuration in the SARS-CoV and HCoV-NL63 Mpro. This difference in configuration was attributed to the interactions of the oxygen atom. In the SARS-CoV and HCoV-NL63 enzyme, the oxygen atom (thiohemiketal) accepts a hydrogen bond from the main chain amides of the oxyanion hole, however, it accepts the hydrogen bond from the catalytic His40 in the CVB3 protease. Forth, **adduct 1** was fine-tuned structurally to accomplish more potent enzyme inhibitors. It is noteworthy to mention that the five-membered lactam ring was a consistent structural feature of all the compounds and structural alterations were only directed towards P1’, P2 and P3 vectors. The reason for the inclusion of the aforestated structural feature in all the compounds of the series was its good glutamine mimicking ability. Moreover, precedential shreds of evidence indicate that the use of this rigid ring amplifies the potential of the enzyme inhibitors, approximately 10 folds higher than the flexible glutamine side chain. These notions justify the use of a rigid 5-membered lactam ring as P1 residue in the designed ketoamides. Thus, for the structural engineering attempts, the size of P2-Alkyl substituents along with ring size and flexibility of P2-Cycloalkylmethyl substituents was varied. The resulting scaffolds were evaluated against the recombinant proteases as well as in viral replicons and virus-infected cell cultures. Delightfully, the structural optimization attempts of the P2 substituent of the α-ketoamides proved to be beneficial as **3** and **4** were pinpointed as potent scaffolds endowed with equipotency (low µM EC_50_ values) against enteroviruses, alphacoronaviruses, and betacoronaviruses in cell cultures. Noteworthy to mention that compound **4** demonstrated a magnificent activity profile (3-digit picomolar activity) against the MERS-CoV. In light of the striking similarity between the main proteases of SARS-CoV and the novel BetaCoV/Wuhan/2019, it is anticipated that compound **4** might replicate the substantial activity trends against the new coronavirus as well (Fig. 4) [49].

Zhang et al. continued their drug discovery program in this direction in pursuit of improving the half-life of the compound in plasma. To accomplish the aforementioned, the research group carried out a structural engineering program by grafting a pyridone ring in place of the P3-P2 amide bond. It was assumed that this structural alteration will hinder the access of cellular proteases towards this bond, thereby preventing its cleavage. Also, the hydrophobic cinnamoyl moiety was replaced by the less hydrophobic group in a quest to increase the solubility of the compound in plasma and reduce the binding to plasma proteins. These efforts culminated in the identification of compound **5** which demonstrated markedly improved plasma half-life as well as plasma solubility in comparison to **4**. Another structural engineering attempt to improve the antiviral activity against betacoronaviruses of clade b (SARS-CoV-2 and SARS-CoV) was carried out by replacing the P2 cyclohexyl moiety of **5** with a smaller cyclopropyl ring in **6**. Exhaustive explorations of **6** revealed that it inhibits the purified recombinant SARS-CoV-2 M_pro_ with IC_50_ = 0.67 ± 0.18 μM. Pharmacokinetic investigation of both the inhibitors revealed pronounced lung tropism. Moreover, both the inhibitors (**5** and **6**) were suitable for administration by the inhalative route (Fig. 4) [50].

**Fig. 5:**
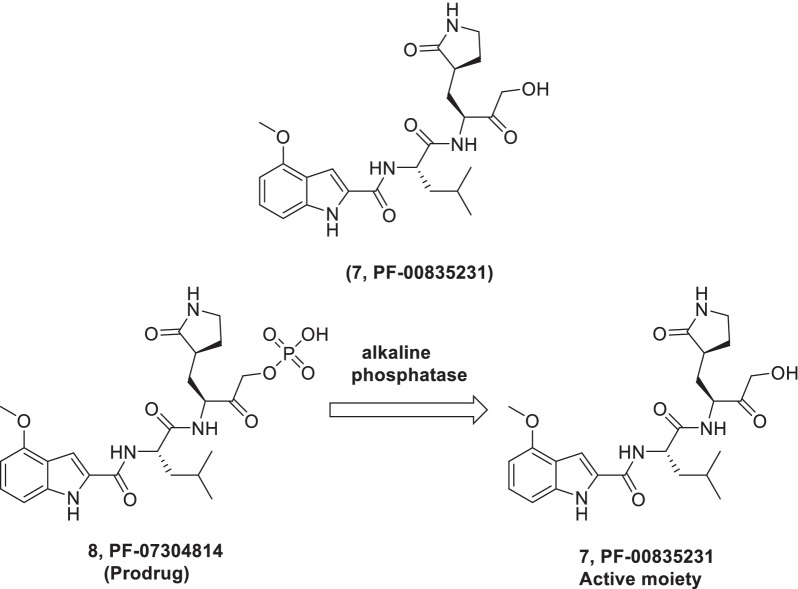
Identification of PF-00835231 as 3CLpro inhibitor

A study was carried out for the discovery of inhibitors (reversible and irreversible, ketone-based) of SARS 3CLpro. Resultantly, a hydroxymethylketone (**7, PF-00835231**) was identified as a potent chemical tool exhibiting potent SARS CoV-1 inhibition in 3CLpro and antiviral assays. Pleasingly, inhibitor **7** was found to be endowed with significant inhibitory potential towards SARS CoV-2 3CLpro along with appropriate pharmaceutical properties that warrant its further investigation for development as an anti-CoV drug for COVID-19 [44]. Lately, detailed preclinical characterization of **7 (PF-00835231)** was carried out by Boras et al. In addition, the research group also synthesized a phosphate prodrug (**PF-07304814**) of **7** in pursuit of attaining sustained systemic exposure in humans. The outcome of the detailed investigations disclosed that **7** is involved in specific binding to SARS-CoV- 2 3CL in vitro and demonstrates broad-spectrum inhibitory activity against a panel of coronavirus 3CLpro. Also, **7** was evaluated for its antiviral efficacy against SARS-CoV-2 in cell culture, utilizing a cytopathic effect (CPE) assay (VeroE6 kidney cells -VeroE6-enACE2 or VeroE6- EGFP). Overall, it was observed that **7** (PF-00835231) was potent enough as a single agent to manifest in vitro antiviral activity against SARS-CoV-2 and also demonstrated synergistic efficacy in combination with remdesivir. Delightfully, in-vivo antiviral activity was also exhibited by **7** when studies were performed in mouse models of SARS-CoV and SARS-CoV-2 infection. Furthermore, the pharmacokinetics studies revealed that the phosphate drug gets rapidly converted to a 3CLpro inhibitor (**7, PF-00835231**). Furthermore, the phosphate prodrug (**8**) delivered as a continuous infusion (500 mg) over 24 h could achieve unbound concentrations of 0.5 µM of the **7 (PF-00835231)**. Overall, the prodrug (**8**) demonstrates a magnificent preclinical pharmacokinetic profile that strongly supported its advancement to clinical stage explorations (Fig. 5) [51, 241].

Another exploration conducted a comparative efficacy analysis of **7 (PF-00835231)** and remdesivir and the results of the investigation revealed that both, **7 (PF-00835231)** as well as remdesivir, were endowed with a magnificent SARS-Cov-2 inhibitory potency in a model of human polarized airway epithelium. Additionally, based on the experiments performed in either A549 cells or human epithelial polarized epithelial cultures, it was observed that the efficacy was not negatively impacted by efflux transporter P-glycoprotein, in either A549^+ACE2^ cells or human polarized airway epithelial cultures [52].

**Fig. 6:**
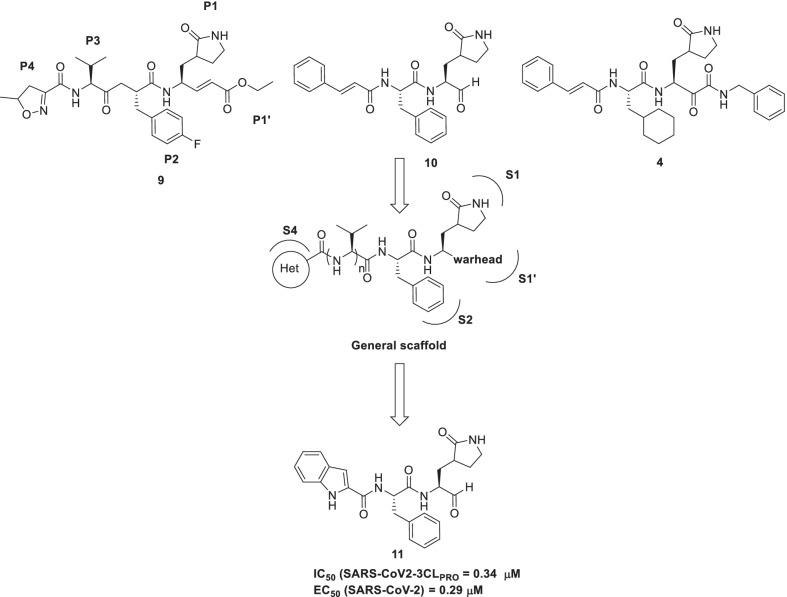
Peptidomimetic aldehydes as 3CLpro inhibitors

Recent exploration by Dai et al. reports the design and synthesis of peptidomimetic aldehydes as inhibitors of 3C protease (3Cpro) of enterovirus 71 (EV71). The design strategy involved careful consideration of the structural features of **9** (**AG7088, rupintrivir**), a peptidomimetic antiviral drug for the treatment of rhinovirus infection. Analysis of the crystal structure of EV71 3Cpro with **9** revealed key interactions/notions such as (i) covalent linkage of the α,β-unsaturated ester with the Cys147 residue (S1 subsite of EV71 3Cpro) ii) hydrogen bonding interaction of the lactam ring with Thr142 and His161 (S1 subsite) iii) occupancy of S2 subsite by substituted phenyl group (P2 position) iv) solvent exposed isopropyl fragment (P3 moiety) v) hydrogen bonding interaction of oxazole ring (P4 moiety) with Gly164, Asn145, and Ser128. In addition to the interaction profile of **9 (AG7088)**, literature precedents also indicated that **9** displays poor plasma stability owing to easy hydrolysis of α, β-unsaturated ester functionality and a short half-life (< 2 min in rat plasma). Another disappointing revelation was the inactivity of **9 (AG7088)** towards SARS-CoV 3CL protease (IC_50_ > 100 μM). With this information in hand, the authors embarked on a drug discovery campaign to furnish peptidomimetic-based inhibitors of enterovirus and SARS-CoV‑2 in pursuit of outwitting the limitations of **9**. To further the aforementioned pursuit, the authors conducted a structural analysis of **9** as well as previously reported **10** [53] and **4** [49] and it was deduced that the three scaffolds (**9**, **10**, **4**) comprised similar key fragments (warhead and (S)-γ-lactam ring). The generated inhibitors were subjected to biological evaluation that led to the identification of potent enzyme inhibitor **11** demonstrating broad-spectrum anti-viral activity against a panel of enteroviruses and rhinoviruses. The analysis of the crystal structure of EV71 3Cpro in complex with compound **11** revealed that the aldehyde group of compound **11** was involved in covalent linkage with the catalytic Cys147. Delightfully, the inhibitor **11** demonstrated inhibitory potential against the 3CLpro and the replication of SARS-CoV-2 (IC_50_ = 0.034 μM, EC_50_ = 0.29 μM). Overall, compound **11** exhibited a superior anti-SARS-CoV-2 as well as pharmacokinetic profile than **9** (**AG7088**) (Fig. 6) [54].

**Fig. 7:**
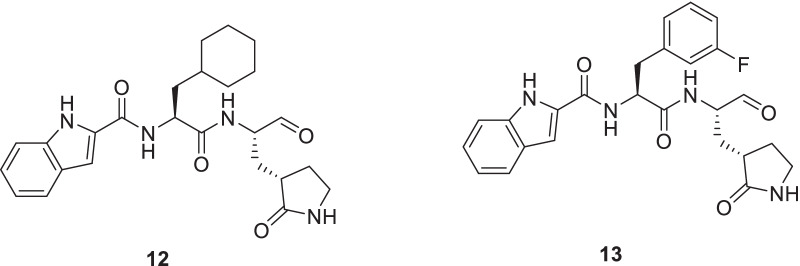
Rationally designed Mpro inhibitors

Recently, Dai et al. furnished Mpro targeting compounds that demonstrated substantial anti–SARS-CoV-2 infection activity (IC_50_ = 0.53 and 0.72 µmol/L). Both the compounds were endowed with low toxicity and appropriate pharmacokinetic properties in vivo. Careful observation of X-ray crystal structures of SARS-CoV-2 Mpro in complex with the compounds revealed covalent binding of the aldehyde groups of **12** and **13** to cysteine 145 of Mpro (Fig. 7) [45].

**Fig. 8:**
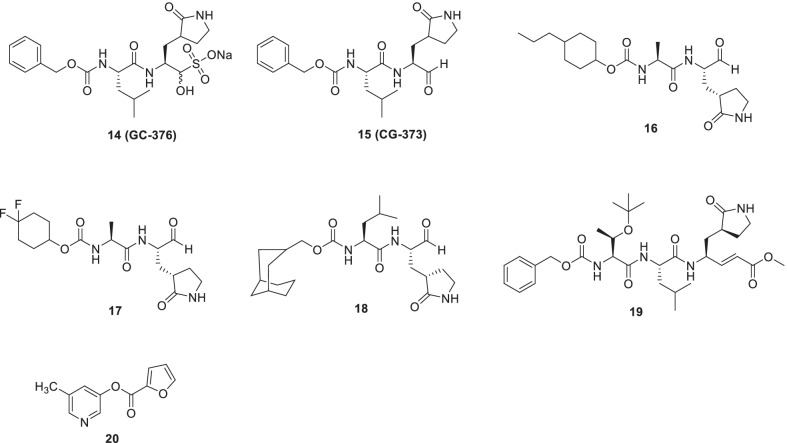
Dipeptidyl-based 3CLpro inhibitors and related scaffolds

Rathnayake et al.conducted a structure-guided optimization endeavour to furnish coronavirus 3CLpro inhibitors [55]. Earlier, the group reported dipeptidyl and tripeptidyl-based 3CLpro inhibitors of multiple human and animal coronaviruses. Given the results of pharmacokinetic and pharmacodynamics studies of the aforementioned chemical classes, it was deduced that dipeptides were pharmacokinetically superior to tripeptides [56, 242]. Notably, a prototype scaffold of the dipeptide series, **14** (GC376), is currently undergoing clinical investigations in animal models of coronavirus infection. As, such **14** is a prodrug, that has demonstrated significant potential to treat feline infectious peritonitis. In a study by Vuong et al., **14** and its active moiety GC373 (**15**), were found to be effective inhibitors of Mpro (SARS-CoV and SARS-CoV-2). Pleasingly, the prodrug, as well as the parent compound exerted their inhibitory effects at the nanomolar concentration (IC_50_ values). In pursuit to gain mechanistic insights, the SARS-CoV-2 Mpro crystal structures with adducts **14** and **15** were investigated at 2.0 and 1.9 Å, and a covalent modification of the nucleophilic Cys145 with the inhibitors was observed. Furthermore, the reversible formation of a hemithioacetal was found to be responsible for the inhibitory effects [57]. With this information in hand, new dipeptidyl series comprising structural variation in the cap substructure was designed. Resultantly, the efforts led to the identification of a promising compound (**16**) endowed with substantial activity against SARS-CoV-2 3CLpro (IC_50_ = 0.17 µM—FRET enzyme assay and EC_50_, 0.15 μM—cell-based assays). Another compound, **17**, exhibited significant potent antiviral activity against MERS-CoV (EC_50_ value = 0.04 μM). Further exploration conducted in a mouse model of MERS-CoV infection revealed that treatment with compound **17** exerted a reduction in lung viral load and also increased the survival of infected mice. Noteworthy to mention that a significant increase in mouse survival was only evidenced when **15** was administered to mice at 1 dpi (Fig. 8) [57].

Spurred by the aforementioned revelations, a drug discovery endeavour centred on dipeptidyl-based inhibitors was initiated by Dampalla et al. [58] The design strategy involved the use of a P1 glutamine surrogate, a P2 Leu residue and an aldehyde warhead. The scaffolds were designed after an in-depth investigation of available X-ray crystal structures of the protease with the previously reported inhibitors [59, 243, 244]. Specifically, transition state SARS-CoV-2 3CLpro inhibitors (masked aldehyde inhibitors as well as nondeuterated and deuterated dipeptidyl aldehydes) comprising of a conformationally constrained cyclohexyl fragment were constructed. Forth the successful furnishment of the designed adducts, their profiling as inhibitors of SARS-CoV2 3CLpro was done in biochemical and cell-based assays. Delightfully, several potent inhibitors of MERS coronavirus 3CLpro were pinpointed, with compound **18** demonstrating a magnificent activity profile in the context of inhibitory potential towards SARS-CoV-2 in vero cells with EC_50_ = 0.035 µM. In addition, compound **18** displayed appropriate cellular permeability as well as a lack of cytotoxicity (Fig. 8) [58].

Iketani et al. recently conducted a study to identify SARS-CoV-2 inhibitors. Resultantly, compounds **14 (GC-376)**, **19** and **20** (MAC-5576) were identified as potent SARS-CoV-2-3CL inhibitors with IC_50_ values of 151, 160 and 81 nM, respectively. The results of the CPE reduction assay revealed that the viral infection in Vero-E6 cells could be blocked by **14** (IC_50_ = 2.883 µM) and **19** (IC_50_ = 2.189 µM), however, **20** did not demonstrate this ability. Furthermore, structures of compounds **14**, **19** and **20** confirmed that all three are covalent inhibitors. Though covalent linkage of **20** to Cys145 was observed, **20** could not exert time-dependent inhibition indicating that it might be a reversible covalent inhibitor (Fig. 8) [60]

**Fig. 9:**
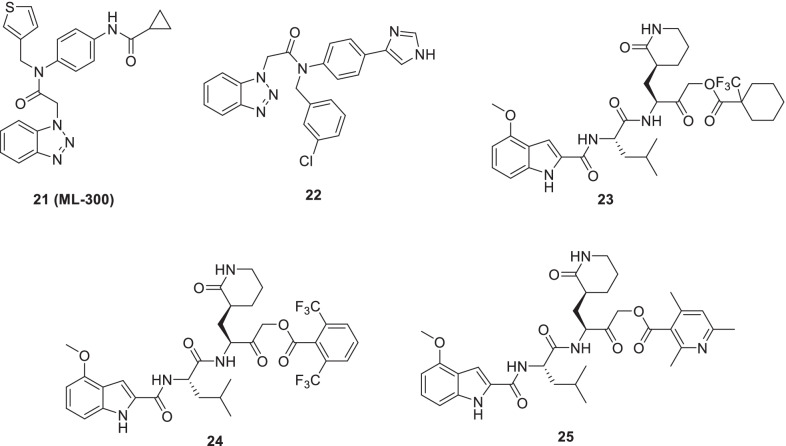
Noncovalent small molecule and α-acyloxymethylketone warhead based peptidomimetic scaffolds as 3CLpro inhibitors

Han et al.initiated a medicinal chemistry optimization program leveraging the chemical architecture of previously reported non-covalent inhibitor **21 (ML300)** for the development of scaffolds that can target SARS-CoV-2 3CLpro [61]. Notably, **21** was identified earlier by Turlingtone et al. as a part of their endeavours centred on the generation of SARS-CoV 3CLpro inhibitors [61]. To steer the progress of this study, the authors studied the X-ray structures of SARS-CoV- 1 and SARS-CoV-2 3CLpro enzymes in a complex with **21** (**ML300**) and **ML300** derived inhibitors which indicated a unique induced-fit reorganization of the S_2_–S_4_ binding pockets. Forth the successful accomplishment of the designed adducts, their assessment was carried out employing a SARS-CoV-2-infected Vero E6 cell viability assay and a plaque formation assay. Resultantly, a non-covalent small molecule SARS-CoV-2 inhibitor **22** was identified that demonstrated a promising activity profile [low nanomolar biochemical inhibition and efficacy in cellular models, EC_50_ (SARS-Cov2 3CLpro) = 68 nM, EC_50_ = 497 nM (anti-viral CPE inhibition), EC_50_ = 558 nM (plaque reduction)]. Notably, the efficacy of compound **22** was comparable to remdesivir. Further explorations are being conducted on compound **22** to improve the DMPK profile as well as biochemical and cellular efficacy (Fig. 9) [62].

Bai et al. recently leveraged six-membered lactam glutamine mimic as a structural motif for the design of α-acyloxymethylketone warhead peptidomimetic scaffolds. Pleasingly, the compounds **23–25** were pinpointed to be potent inhibitors of SARS-CoV-2 3CLpro. Specifically, the compound **23–25** inhibited SARS-CoV2 3CLpro with IC_50_ values of 86, 1 and 19 nM respectively. Also, the aforementioned compounds were endowed with selectivity for SARS-CoV-2 3CLpro over CatB and CatS. The results of the further analysis made several key disclosures such as (i) superior activity profile of compound **23–25** (in vitro SARS-CoV-2 antiviral replication inhibition) in comparison to previously reported peptidomimetic inhibitors (ii) formation of the covalent adduct (cocrystallization of **25** with SARS-CoV- 2 3CL protease) (iii) potential of compound **23–25** to inhibit alphacoronavirus as well as non-SARS betacoronavirus strains. Delightfully, compounds **23–25** also demonstrated a low cytotoxicity profile coupled with good plasma and glutathione stability (Fig. 9) [63].

**Fig. 10:**
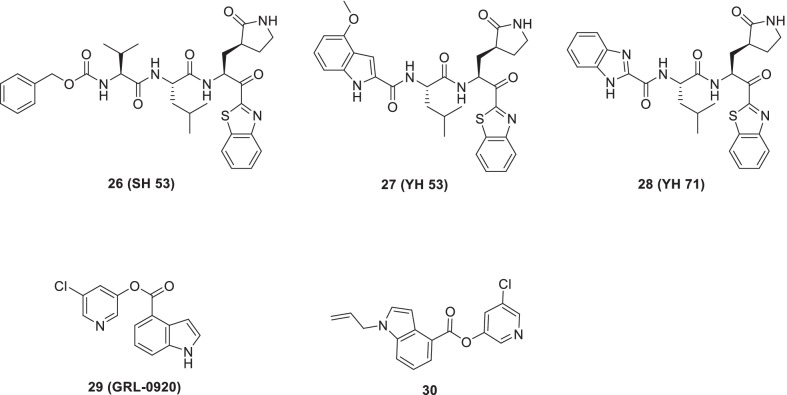
Benzothiazolyl and indole-based 3CLpro inhibitors

In continuation of their drug discovery program to design SARS-CoV-2 3CL_PRO_ inhibitors, Konno et al. conducted another scaffold construction program to furnish anti-SARS-CoV-2 agents [64]. Inhibitors **26 (SH-5**), **27** (**YH-53**), and **28** (**YH-71**) endowed with substantial SARS-CoV-1 3CLpro inhibitory activities with Ki values of 4.1, 6.3 and 22 nM respectively were identified through their previous efforts [65, 245]. The chemical architecture of all the three inhibitors contained benzothiazolyl ketone as the P1-directed warhead. Further analysis revealed the formation of the reversible covalent bond between the electrophilic ketone warhead and nucleophilic thiolate of the active site Cys145 in SARS-CoV-1 3CLpro. Notably this interaction led to transient inactivation of enzyme via the formation of hemithioketal intermediate [65, 245]. In light of the disclosures regarding structural similarity of 3CLpro between SARS-CoV-1 and SARS-CoV-2, the research group in this study explored the potential of their previously reported SARS-CoV-1 inhibitors as SARS-CoV-2 inhibitors. Gratifyingly, compounds **26–28** demonstrated significant SARS-CoV-2 3CLpro inhibition with Ki values of 14.5, 34.7 and 32.1 nM, respectively. Noteworthy to mention that amongst all the three compounds, the structural template of **27** (YH-53) was found to be appropriate for consideration as a lead compound for the development of anti-COVID-19 drug by virtue of its SARS-CoV2 inhibitory activity coupled with favourable in vitro ADME profile as well as in vivo pharmacokinetics. Further investigation on **27** indicated the formation of several hydrogen bonds with the backbone amino acids. Also, a covalent bond was observed in the active site of 3CLpro (Fig. 10) [64].

Hattori et al. recently evaluated indole-chloropyridinyl-esters for their inhibitory activity towards SARS-CoV-2. Specifically, the authors employed VeroE6 cells and TMPRSS2-overexpressing VeroE6 cells for the cell-based assays. Pleasingly, one of the compounds **29**, GRL-0920, demonstrated magnificent activity against SARS-CoV-2 (EC_50_ = 2.8 μM). Also, infectivity, replication and CPE of SARS-CoV-2 were reduced by treatment with compound **29**. Computational studies revealed key interactions of indole and chloropyridinyl fragments with two catalytic dyad residues of Mpro, Cys145 and His41 leading to covalent bonding (Fig. 10) [66]

Recently, Ghosh et al. furnished a series of indole 5-chloropyridinyl esters and evaluated their efficacy as anti-SARSCoV-2 3CLpro inhibitors. The results of the biological evaluation revealed that several compounds could exert their 3CLpro inhibitory potential at low nanomolar concentrations. Mechanistic studies indicated that the ester carbonyl functionality of the inhibitor was prone to the attack of catalytic Cys145. Also, a covalent bond was formed between Cys145 and the carbonyl group of the active ester. SAR studies indicated the position of the carboxylic acid on the indole ring was critical for activity. Encouragingly, the outcome of immunocytochemistry assays as well as Vero E6 cell-based assays also confirmed the magnificent antiviral activity profile of numerous compounds. Specifically, compound **29** exhibited striking SARS-CoV-2 3CLpro inhibitory potential [IC_50_ = 250 nM, EC_50_ = 2.8 μM (VeroE6 cells)]. Notably, **29** demonstrated comparable antiviral activity as that of remdesivir in immunocytochemistry assays. Another compound **30** displayed an impressive activity profile with IC_50_ value 73 nM (SARSCoV-2 3CLpro) (Fig. 10) [67].

**Fig. 11:**
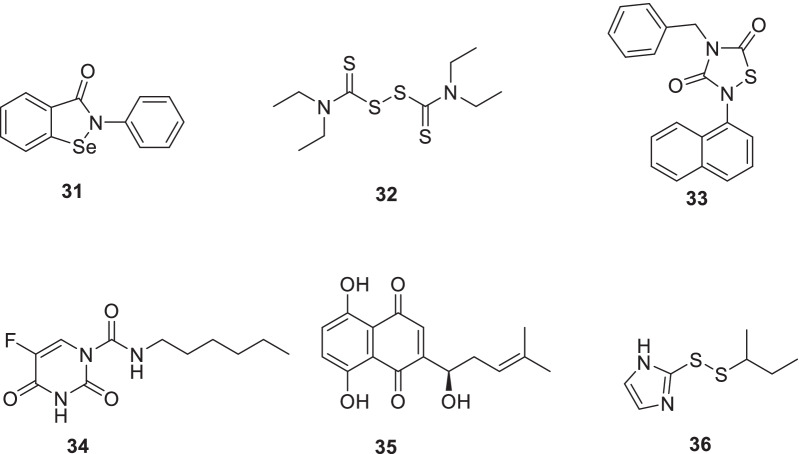
3CLpro inhibitors

Ma et al.explored the mechanism of previously reported M^pro^ inhibitors. Specifically, **31** (ebselen), **32** (disulfiram), **33** (tideglusib), **34** (carmofur), **35** (shikonin), and **36** (PX-12) were investigated mechanistically employing native mass spectrometry, molecular dynamics simulations, FRET-based enzymatic assay, cellular antiviral assays and thermal shift assay. All the six inhibitors demonstrated nonspecific Mpro inhibition and the addition of 1, 4-dithiothreitol attenuated the inhibitory activity of the compounds. Delightfully, the compounds also exhibited inhibitory activity towards SARS-CoV-2 PLpro and 2A^pro^ and 3C^pro^ from enterovirus A71 (EV-A71) and EV-D6 (Fig. 11) [68].

Spurred by the SARS-CoV-2 Mpro inhibitory potential of **34** (carmofur), the X-ray crystal structure of Mpro in complex with **34** was studied. Resultantly, some key insights were obtained such as covalent binding of carbonyl reactive group of **34 (carmofur)** to catalytic Cys145, and hydrophobic S2 subsite occupied by the fatty acid tail (Fig. 11) [69].

**Fig. 12:**
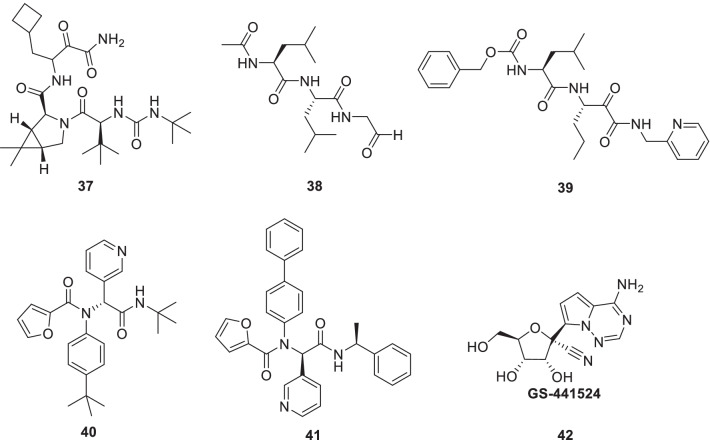
Boceprevir, calpain inhibitors II and calpain inhibitors XII and other scaffolds as 3CLpro inhibitors

Ma et al. employed a FRET-based enzymatic assay and reported **37** (boceprevir), **38** (calpain inhibitor II) and **39** (calpain inhibitor XII) as novel chemotypes targeting the SARS-CoV-2 Mpro. Notably, significant inhibition of SARS-CoV-2 viral replication was evidenced by **37–39** (EC_50_ = 0.49–3.37 µM) [70]. The aforestated disclosures shed light on the fact that unlike most of the Mpro inhibitors containing a γ-lactam glutamine surrogate (P1 position), **38** and **39** comprise hydrophobic moieties at the P1 site. Notably, **38** and **39** also manifest activity against a host protease, cathepsin that plays a key role in the viral entry. Based on the above-mentioned, dual inhibition of Mpro and cathepsin L is presently being considered a prudent approach for the generation of SARS-CoV-2 antivirals. [71]. Another study reported the efficacy of **37** (boceprevir) as well **14** (GC376) in inhibiting the SARS-CoV-2 in vero cells. Moreover, the results also indicated that synergistic efficacy can be attained with the combination of **14** (GC376) and remdesivir (Fig. 12) [72].

Inspired by the activity profile of calpain inhibitors coupled with the revelations regarding the similar binding mode of **40** (ML188) as that of **39** (calpain inhibitor XII), Kitamura et al. rationally designed noncovalent SARS-CoV‑2 M_pro_ inhibitors [73]. As such, **40** (ML188) is a SARS-CoV Mpro inhibitor (IC_50 =_ 1.5 ± 0.3 μM). Also, **40** (ML188) inhibits the SARS-CoV viral replication (EC_50_ = 12.9 μM, Vero E6 cells). With this background, a compendium of ML188 analogs was generated using a structure-based drug design and one-pot Ugi four-component reaction. Resultantly, compound **41** was identified as a potent noncovalent inhibitor that demonstrated striking inhibitory selectivity towards SARS-CoV-2 and SARS-CoV Mpro (EC_50_ = 1.27 μM, Vero E6 cells) but not towards other viral and host proteases. This is an overwhelmingly positive finding as **14** (GC376) does not exhibit this selectivity trend and also inhibits the host cysteine proteases that might lead to side effects. Investigation of the cocrystal structure of SARS-CoV-2 Mpro with **41** revealed a ligand-induced binding pocket between S2 and S4 sites (Fig. 12) [73].

Compounds **37** (Boceprevir), **38** (calpain inhibitors II) and **39** (calpain inhibitors XII) as well as **14** (GC376) have also been evaluated for their SARS-CoV-2 inhibitory potential in other studies. Some imperative disclosures include (i) broad-spectrum antiviral activity of **37**, **38**, **39** and **14** against SARS-CoV, SARS-CoV-2, MERS-CoV and human coronaviruses (229 E, OC43, and NL63) [74] (ii) In-vivo SARS-CoV-2 infection inhibitory efficacy of **14** (iii) reduced inflammation as well as viral loads in SARS-CoV-2-infected K18-hACE2 mice on treatment with **14** [75] (iv) effective protection of mice against SARS-CoV-2 infection with a cocktail of **14** and **42** (GS441524) (low dose) (Fig. 12) [76].

**Fig. 13:**
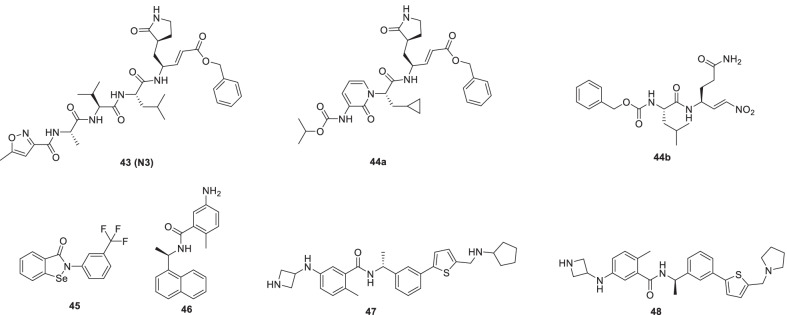
Structure-based drug design of 3Clpro and PLpro inhibitors

Jin et al. employed the strategy of structure-based drug design coupled with HTS and virtual drug screening to embark on a campaign to pinpoint promising scaffolds with the ability to target Mpro of SARS-CoV-2. Resultantly, **31** (Ebselen, organoselenium compound) and **43** (N3, a michael acceptor inhibitor) demonstrated pronounced inhibitory potential SARS-CoV-2 (EC_50_ = 4.67 μM and 16.77 μM). Moreover, both the compounds were endowed with cellular membrane penetrating ability to access their targets (Fig. 13) [77].

Arafet et al. recently simulated the inhibition process of SARS-CoV-2 Mpro with **43** (N3) and utilized the results to design two putative inhibitors **44a** and **44b** of SARS-CoV-2 Mpro. The recognition site of **43** (N3) was varied for the design of inhibitor **44a** and the warhead was modified to furnish the inhibitor **44b**. QM/MM modelling-based mechanistic studies disclosed that compound **44a** is an irreversible inhibitor while **44b** is a reversible inhibitor. Based on these findings, it is imperative to evaluate these inhibitors to ascertain their candidature as anti-COVID drugs (Fig. 13) [78]

Huff et al. investigated the binding mode of **31 (ebselen)** in the active site of Mpro. Utilizing the extracted information, a series of 2-phenyl-1,2-benzoselenazol-3-one analogue was designed and synthesized. Structural variations were particularly directed towards the N-phenyl moiety as well as electronically diverse substitutions and some lipophilic surrogates of the phenyl moiety were explored. Among the synthesized compounds, six compounds were identified through FRET-based screening against recombinant SARS-CoV-2 Mpro that manifested substantial potential to inhibit proteolysis at nanomolar concentrations. Covalent and non-covalent mechanisms were involved in the inhibition of SARS-CoV-2 Mpro as evidenced by the results of preincubation dilution experiments and molecular docking. The most potent compounds **45** magnificently inhibited the viral replication (EC_50_ value-844 nM, SARS-CoV-2-infected Vero E6 cells). Also, the potential of compound **45** to impair SARS-CoV-2 replication in human lung epithelial cells and human induced pluripotent stem cell-derived 3D lung organoids was evidenced (Fig. 13) [79].

Like SARS-CoV-2 cysteine protease, 3CLpro, 2 papain-like protease (PLpro) is also being considered an excellent therapeutic target for COVID-19 owing to its involvement in the regulation of viral replication and reversal of host-mediated post-translational modifications in response to viral infection. Unfortunately, the strategy of inhibiting the covalent modification of active site cysteine that has been exhaustively leveraged for the generation of 3CLpro inhibitors, does not work for the construction of PLpro inhibitory adducts due to the featureless P1 and P2 sites (Gly-Gly recognition). Though **46** (GRL0617) is a noncovalent SARS-CoV PLpro inhibitor (IC_50_ = 1.61 µM), it does not display an impressive activity profile to further the pursuit of its development as an antiviral agent. Nevertheless, the cocrystal of **46** with SARS-CoV PLpro has led to the initiation of several structure-based design endeavours in the recent past. With this information in hand, the structural template of **46** (**GRL0617**) was employed as a starting scaffold by Shen et al. for the design of SARS-CoV‑2 PLpro inhibitors. It was earlier established that the benzamide of **46** was responsible for establishing the hydrogen-bonding network with residues of the BL2 loop. However, the author’s strategy was based on engaging additional binding interactions than that utilized by **46** in the quest to extract amplified inhibitor potency. With this mindset, a set of noncovalent PLpro inhibitors was furnished. Subsequent biological evaluations led to the identification of potent PLpro inhibitors such as **47 (**IC_50_ = 0.39 µM) and **48** (IC_50_ = 0.56 µM) that manifested significant potential to block infection of human cells by SARS-CoV-2 PLpro. Noteworthy to mention that the results of the studies conducted to evaluate the ability of **47** and **48** to reach appropriate plasma concentrations indicated the C_max_ measurements of 6130 ng/mL for **47** and 6403 ng/mL for **48** corresponding to 12 − 13 μM plasma concentrations, respectively. Overall, the aforementioned revelations warrant further investigation of **47** and **48** (Fig. 13) [80].

**Fig. 14:**
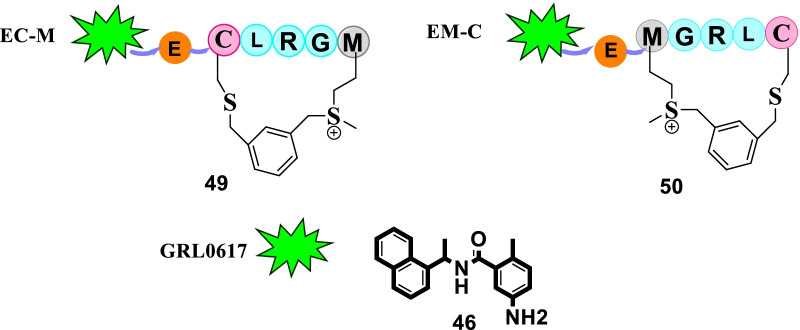
Peptide–drug conjugates as PLpro inhibitors

Spurred by the potential of peptide–drug conjugates (PDCs) to deliver drug payloads, Liu et al. initiated an endeavour to furnish SARS-CoV-2 PLpro targeting PDCs via linking the structural template of **46** (GRL0617) to the sulfonium-tethered peptides derived from PLpro-specific substrate LRGG. The results of the study were overwhelmingly positive as the two adducts (**49** and **50**) demonstrated the ability to covalently label PLpro active site C111 and could also covalently bind to SARS-CoV PLpro and MERS PLpro. Pleasingly, the adducts **49** and **50** were found to be endowed with significant inhibitory potential towards SARS-CoV PLpro with IC_50_ values of 3.43 ± 0.54 and 16.38 ± 0.81 µM, respectively. In addition, the results of the anti-ISG15 immunoblotting assay revealed that the cellular ISGylation level suppressed by SARS-CoV-2 PLpro could be recovered by **49** and **50** in a dose- dependent manner. Notably, **PDC 50** also manifested the potential to recover IFN-β promoter activity on a cellular level. Collectively, it was deduced that the adduct **50** exerts dual effects in the context of reactivating the host’s innate immune response against viral infection as well as interfering with the SARS-CoV-2 replication (Fig. 14) [81].

**Fig. 15:**
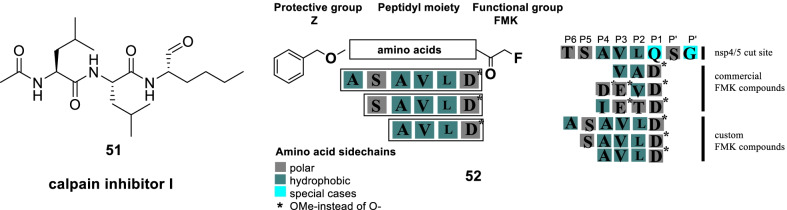
NSP5 (non-structural protein) inhibitors

In the quest to identify SARS-CoV-2 inhibitors, Milligan conducted a high throughput drug screening program to pinpoint NSP5 (non-structural protein) inhibitors. It is well known that the cleavage of polyproteins by Mpro, (encoded by nsp5) releases nsp4–nsp16 and the excised NSPs play an essential role in constituting the viral replication transcription complex. In this context, nsp5 inhibition is presently being conceived as a logical strategy for the design of antiviral drugs. With this information in hand, the authors commenced with their endeavour and developed assays for nsp5 protease activity. A compendium of 5000 characterised pharmaceuticals was evaluated. Resultantly, some promising inhibitors of nsp5 were identified (**51**, calpain inhibitor I and **52**, peptidyl fluoromethyl ketones). Forth, the authors employed a sequence alteration approach for the peptidomimetic FMK inhibitors and furnished potent nsp5 inhibitors (IC_50 =_ 0.16–0.23 µM). Overall, substantial inhibition of viral infection in monkey-derived vero E6 cells was exerted by **51** (calpain I inhibitor, EC_50_ = 0.28 µM). Also, the furnished peptidyl FMK inhibitor displayed pronounced antiviral activity (Fig. 15) [82].

**Fig. 16:**
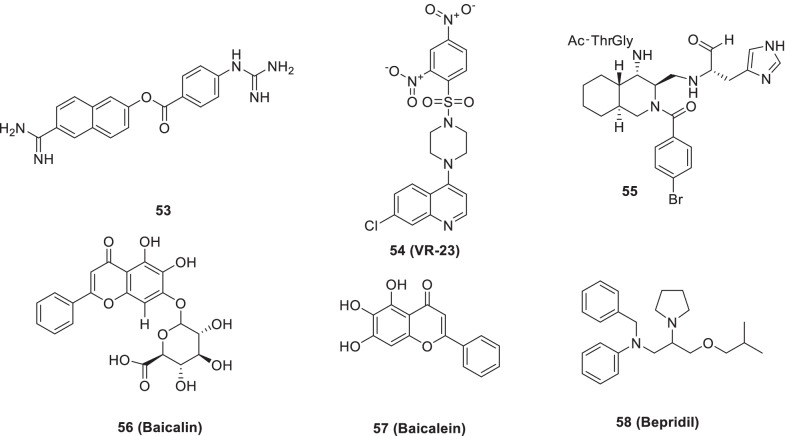
FDA-approved protease inhibitors, decahydroisoquinoline scaffold-bearing adducts, and natural product-based 3Clpro inhibitors

Recently, Bhowmik et al. performed molecular docking of several ligands from the library of protease inhibitors to figure out the binding affinity. Based on docking scores, **53** (Nafamostat) and **54** (VR23, protease inhibitor) were further investigated and it was observed that both the inhibitors were endowed with favourable pharmacokinetic properties. Overall, **53** and **54**, were identified as potential 3CLpro and PLpro SARS-CoV-2 inhibitors for COVID-19 treatment [62]. Also, the dynamics simulation studies highlighted the SARS-CoV-2 3CLpro inhibitory potential of FDA-approved protease inhibitors, paritaprevir and simeprevir (Fig. 16) [83].

Another recent study structurally explored the decahydroisoquinoline scaffold for furnishing non-peptide inhibitors for SARS 3CLpro. The design strategy involved the introduction of a non-prime site substituent at the 4th-position of the decahydroisoquinoline scaffold. The adduct (**55**) was constructed by Pd (II) catalyzed diastereoselective ring formation. Delightfully, the strategy employed yielded **55** with inhibitory activity for R188I SARS 3Clpro (IC_50_ = 26 µM) (Fig. 16) [84].

Vandyck et al. reported the SARS-CoV-2 3CLpro inhibitory potential of **ALG-097111** (structure not disclosed) (IC_50_ = 7 nM) along with its ability to cause reduction of the viral RNA copies and the infectious virus titers in the lungs. Notably, **ALG-097111** selectively inhibited the SARS-CoV-2 3CLpro and did not modulate the functions of human cathepsin L [85].

While investigating the natural product derived from Chinese traditional medicines, Su et al.identified **56 (baicalin)** and **57 (baicalein)** as SARS-CoV-2 3CLpro inhibitors (IC_50_ = 10.27 and 1.69 µmol/L, non-covalent, non-peptidomimetic type inhibitors). Subsequent investigations revealed key insights regarding the binding mode of **57 (baicalein)** with SARS-CoV-2 3CLpro. Compound **57 (Baicalein)** interacted with S1/S2 subsites and the oxyanion loop and prevented the peptide substrate from reaching the active site [86]. Another study result indicated that **57** (**Baicalein**) exerts its effects on SARS-CoV-2 replication via inhibition of mitochondrial OXPHOS in an mPTP-dependent manner. Besides underscoring the potential of **57 (Baicalein)** as a promising drug for COVID-19, the study results present the targeting of mitochondrial OXPHOS/mPTP as a prudent strategy for antiviral drug development [87]. Liu et al. also investigated the anti-SARS-CoV-2 activity of *S. baicalensis* and its ingredients. Resultantly, the major component of *S. baicalensis,* **57** (**baicalein**) was found to be endowed with magnificent SARS-CoV-2 3CL^pro^ inhibitory activity (IC_50_ = 0.39 µM). Experiments also indicated that the ethanolic extract of *S. baicalensis* inhibited the viral entry while **57** (baicalein) acted at the viral post-entry stage. Moreover, some analogues of **57** (baicalein) were also identified to inhibit SARS-CoV-2 3CL^pro^ activity (Fig. 16) [88].

Vatansever et al. evaluated 30 FDA/EMA-approved drugs for their Mpro enzyme (SARS-COV-2) inhibitory potential by employing computational docking analysis. Resultantly, compound **58 (bepridil)** was identified as a strong inhibitor of SARS-COV-2 for entry and replication inside Vero E6 and A549 cells. Also, inhibition of CPE induced by SARS-CoV-2 infection in Vero E6 and A549 cells was caused by **58 (bepridil)** (IC_50_ value = 0.86 and 0.46 µmol/L) (Fig. 16) [89].

**Fig. 17:**
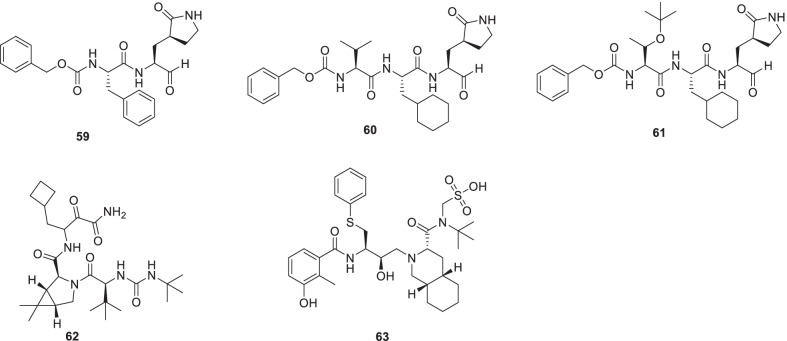
Opal-based dipeptidyl and tripeptidyl-based inhibitors and other scaffolds as 3Clpro inhibitors

Yang et al. embarked on a medicinal chemistry endeavour to generate Opal-based dipeptidyl and tripeptidyl-based inhibitors of SARS-CoV-2 Mpro. β-(S-2-oxopyrrolidin-3-yl)-alaninal (Opal) was strategically placed in the structural template of the designed adducts for the formation of a reversible covalent bond with the SARS-CoV-2 Mpro active-site cysteine C145. Subsequent evaluations led to the identification of **59** as a potent inhibitor of SARS-CoV-2 Mpro (IC_50_ = 8.5 nM). Several other inhibitors also demonstrated significant inhibitory potential toward SARS-CoV-2 induced CPE in Vero E6 and A549/ACE2 cells. Notably, SARS-CoV-2-induced CPE in Vero E6 cells was completely prevented by inhibitors **60** and **61 **(Fig. 17) [90].

In a study by Jan et al., numerous small molecules, as well as traditional herbal medicines, were screened employing a cell-based infection assay. The aforementioned efforts led to the identification of 15 small molecules with anti-infective activity. Two small molecules **62 (boceprevir)** and **63 (nelfinavir mesylate**) displayed promising results as they inhibited SARS-CoV-2 infection and replication with IC_50_ of 50.1 and 3.3 µmol/L. The active compounds were assessed for their 3CL pro targeting potential and further tested in vivo. Resultantly, **62** (**boceprevir**) and **63** (**nelfinavir**) demonstrated the potential to inhibit 3CLpro. Also, in a challenge study using hamsters as a disease model, both the compounds (**62** and **63**) demonstrated efficacy (Fig. 17) [91].

**Fig. 18:**
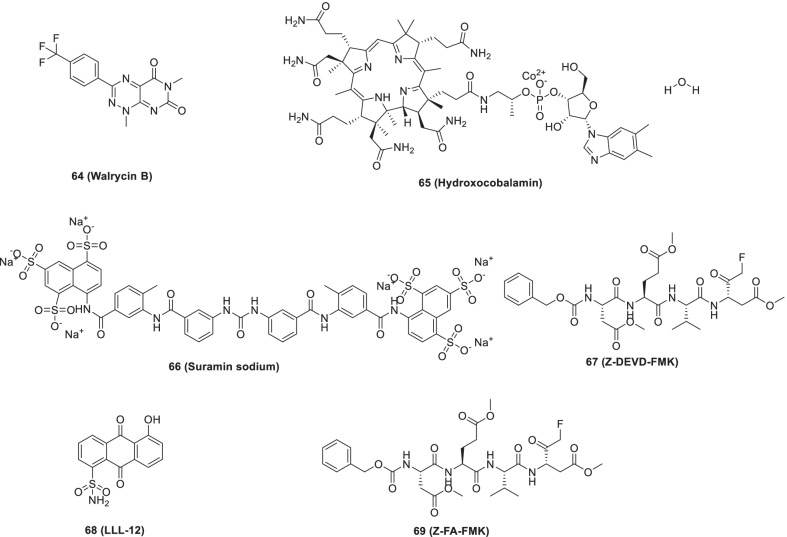
Small molecule inhibitors of SARS-CoV-2 3CLpro identified through a quantitative HTS

Zhu et al. identified 23 small molecule inhibitors of SARS-CoV-2 3CLpro (IC_50_ value = 0.26–28.85 µM) through a quantitative HTS of approved and investigational drugs. The results of the investigation were optimistic as several compounds were found to be endowed with excellent to moderate inhibitory potential. Specifically, **64** (Walrycin B, IC_50_ = 0.26 μM), **65** (hydroxocobalamin, IC_50_ = 3.29 μM), **66** (suramin sodium, IC_50_ = 6.5 μM), **67** (Z-DEVD-FMK, IC_50_ = 6.81 μM), **68** (LLL-12, IC_50_ = 9.84 μM), and **69** (Z-FA-FMK, IC_50_ = 11.39 μM) were found to be the most promising. Overall, these inhibitors can be used in combination with other inhibitors for COVID-19 treatment and can also be used for structural engineering endeavours to amplify the potency (Fig. 18) [92].

**Fig. 19:**
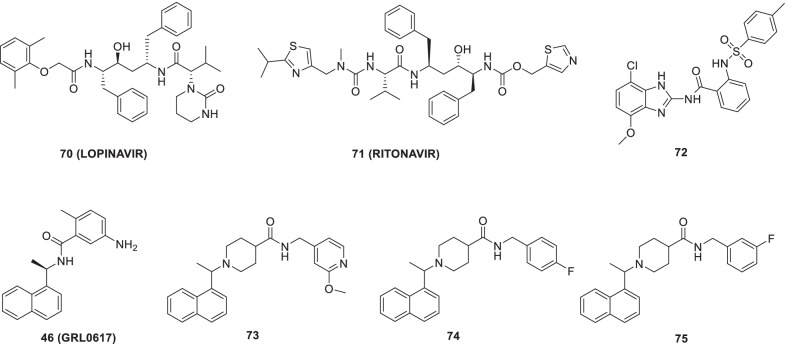
Small molecules as 3CLpro inhibitors

Choy et al. recently reported the efficacy of **70 (lopinavir)** (protease inhibitor) against the SARS-CoV-2 virus in Vero E6 cells (EC_50_ = 26.63 µM) [93]. In a phase 2 trial in COVID-19 patients, the efficacy and safety of the triple antiviral combination of **70 (lopinavir), 71 (ritonavir)** and **ribavirin** with interferon beta-1b were evaluated. The outcome of the trial was overwhelmingly positive as the triple antiviral therapy could reduce the duration of viral shedding in COVID-19 patients. Notably, the triple antiviral therapy was found to be safe (Fig. 19) [94].

In light of the reports of USP2 inhibitors demonstrating SARS-CoV PLpro inhibitory potential, Mirza et al. commenced with an integrated in-silico study to identify potential USP2 inhibitors through integrated in-silico efforts. As a result, compound **72 (Z93)** was identified as a promising candidate that could fit well in the binding site of proteases and was found to be involved in π-π stacking and H-bond interactions. (Fig. 19) [95].

Gao et al. reported the potency of **46** (**GRL-0617**) as SARS-CoV-2 PLpro activity (IC_50_ = 2.2 µmol/L) and also investigated the structure of SARS-CoV-2 PLpro complexed by **GRL0617** (**46)** to 2.6 Å. It was observed that, in addition to occupying the substrate binding pockets, compound **46** also prevented the binding of the substrate by sealing the entrance to the substrate binding cleft [96]. The SARS-CoV-2 PLpro inhibitory potential of **46** (**GRL-0617**) was also reported in another study (IC_50_ = 2.1 µmol/L). The drug demonstrated effective antiviral inhibition of SARS-CoV-2 in cell-based assays. The observation of co-crystal structure of SARS-CoV-2 PLpro^C111S^ by **46 (GRL0617)** revealed the covalent nature of the inhibitor. Moreover, it was found that **46** (GRL0617) resides in the ubiquitin-specific proteases domain of PLpro. The binding of ISG15 C-terminus to PLpro was blocked by **46 (GRL0617)** as indicated by the NMR studies. Notably, the drug was almost free from cytotoxicity on Vero E6 cells up to a concentration of 100 µmol/L (Fig. 19) [97].

Klemm et al. recently reported that non-covalent inhibitor **73** targets SARS-CoV-2 PLpro (IC_50_ = 0.81 µM). The inhibitor **73** also manifested the potential to prevent self-processing of nsp3-GFP indicating that the inhibitor can efficiently target viral replication. A clear-cut reduction of CPE was evidenced by the treatment with **73** (11 µmol/L). Also, two other non-covalent inhibitors, **74** and **75**, were found to inhibit SARS2 PLpro with IC_50_ = 1.4 and 1.15 µM, respectively (Fig. 19) [98].

**Fig. 20:**
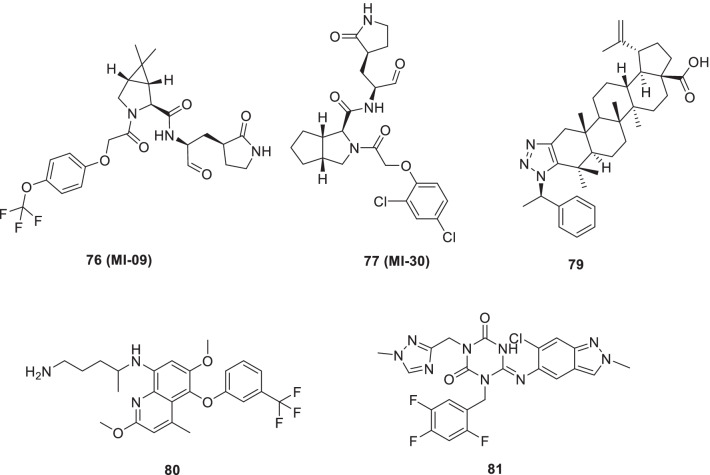
Pragmatically designed 3Clpro inhibitors, nsp15 inhibitors and other scaffolds

Qiao et al. embarked on a drug discovery endeavour and furnished 32 new bicycloproline-containing Mpro inhibitors. Subsequent evaluations led to the identification of two compounds (**76**, MI-09 and **77**, MI-30) endowed with excellent antiviral activity coupled with the ability to reduce the lung viral loads and lung lesions in a transgenic mouse model of SARS-CoV-2 infection. The results of the investigation also revealed that both the compounds downregulated the expression levels of IFN-β and CXCL10, which was assumed to be the reason behind the ability of the compound to ameliorate lung damage. In addition, both the compounds demonstrated appropriate pharmacokinetic properties (Fig. 20) [99].

Investigations conducted in pursuit of identifying drug targets for the design of small molecule inhibitors for COVID-19 revealed that NSP15 of COVID-19, a uridylate-specific endoribonuclease, bears a high sequence similarity with **NSP15** of SARS and MERS and can be leveraged as a prudent target for the furnishment of anti-COVID-19 drugs. Albeit, its functions are not fully understood, NSP15 is reported to be involved in RNA replication and processing of subgenomic RNAs. In the quest to capitalize on these revelations, structure-based drug designing campaigns have been initiated for the identification of NSP15 targeting anti-virals for COVID-19 [100]. A recent study by Stevaert excellently exemplifies one such endeavour and reports 1,2,3-triazolo-fused betulonic acid based CoV nsp15 inhibitors. Efforts invested to establish the structure–activity relationship culminated in the identification of potent inhibitors of HCoV-229E replication (EC_50_ = 0.6 μM). Mechanistic studies were performed using HCoV-229E and it was observed that the inhibitor **79** acts post-entry at an early stage in viral RNA synthesis as evidenced by the results of the time-of-addition experiment. Furthermore, it was observed that the endoribonuclease active site mutant virus was noticeably less sensitive to the exposure of **79**. Mutations in the N-terminal part of HCoV-229E nsp15 were deduced to be the reason for the aforementioned resistance towards compound **79**. Disclosures from the docking study revealed that **79** binds to an inter-monomer nsp15 interface lying adjacent to the EndoU catalytic core (Fig. 20) [101].

Chen et al. recently screened FDA-approved drugs for the treatment of COVID-19 patients employing a FRET-based HTS platform. Resultantly, **80** (tafenoquine), an antimalarial drug, was found to be endowed with the potential to exert conformational change in SARS-CoV-2 Mpro, thereby diminishing the protease activity. Also, the drug significantly inhibited SARS-CoV-2 infection in the cell culture system (IC_50_ = 2.5 µmol/L) [102]. In another study, **80** (Tafenoquine) was found to be more potent than hydroxychloroquine in inhibiting SARS-CoV-2 in VERO E6 cells (Fig. 20) [103].

UNOH et al.identified a non-covalent nonpeptidic SARS-CoV-2 3CLpro inhibitor **81 (S-217622)** employing virtual as well as biological screening protocols. Delightfully, antiviral activity against SARS-CoV-2 variants was exhibited by the inhibitor [IC_50_ (SARS-CoV-2 3CLpro) = 0.13 µM and EC_50_ (VeroE6/TMPRSS2) = 0.37 µM] in the in vitro studies. Moreover, **81 (S-217662)** was also found to be suitable for once-daily oral dosing and also exerted dose-dependent inhibition of intrapulmonary replication of SARS-CoV-2 in mice (Fig. 20) [104].

Recently, Kneller et al. attempted to delineate the neutron structure of Mpro in complex with the protease inhibitor at near-physiological (22 °C) temperature. Specifically, a comparison of the protonation states of the inhibitor complex and ligand-free Mpro was observed. It was found that the binding of the inhibitor resulted in a cascade of protonation/deprotonation and conformational events. The aforementioned events led to the remodelling of the SARS-CoV-2 Mpro active site cavity. Collectively the study results can be leveraged for the design of protease inhibitors endowed with the potential to specifically target the Mpro enzyme from SARS-CoV-2   [105].

**Fig. 21:**
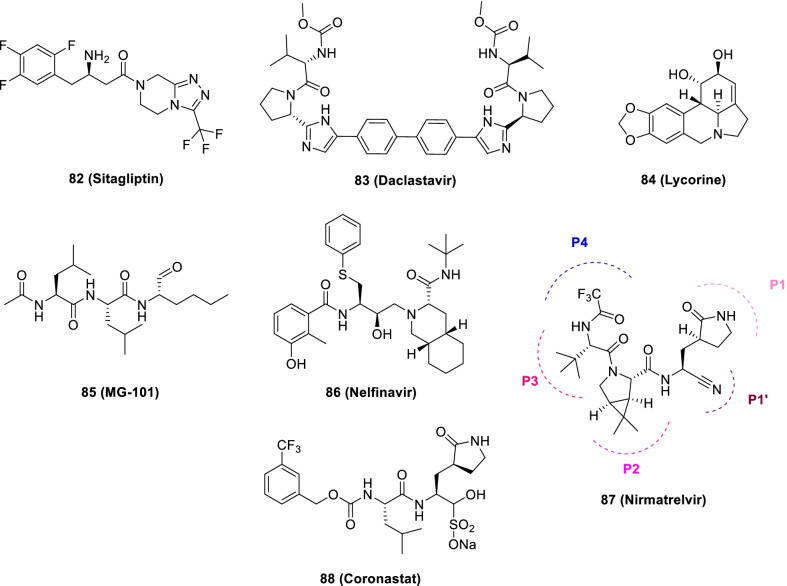
Nirmatrelvir, GC376-based small molecule SARS-CoV-2 3CLpro inhibitors and small molecules identified through deep neural network-based generative and predictive models

Narayanan et al. in pursuit of quick identification of protease inhibitors with low cytotoxicity employed a novel in-cell protease assay. For the study, a library of 64 repurposed drugs was screened which led to the identification of eight compounds with promising anti-SARS-CoV-2 activity. Notably, **82** (**Sitagliptin**) was found to be an effective PLpro inhibitor that decreased SARS-CoV2 in Huh-7.5 cells with an EC_50_ = 0.32 μM and a CC_50_ of 22 μM. Another PLpro inhibitor, **83 (Daclastavir)** also demonstrated the potential to reduce SARS-CoV-2 replication in Huh-7.5 cells. Moreover, compound **84 (Lycorine HCl)** also elicited striking Mpro inhibitory activity with an EC_50_ of 0.01 µM. It was also observed that treatment with a cocktail of **85 (MG-101**, Mpro inhibitor) and **82 (Sitagliptin**, PLpro inhibitor) exhibited significant inhibitory effects on SARS-CoV-2 Delta variant growth. Also, a combination of **84 (Lycorine HCl)** or **86 (Nelfinavir mesylate)** and **85 (MG-101)** exerted a significant reduction in virus titer. Overall, in addition to providing a rapid screening methodology for the development of SARS-CoV-2 antivirals, the study outcome also indicates that combined inhibition of Mpro and PLpro is a prudent strategy for inhibiting SARS-CoV-2 and the delta variant (Fig. 21) [106].

Bung et al. performed a study for the design (de novo) of small molecules utilizing deep neural network-based generative and predictive models. Specifically, the models were leveraged for the design of 3CL pro inhibitors. As a result of this study, 31 potential NCEs were pinpointed with some of them demonstrating striking resemblance to the HIV protease inhibitors. On the whole, identified compounds appear to be promising agents to inhibit the 3CL protease of SARS-CoV-2 [107]

Franko et al. designed modular, tunable autoproteolytic gene switches (TAGS) based on synthetic autoproteolytic transcription factors that demonstrated protease inhibition-dependent transactivation ability. The designed synthetic transcription factors were employed to determine the effects of mutations on the protease, and report on the compound’s intracellular inhibitory activity. Specifically, the impact of drug candidates on Mpro and PLpro activities was reported by TAGS with a high signal-to-noise response and a sensitivity matching concentration range inhibiting viral replication. It is highly anticipated that TAGS might emerge as efficient tools to expedite the drug development endeavours for futuristic cell- and gene-based therapies [108].

Anti-SARS-Cov-2 scaffold identification via traditional HTS assays is often hurdled by factors such as high costs and low hit rates. To overcome this hurdle, Xu et al. developed and employed machine learning models for the identification of compounds that can inhibit the SARS-CoV-2 entry into human host cells. Encouragingly, the best performing models led to an increase in the assay hit rate by 2.1-fold for viral entry inhibitors and 10.4-fold for 3CLpro inhibitors. As a result, several compounds with potent activity in a SARS-CoV-2 live virus assay were identified. These findings validate the use of machine learning models as an effective approach to expedite the anti-SARS-CoV-2 drug discovery endeavours [109].

Recently, FDA issued EUA to Paxlovid™ on 22 December 2021 for COVID-19 (mild to moderate, patients age ≥ 12). As such, Paxlovid™ is a combination treatment comprising a cocktail of nirmatrelvir and ritonavir (low dose) to slow down hepatic metabolism. Notably, the peptidomimetic 3C-like protease inhibitor, **87 (Nirmatrelvir)**, bears a glutamine-mimicking pyrrolidone moiety (P1 domain) and a cyano group (electrophilic warhead, P1’). Notably, **87 (Nirmatrelvir)** is a reversible covalent inhibitor as it forms a thioimidate adduct with the catalytic cysteine of Mpro and is endowed with in vitro and in vivo efficacies against SARS-CoV-2 [110]. Importantly, Nirmatrelvir (**87**) is suitable for oral administration [111]. The combination was found to reduce the hospitalization risk and mortality in patients with mild COVID-19 (Fig. 21) [112, 113].

Liu et al. recently reported the discovery of GC376-based small molecule SARS-CoV-2 3CLpro inhibitors possessing drug-like properties. Reportedly, **88** (**NK01-63, coronastat**) was found to be the most promising compound endowed with magnificent inhibitory potency towards SARS-CoV-2 3CL pro (IC_50_ = 16 nM). It was also observed that **88** could block the viral replication of human CoVs (alpha OC43 and beta 229E) with EC_50_ values < 100 nM in Huh-7 human cells. In addition, the drug also blocked MERS-CoV replication (EC_50_ values < 1 μM) and SARS-CoV (EC50 values < 3 μM) in Vero 76 cells. Further investigation results disclosed that **88 (NK01-63)** did not exhibit in vivo toxicity when administered via IP or oral dose. Moreover, a substantial concentration of **88 (coronostat)** could be delivered to plasma and lungs via both routes. Collectively, **88 (Coronastat)** was deduced to be a potent small molecule protease inhibitor demonstrating the potential for SARS-CoV-2 infection treatment (Fig. 21) [114].

## RdRp inhibitors

RdRp (nsp12) represents a prudent and well-explored target for the design of antiviral inhibitors. As such, RdRp catalyzes the viral genome synthesis, which serves an imperative role in coronavirus replication. Both nucleosides, as well as non-nucleoside-based drugs have been explored as RdRp inhibitors, however, the development of non-nucleoside-based inhibitors has often been hurdled owing to the issue of drug resistance [115].

### Remdesivir

**Fig. 22:**
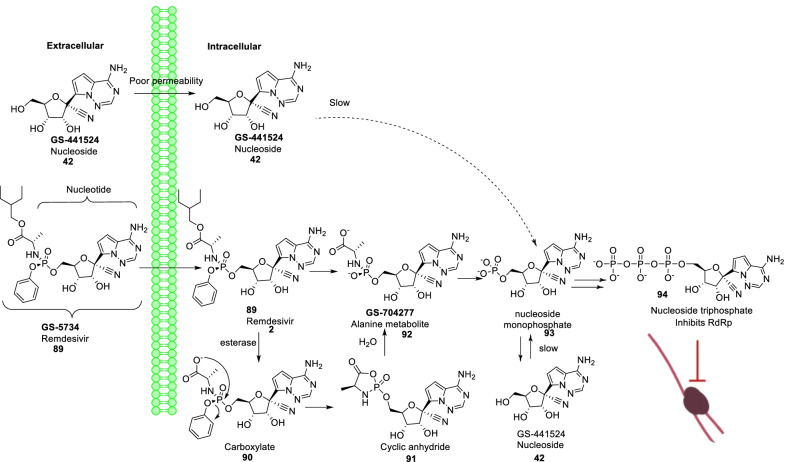
Intracellular conversion of Remdesivir

Remdesivir (**89**) is a broad-spectrum antiviral medication developed by Gilead Sciences. Though originally developed for the Ebola treatment, it demonstrated the ability to effectively block SARS-CoV-2 infection (EC_50_ = 0.77 μM) [115–123]. Overall, **89 (remdesivir)** forms a complex with the SARS-CoV-2 polymerase-RNA complex and indicates a mechanism of delayed-chain-termination [120, 121]. Williamson et al. explained the potency of **89 (remdesivir)** for SARS-CoV-2 in animal models by hampering translocation in the RdRp synthesis due to a steric interaction with serine [122, 123]. Pleasingly, **89 (remdesivir)** was found to be endowed with low cytotoxicity (CC_50_ > 100 μM). Chemically, **89 (remdesivir)** is an aryloxy phosphoramidate prodrug that gets converted to remdesivir nucleoside monophosphate (**93**) via a complex series of reactions. Remdesivir nucleoside monophosphate (**93**) is then efficiently converted to nucleoside triphosphate (**94**) that targets the RNA virus polymerase and prevents viral replication (Fig. 22) [115, 116]. Literature precedents indicate that a 1’-cyano-substituted adenine C nucleoside (**42, GS-441524**) is poorly phosphorylated and its aryloxy phosphoramidate trimester prodrug, **89 (remdesivir)**, can bypass the perceived slow first phosphorylation (Fig. 22). Despite of the fact that **94** (nucleoside triphosphate) is an excellent inhibitor of the viral RdRp, the antiviral activity of **89 (remdesivir)** is highly variable in different cell types. This variable activity profile is attributed to the inconsistent expression pattern of enzymes required for conversion **89** to **93** (Fig. 22) [117–119].

Wit et al. in 2020 employed the rhesus macaque model of MERS-CoV infection and reported the potential of **89 (remdesivir)** (administered before or after the animals were infected with MERS-CoV) to reduce disease severity, virus replication, and damage to the lungs [124]. Remdesivir (**89**) administered intravenously in adults hospitalized with COVID-19 (double-blind, randomized, placebo-controlled trial, ACTT-1, NCT04280705) demonstrated better results than placebo in terms of shortening the time to recovery and reducing lower respiratory tract infection in adults hospitalized with COVID-19 [125]. Results of a clinical trial (solidarity trial) involving 11,266 patients from 405 hospitals in 30 countries revealed that there was almost no difference in mortality (critically ill patients in the remdesivir group and control group). Based on the aforementioned results, it was concluded that **89 (remdesivir)** exerted little or little or no effect on the mortality of hospitalized patients [126]. Despite the failure of the solidarity trial in the context of attaining favourable trends in the mortality rate, the trial results did not nullify the efficacy of **89 (remdesivir),** as shortened recovery time assessed by the ACTT-1 was considered to be a better criterion for measuring the effectiveness of **89 (remdesivir)** [125–127]. The US FDA on 22nd October, 2020 officially approved **89 (remdesivir)** as the first and only COVID-19 treatment drug. Overall, it was ascertained that **89 (remdesivir)** is only effective in terms of improving the percentage recovered (hospitalized adult patients with COVID-19) along with a small reduction in the proportion receiving ventilation but not in terms of reduction in mortality rate [127].

Several studies advocate for the superiority of remdesivir’s parent nucleoside **42** (**GS-441524)** in the context of COVID-19 treatment. The superiority is attributed to i) simpler structure and ease of synthesis of **42** (**GS-441524)** in comparison to remdesivir ii) identification of **42** as a major metabolite of remdesivir in circulation iii) evidenced antiviral activity profile of **42 (GS-441524**) against various strains of coronaviruses. Despite the aforementioned optimistic revelations, the candidature of **42 (GS-441524)** is often debated as primary HAE cells infected with SARS-CoV-2 demonstrate higher sensitivity to **89 (remdesivir)** [128, 246]. Intrigued by this, Li et al. performed an in-depth investigation of the in vitro and in vivo anti-SARS-CoV-2 activities of **42** (**GS-441524)** and reported that **42** (**GS-441524)** significantly inhibited SARSCoV-2 in three cell lines (Vero E6, Calu-3, and Caco-2). It was observed that, in a lethal MHV-A59 mouse model, **42** (**GS-441524)** could reduce the viral loads in the liver and prevent death. Moreover, in AAV-hACE2 mice infected with SARS-CoV-2, **42 (GS-441524)** caused substantial reductions in viral titers. Delightfully, **42** (**GS441524)** also demonstrated the potential to inhibit SARS-CoV-2 replication in the lungs and alleviate lung inflammation and injury. Collectively, the study results underscore the potential of **42 (GS-441524)** for the treatment of COVID-19 [129].

A substantial amount of research is being conducted at present in pursuit of finding suitable alternatives to **89 (remdesivir)** for COVID-19 treatment and either via the design of new agents or agents furnished through structure alteration of **89 (remdesivir).** As such, the chemical architecture of **89 (remdesivir)** comprises an appropriately positioned primary amine functionality that can be leveraged to fuse the remdesivir scaffold with other potent antiviral agents to extract synergistic effects.

Recent revelations indicate that the chaperone protein, HSP90, is a widely implicated target in viral disease. Indeed, a study conducted on resorcinol ring bearing HSP90 inhibitor, ADX-1612, reported that the drug is endowed with remarkable potency at nanomolar concentration as evident from the results of in vitro studies. Much to the delight, the effects of **ADX-1612** were observed to be similar or more pronounced than that of **89 (remdesivir)**. The mechanistic studies revealed that **ADX-1612** exerts inhibition of proteins that are associated with viral replication and infection. The mechanism of action of **ADX-1612** is complementary to the mechanism of action of **89 (remdesivir)** in the context of nucleic acid inhibition. These insights underscore the fact that **ADX-1612**, when used in combination, may increase the activity of other antiviral drugs for COVID-19 treatment. Importantly, HSP90 was also ascertained to be a key pharmaceutical target for viral inhibition as per the expression profiling of SARS-CoV-2 infected human cell lines [130, 131]. Overall, these findings indicate that initiating a clinical evaluation study of a combination of **89 (remdesivir)** with HSP90 inhibitors or development of hybrid scaffolds (remdesivir-HSP90 inhibitors) is the need of the hour.

It is highly anticipated that endeavours centred on RdRp inhibitors based antiviral design will be steered at an expedited pace as the binding modes of nucleoside analogs, **89 (remdesivir)** and **95 (favipiravir)** have been well established based on the study by Rao et al. [132]. Noteworthy to mention that a cryo-electron microscopy structure of full length RdRp in complex with nsp7 and nsp8 (2.9 A°) was solved in the study [108]. Also, the preliminary success of suramin in the context of inhibiting the SARS-CoV-2 infection via targeting RdRp can spur researchers toward non-nucleoside-based inhibitors. Notably, suramin demonstrated a more pronounced activity than remdesivir in inhibiting SARS-CoV-2 infection [133].

### Favipiravir

**Fig. 23:**
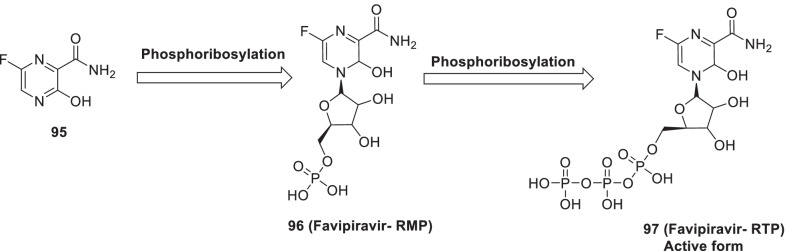
Conversion of Favipiravir to Favipiravir -RTP

Developed by Toyama chemical company, **95 (favipiravir, Avignan)** is a pyrazine carboxamide based derivative. As such, **95 (favipiravir)** is a prodrug that gets converted to **96 (favipiravir-ribofuranosyl-5′-monophosphate)** through phosphoribosylation which further gets metabolized into its **97 (favipiravir-ribofuranosyl-5′ -triphosphate, active form)** to exert its RdRp inhibitory activity (Fig. 23). It was found that **95 (favipiravir)** was endowed with anti-SARS-CoV-2 activity in Vero cells [134–136]. **Favirapir (95)** was approved by China’s state drug administration in March 2020, as China’s first anti- COVID-19 drug. Clinical study results conducted to assess the efficacy of **95 (favipiravir)** in COVID-19 patients (35 patients) (ChiCTR2000029600), demonstrated shorter viral clearance in the favipiravir arm in comparison with the control arm [137]. The drug also exhibited the potential to improve the recovery of COVID-19 patients on the 7th day in a multicentric randomized study. (ChiCTR200030254) [138]. A recent study revealed that **95 (favipiravir)** does not exert any significant beneficial effect in the context of mortality in patients with mild to moderate COVID-19. Moreover, **95** (**favipiravir**) was found to be effective for treating SARS-CoV-2 infection in the clinical trial due to its role in decreasing the lag to the negative conversion of viral RNA which can be detected [139].

### Molnupiravir

**Fig. 24:**
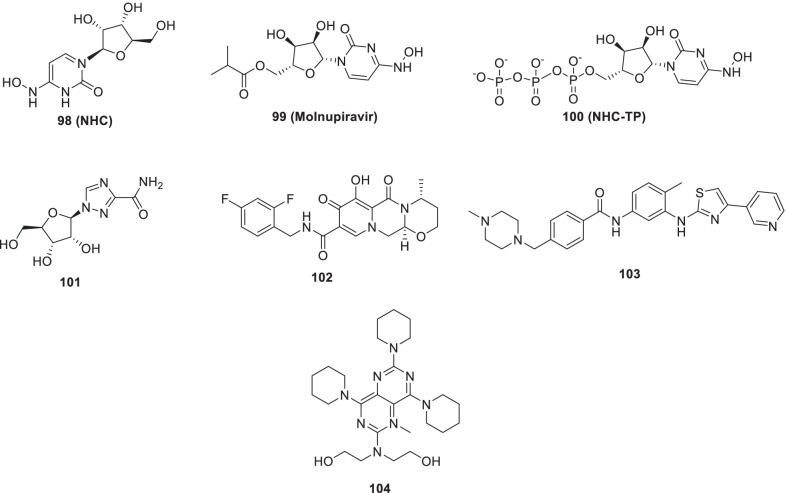
Replication inhibitors (NHC, Molnupiravir, NHV-TP, ribavirin, Dolutegravir, masitinib, suramin)

β-D-N4-hydroxycytidine (**98, EIDD-1931**) is an orally available ribonucleoside analogue that was found to be endowed with antiviral activity against SARS-CoV-2 in Vero cells with an IC_50_ of 0.3 mmol/L. Molnupiravir (**99, EIDD-2801**) is an orally bioavailable prodrug of **98 (EIDD-1931)** that on administration, as prophylactic as well as therapeutic, manifested the potential to improve pulmonary function and reduce virus titer and body weight loss in SARS-CoV infected mice. The mechanism of action of **99** (**EIDD-2801’s**) against the coronaviruses is yet to be fully elucidated, however, the drug demonstrated its antiviral function against influenza viruses via inhibition of RdRp. Notably, **99** (**EIDD-2801**) is effective against remdesivir-resistant mutants and demonstrates a higher genetic barrier to drug resistance than **89 (remdesivir)** [140]. Recent studies conducted to elucidate the mechanism of **99 (molnupiravir)** revealed that the active form of **99** (**EIDD-2801), 100 (β-d-*N*4-hydroxycytidine triphosphate, NHC-TP)** is used by RdRp as a substrate instead of cytidine triphosphate or uridine triphosphate, leading to mutated RNA products. The analysis of RdRp–RNA complexes containing the mutagenesis products indicated the formation of stable base pairs by **98 (NHC)** with either G or A in the RdRp active centre [141]. Thus, it is concluded that an increase of G to A and C to U transition mutations in replicating CoVs leads to significant antiviral effects by **99 (Molnupiravir)** or **98**. Another study to validate the effects of **100 (NHC-TP)** against the purified SARS-CoV-2 RdRp complex confirmed that the mechanism of action of **99** (EIDD-2801’s) is based on RNA mutagenesis mediated via template strand. [142]. Noteworthy to mention that the results of phase 3, a double-blind, placebo-controlled trial of **99** (**molnupiravir**) were optimistic and **99** (**molnupiravir**) administered orally demonstrated efficacy for the treatment of COVID-19. Specifically, the drug led to a reduction in the risk of hospitalization or death in unvaccinated adults (Fig. 24) [143]

Overall, the results of the clinical evaluation of **99 (molnupiravir)** were not very remarkable, improvement in patient recovery was evidenced indicating its potential as useful prophylaxis against SARS-CoV-2 infections. FDA issued a EUA for the use of **99 (molnupiravir)** to treat COVID-19 (mild to moderate). Noteworthy to mention that an oral pill of **99 (molnupiravir)** can be formulated and this is a significant advantage over remdesivir which needs to be administered intravenously (Fig. 24) [144].

## Other replication inhibitors

Ribavirin (**101**), a nucleoside based broad-spectrum antiviral medication exhibits mild anti-SARS-CoV-2 activity (EC_50_ value = 109.5 μM) and a CC_50_ value > 400 μM (Vero E6 cells) [126]. As such, **101 (ribavirin)** is a prodrug that gets converted to ribavarin monophosphate (intracellularly) and further into ribavarin diphosphate and ribavirin triphosphate. Mechanistic studies demonstrate that ribavirin triphosphate inhibits RdRP leading to inhibition of RNA replication (Fig. 24) [133, 145].

The nsp 16, or 2’-O-MTase, catalyzes the 5’-terminal caps structures (m7GpppN) of mRNA for methylation and thus prevents the recognition and activation of host immune responses. In this context, it is considered to be a key protein responsible for SARS-CoV replication. Recently, Dolutegravir (**102**), a 2ʹ-O-MTase inhibitor was found to have inhibitory activity against RdRp as well as Mpro (Fig. 24) [146].

RNA-binding N-terminal domain (N protein) plays a key role in viral RNA transcription and replication. Recently, two compounds (theophylline and pyrimidone derivatives) with a binding affinity towards the N-terminal domain of SARS-CoV-2 N protein were identified [147]. X-ray crystal structure of SARS-CoV-2 N protein (resolution of 2.7 A°) was recently resolved and the revelations were made regarding the specific surface charge distributions required by the drugs to bind to the ribonucleotide binding domain of SARS-CoV-2 N protein [148].

Drayman et al. conducted a screening program of clinically safe drugs for the inhibition of OC43 replication. As such, OC43 is a human beta coronavirus closely related to SARS-CoV-2. The efficacious compounds were pinpointed and further examined for SARS-CoV-2 inhibitory activity. Resultantly, a tyrosine kinase inhibitor, **103 (masitinib),** manifested potent inhibitory activity towards SARS-CoV-2 main protease 3CLpro (IC_50_ = 3.2 µmol/L) (Fig. 24) [149].

Another study’s results also highlighted the potential of **66 (suramin)** to inhibit SARS-CoV-2 replication and protect Vero E6 cells (EC50 ∼20 μM). Moreover, the viral log was also reduced by 2 to 3 logs on treatment with **66 (suramin)**. Further investigation (Time-of-addition and plaque reduction assays) confirmed that **66** prevents the binding or entry of the virus by acting on the early steps of the replication cycle. Inhibition of infection progression was also evidenced on treatment with **66** (**suramin**) in an experiment conducted in a primary human airway epithelial cell culture model [150].

Liu et al. conducted a virtual screening of a U.S. FDA-approved drug library and the efforts invested led to key revelations regarding the SARS-CoV-2 replication suppression ability of **104** (dipyridamole, DIP anticoagulant agent) at a concentration of 100 nmol/L in Vero E6 cells. In this study involving 31 COVID-19 patients, it was observed that after two weeks of DIP adjunctive therapy, significant improvement was observed in 8 of the DIP-treated severely ill patients with clinical cure attained in 7 patients (Fig. 24) [151].

## Inhibitors targeting host protein

Host targeting therapeutic approach is presently being considered as a very potential approach for the reduction of aggressiveness and mortality due to SAR-CoV-2 infections [127].

### ACE2 Inhibitors

Initiation of the molecular events via binding of SARS-CoV-2 viral spike protein S-RBD and ACE2 leads to the intracellular release of the viral genome. In this context, it was envisioned that the adducts endowed with the ability to antagonize the aforementioned interaction can yield therapeutic benefits [152, 153].

**Fig. 25:**
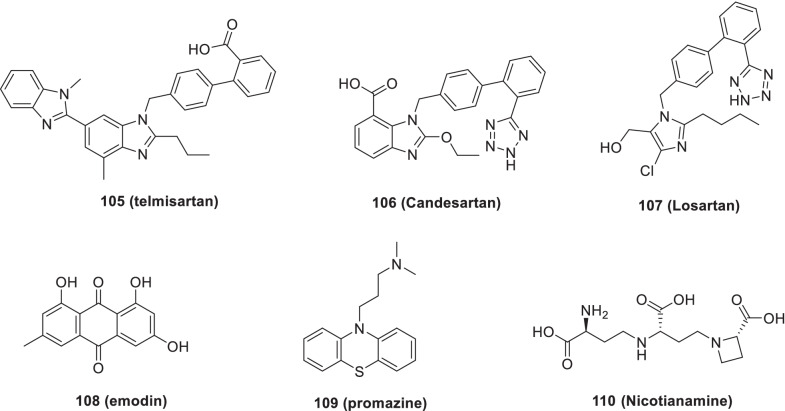
ACE inhibitors as anti-COVID drugs

Recently, the efficacy of **105 (telmisartan)** in reducing C-reactive protein (CRP) plasma levels in hospitalized covid19 patients was investigated in a multicenter superiority trial. The results of the study demonstrated the drug, at a high dose, was capable of exerting anti-inflammatory effects. In addition, the treatment with **105 (telmisartan)** also reduced morbimortality in COVID-19-hospitalized patients [154]. In a study, the impact of **106** (**candesartan**) addition to the standard care regimen in COVID19 patients was evaluated. Resultantly, it was found that the length of the hospital stay of COVID19 patients was significantly reduced to **106 (Candesartan) **(Fig. 25) [155].

In another clinical trial (multicentre blinded randomized), efficacy of **107 (losartan)** was assessed in COVID-19 outpatients. Disappointingly, it was observed that losartan could not reduce the hospitalizations. Also, the treatment with losartan did not reduce the viral load (Fig. 25) [156].

Literature precedents indicate that **108 (emodin)**, an anthraquinone compound, and structurally similar compound **109 (promazine)**, can interfere with the interactions between S protein (SARS-CoV) and ACE2 and exerts inhibitory effects towards the replication. Thus, both the aforementioned drugs are presently being considered prudent options for the treatment of COIVD-19 owing to the similarity between S protein sequences between SARS-CoV-2 and SARS-CoV [157]. Also, **110 (Nicotianamine)**, a potent inhibitor of ACE2 [158] along with flavonoids [159] are also undergoing explorations for ascertaining their potential as cell entry inhibitors that can act by targeting S protein or ACE2 (Fig. 25).

Recently, Sadre Momtaz et al. commenced a scaffold construction program and performed molecular dynamics simulations to pinpoint interaction hotspots on the secondary structure elements of ACE2. Forth, the compendium of discontinuous peptides was generated and evaluated in a bioluminescence-based assay. Resultantly, the peptides could significantly antagonize the SARS-CoV-2 S-RBD: ACE2 interaction, thereby demonstrating the scope of their detailed investigations for the treatment of COVID-19 (Fig. 25) [160].

Working on similar lines, Valeinte et al. designed D-peptide inhibitors to block the association between SARS-CoV-2 spike protein S—RBD and ACE2. Pleasingly, two inhibitors were identified as high- affinity binders of SARS-CoV-2 RBD that displayed significant potential to block the infection of Vero cells by SARS-CoV-2 (IC_50_ values = 5.76 and 6.56 μM). It is noteworthy to mention that both the inhibitors were equipotent in the context of neutralization of the infection of two variants (B.1.1.7 and B.1.351 in vitro) [161].

### TMPRSS2 inhibitors and furin inhibitors

**Fig. 26:**
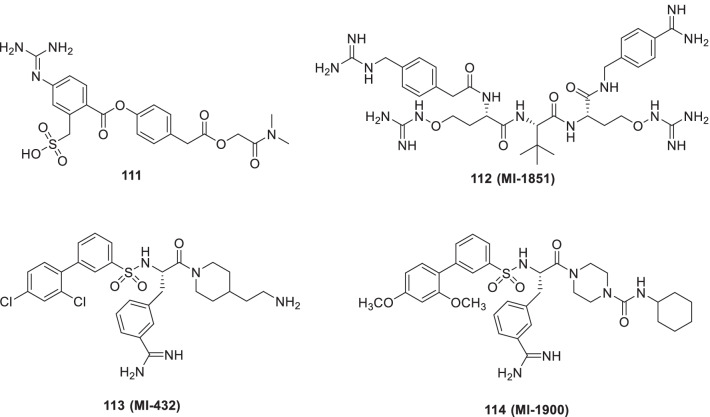
TMPRSS2 inhibitors demonstrating anti-COVID effects

It has been well evidenced that SARS-CoV-2 enters the host cell by binding to ACE2. Notably, the S protein located on the surface of SARS-CoV-2 mediates this interaction. As such, TMPRSS2 cleaves the viral S glycoprotein, thereby facilitating viral activation. In this context, TMPRSS2 appears to be a promising therapeutic target [162, 247]. In pursuit of identifying the cellular factors used by SARS-CoV-2 for entry, Hoffman et al. conducted exploration and concluded that SARS-CoV receptor ACE2 is utilized by SARS-CoV-2 for entry and TMPRSS2 for S protein priming. Validation of the aforementioned was done by observing the potential of **111 (Camostat mesylate)**, a TMPRSS2 inhibitor to significantly reduce SARS-CoV-2 pseudovirus entry into Calu-3 cells (IC_50_ = 1 µmol/L). Moreover, the TMPRSS2 inhibitor was also found to reduce Calu-3 authentic SARSCoV-2 infection in Calu-3 cells and SARS-CoV-2 pseudovirus infection in primary human lung cells (Fig. 26) [162, 247].

Another TMPRSS2 inhibitor, **53 (nafamostat mesylate)**, demonstrated the ability to inhibit SARS-CoV-2 S-mediated entry into host cells. Noteworthy to mention that **53 (nafamostat mesylate)** was found to be endowed with more pronounced effects (15 folds higher) than **111 (camostat mesylate)** as SARS-CoV-2 S-mediated entry inhibitors. In addition, **53 (nafamostat mesylate)** was also found to be superior to **111 (camostat mesylate)** in the context of blocking SARS-CoV-2 infection [163]. Another study results demonstrated the potential of **53 (nafamostat)** to inhibit SARS-CoV-2 S protein-mediated fusion in a cell fusion assay system. Also, in a cell type dependent manner (Calu-3 cells), the drug **53 (nafamostat)** inhibited SARS-CoV-2 infection in vitro (IC_50_ = 10 nmol/L) (Fig. 26) [164].

Bestle et al. (2022) showed that the two different sites on the surface glycoprotein spike (S) of SARS-CoV-2 must be cleaved by host cell proteases for entering the cells and can be utilized as therapeutic drug targets. They described the cleavage of S at S1/S2 and S29 sites by proprotein convertase furin and TMPRSS2 respectively. Also, the S protein in this virus was reported to be activated by TMPRSS2 in Calu-3 (HAE cells) through TMPRSS2 expression knockdown mediated by an antisense mechanism. Synthetic furin inhibitor **112** (MI-1851) was found to inhibit viral replication in human airway cells. The TMPRSS2 inhibitors **113** (M1-432) and **114** (MI-1900) (peptide mimetics) elicited the potential to dose dependently prevent the proliferation and strong CPE of SARS-CoV-2 in CALU-3 cells. Notably, **113** (MI-432), at 10 µM, could only exert a slight effect on the virus replication, however, demonstrated an amplified effect at 50 µM by causing a reduction in virus titers by 75-fold at 24 h p.i. Strong inhibition of SARS-CoV-2 and a significant reduction in viral titers (35–280 folds) were also evidenced in treatment of cells with **114** (MI-1900) in comparison with control cells (Fig. 26). Delightfully, a combination of **112 (furin inhibitor)** with TMPRSS2 inhibitors exhibited striking antiviral activity against SARS-CoV-2 [165]

**Fig. 27:**
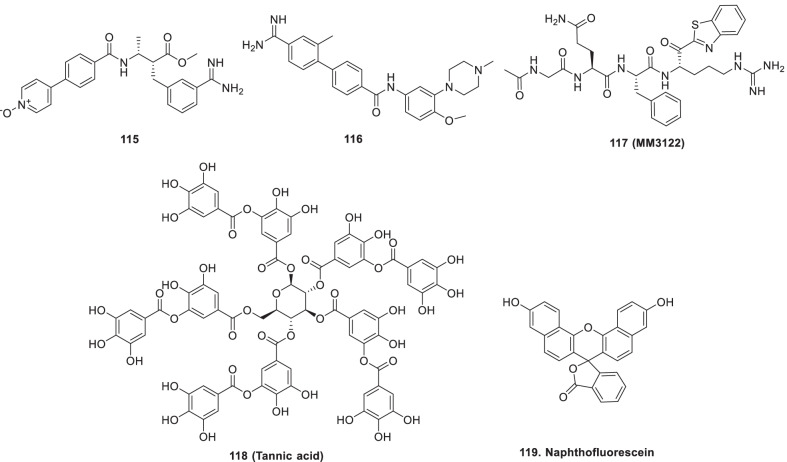
In silico database screening, structure-based drug design, of TMPRSS2 and dual TMPRSS2-Mpro/3CLpro inhibitors

Hu et al. used in silico database screening (pharmacophore-based approach and docking study) of approximately 2 lakh molecules as TMPRSS2 inhibitors and then tested 350 screened compounds in the SARS-CoV-2 pseudotyped particle entry and TMPRSS2 biochemical assay. Resultantly, **115 (Otamixaban) **(Fig. 27) was identified as a potent TMPRSS2 inhibitor (IC_50_ = 0.62 μM). Also, **116** (**NCGC00386945)** (a selective 5-HT1D antagonist) was also found active against TMPRSS2 (IC_50_ = 1.24 μM). Gratifyingly, **116** displayed the potential to act as a viral entry inhibitor and also demonstrated drug-like properties. Structural analysis revealed that the benzoamidinium headgroup was deduced to be an important structural feature responsible for the inhibitory activity of these inhibitors. In addition, NCGC00487181 (COX-2 inhibitor) displayed 70% efficacy on TMPRSS2 (IC_50_ = 3.49 μM) in the enzyme assay. Hence, these molecules can be used as leads for designing the TMPRSS2 targeting drugs for the treatment of COVID-19 (Fig. 27) [166].

Mahoney et al. also demonstrated that in SARS-CoV-2 and other coronaviruses, the spike protein is activated by TMPRSS2 and causes the spreading of the virus in the lung. They found ketobenzothiazoles as substrate specific TMPRSS2 inhibitors using a structure-based drug design with superior activity and potency than **53** (**nafamostat), 89 (remdesivir) and 111 (camostat)**. Amongst all, **117 (MM3122)** blocked the VSV- SARS-CoV-2 chimeric viral entry into Calu-3 human lung epithelial cells (IC_50_ = 340 pM, an EC_50_ of 430 pM), and also inhibited the CPE (EC_50_ = 74 nM). Compound **117** showed an appropriate pharmacokinetic profile (t_1/2_ = 7.5 h and 8.6 h) in lung tissue and plasma respectively. Thus, it is presently being considered a potential molecule for COVID-19 treatment due to its impressive pharmacokinetic profile, metabolic stability and safety profile in mice. Pleasingly, this molecule showed zero toxicity in comparison to **53 (nafamostat)** and **89 (remdesivir)** (toxic at higher concentrations) in Calu-3 cells. In addition to **117**, cyclic peptide VD2173 also acted as a TMPRSS2 inhibitor and demonstrated significant antiviral activity and pharmacokinetic profile (t_1/2_ =  > 8 h in mice) (Fig. 27) [167].

It is well known that TMPRSS2 participates in the pathway involving the entry of this virus into host cells and virus Mpro/3CLpro contributes to its propagation. Wang et al. (2020) in an attempt to capitalize on the aforementioned revelations commenced with a campaign to identify dual TMPRSS2-Mpro/3CLpro inhibitors. To accomplish this, the research group evaluated phytoconstituents present in common fruits to treat SARS-CoV-2 infection. The phytoconstituents selected were kaempferol, catechin, proanthocyanidins, quercetin, tannic acid and resveratrol. A FRET-based assay was used to check their inhibitory activity on the Mpro of SARS- CoV-2. Amongst all molecules, **118 (tannic acid)** was identified as an effective inhibitor of both TMPRSS2 and SARS-CoV-2 Mpro. Compound **118** (Tannic acid) was found to form a stable complex (thermodynamically) with M_pro_ and TMPRSS2 as assessed by molecular analysis (KD = 1.1 μM and 1.77 μM; IC_50_ = 13.4 μM and 2.31 μM) for M^pro^ and TMPRSS2 respectively) (Fig. 27). The suppression of viral entry by **118** (tannic acid) was confirmed by functional assays using the pseudotyped virus particles of SARS-CoV2-S. In light of the aforementioned results, **118 (tannic acid)** appears to be a powerful lead from natural resources to develop new therapeutics for COVID-19 as a potent, safe and effective dual inhibitor of two independent enzymes required for the spread of infection by SARS-CoV-2 (Fig. 27) [168].

Cheng et al. described the role of furin substrate site cleavage in the spike protein of virus for CPE and their production, as this site mediates the formation of syncytium. The furin inhibitors like **119 (naphthofluorescein) (0–20 µM)** and **decanoyl-RVKR-chloromethylketone (CMK, 5 µM)** blocked the SARS-CoV-2 entry and its replication by preventing the formation of the syncytium but **111 (camostat, TMPRSS2 inhibitor)** had no effect through this mechanism. Both the drugs reduced the CPE and production of virus, specifically **CMK** (CC_50 =_ 318.2 µM, IC_50_ = 0.057 µM, SI = 5,567) blocked the entry of virus, suppressed the syncytium and spikes cleavage. Moreover, **119** (**naphthofluorescein**) (CC_50 =_ 57.44 µM, IC_50_ = 9.025 µM, SI = 6.36) suppressed the transcription of viral RNA. For **111 (camostat),** CC_50_ was determined > 2,000 µM by CCK-8 assay, IC_50_ = 0.025 µM by plaque reduction assay and SI > 81,004. Thus, furin inhibitors could be considered potential antiviral agents for this infection (Fig. 27) [169].

### Cathepsin B/L inhibitors

**Fig. 28:**
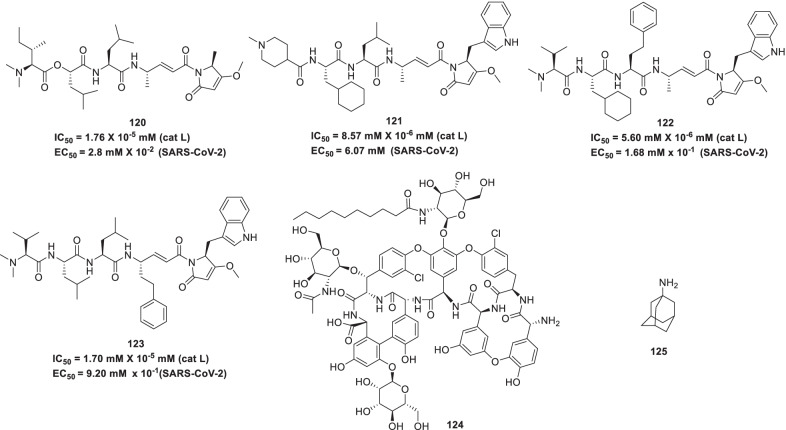
Logical design of Cathepsin B/L inhibitors as anti-COVID scaffolds

In light of the disclosures regarding the candidature of cathepsin L as a COVID-19 drug target, an exhaustive exploration of marine natural product gallinamide A and its synthetic analogues was carried out by Ashhurst et al. Resultantly, some potent inhibitors of cathepsin L (**120–123**) were identified that markedly inhibited SARS-CoV-2 infection. Notably, reduced antiviral activity was observed in TMPRSS2 overexpressing cells. Thus, a cocktail of the inhibitors generated in this study with TMPRSS2 was evaluated and a synergistic improvement in antiviral activity was attained with the combination (Fig. 28) [170].

Zhang et al. assessed the function of cathepsin L in the entry of the virus into the host cell. As **124 (teicoplanin)** binds to cathepsin L, the existence of the cleavage site of cathepsin L on the 2019-nCOV S protein was explored, to advocate the participation of cathepsin L in the entry and fusion of COVID-2019. The authors evaluated the potency of **124 (teicoplanin,** IC_50_ = 1.66 μM) against the infection caused by the 2019-nCoV virus and observed potential inhibition of fusion and entry of 2019-nCoV-Spike-pseudoviruses into the cell. Even though, ex vivo and in vivo studies are pending, the outcomes suggested the efficacy of **124 (teicoplanin)** as an antiviral drug for COVID-19 infection (Fig. 28) [171].

Sacco et al. studied and elucidated the crystal structure of Mpro complexed with three analogs of **14 (GC-376), 38 (calpain inhibitor II**) and **39 (calpain inhibitor XII)** and observed some hydrophobic interactions of these inhibitors (P1 site). These inhibitors were identified to cause dual inhibition of Mpro as well as human cathepsin L, confirming the existence of a hydrophobic methionine side chain in the S1 pocket. Also, **39 (calpain inhibitor XII)** inhibited cathepsin L (IC_50_ = 1.62 ± 0.33 nM) as well as the activation of SARS-CoV-2 spike protein and blocked the entry of the virus. Thus, the aforementioned findings ascertain the potential of the strategy of furnishing dual inhibitors for the host cathepsin L and SARS-CoV-2 M_pro_ to generate potent antiviral drugs for the prevention of viral replication [172].

Smieszek et al. (2020) described the role of proteases [Cathepsin L (CTSL) and Cathepsin B (CTSB)] in the entry of SARS-Cov-2 into the cell via viral spike (S) protein. CTSL/B disruption is being conceived as a prudent strategy and the disruption mechanism includes reduction of CTSL expression, CTSL inhibition and increasing the pH in the lysosomes affecting the environment of CTSL. Owing to their efforts in this direction, the authors identified **125** (amantadine) (10 µM) as a downregulator of the cathepsin expression. Moreover, it was observed that **125** disrupted the lysosomal pathway as evidenced by the results of high throughput drug screen gene expression analysis. Also, **125** was found to inhibit viral replication due to its lysosomotropic effect. Overall, **125** appear to be a worthy candidate capable of decreasing the viral load in SARS-CoV-2 patients (Fig. 28) [173].

### DHODH inhibitors

**Fig. 29:**
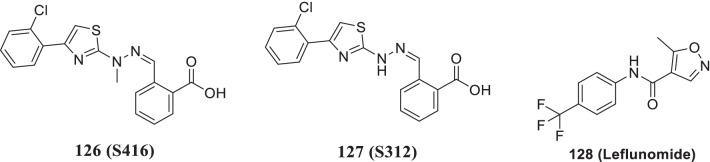
Design of DHODH Inhibitors

Xiong et al. studied the inhibition effect of DHODH enzyme, responsible for catalysing the rate limiting step in de novo synthesis of pyrimidine i.e. dehydrogenation of dihydroorotate to orotic acid to finally produce cytosine and uridine for nucleotide supply in a cell and thus viral replication. The research group identified two potent human DHODH inhibitors, **126 (S416)** and **127 (S312),** with efficacy against SARS-CoV-2 along with desired pharmacokinetic profiles and drug-likeness as compared to Brequinar and Teriflunomide. These inhibitors were also found effective against the Ebola virus, influenza A virus and Zika virus. Notably, compound **126 (S416)** was described as the most potent inhibitor (EC_50_ = 17 nmol/L, SI = 10,505.88) in infected cells. Moreover, **127 (S312)** was reported to possess high potency and low toxicity in infected animals and prevented inflammatory cytokine storm in severe influenza infection in vivo. Thus, these inhibitors by intruding the pyrimidine synthesis pathway inhibited the replication of the virus. Overall, the results validate DHODH as an attractive target to design potent antiviral molecules against the infection caused by SARS-CoV-2 (Fig. 29) [174].

Hu et al. evaluated **128 (Leflunomide)** in ten COVID-19 patients to control the inflammation induced by the immune system as it is an approved DHODH inhibitor used for immunoregulation in autoimmune diseases. The patients selected in the study possessed lung opacity and the condition was confirmed by laboratory tests. **Leflunomide (128)** treatment was given to half of the patients and rest were not given any drugs (blank controls). Standard supportive treatment was given to all the COVID-19 patients selected for the study. The viral shedding time was found to be decreased in the patients treated with **128 (Leflunomide)** (median = 5 days) in comparison to the controls (median = 11 days, P = 0.046). The inflammation due to immunopathological reaction was controlled in the treatment group due to a major decline in C-reactive protein levels. The treatment group was discharged earlier from the hospital than the control group with no reports of side effects. Thus, the use of this drug was suggested as a potential antiviral drug against COVID-19 (Fig. 29) [175].

## Mechanistically diverse inhibitors and small molecule based viral entry inhibitors

**Fig. 30:**
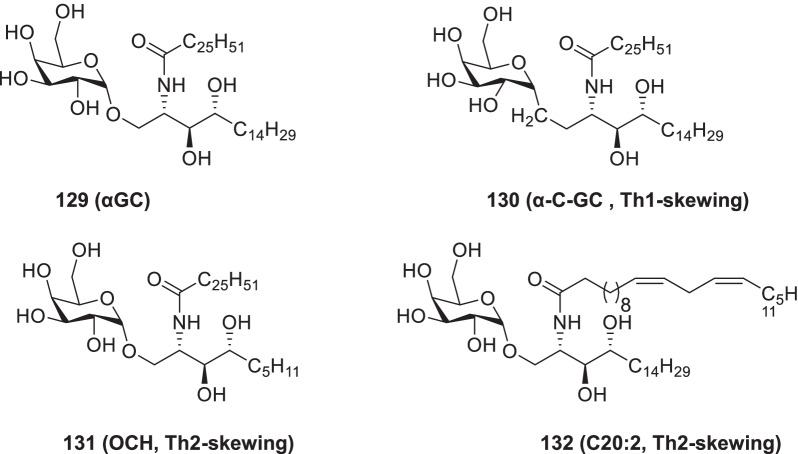
Th1-biasing (α-C-GC) and Th2-biasing (OCH and C20:2) analogues

Rapid production of Th1- and Th2-associated cytokines is caused by α-Galactosylceramide (αGC, a classical iNKT cell agonist). At present, it is being envisioned that the properties of αGC such as the dose-sparing effect and ability to promote a rapid rise in serum IgG after one immunization can be quite useful in COVID-19. In light of these revelations, Wang et al. furnished **129** and **130** [Th1-biasing (α-C-GC)] as well as **131** and **132** [Th2-biasing (OCH and C20:2)] analogues and conducted the immunological study by evaluating the adjuvant activity of αGC in subunit vaccine candidates with different doses of the RBD-Fc protein. Delightfully, it was observed that αGC-adjuvanted RBD-Fc demonstrated a significant humoral response and enabled the sparing of antigens. Also, compound **132 (Th2-biasing agonist C20:2)** exerted a markedly higher titer of the neutralizing antibody than alum. In a nutshell, it is anticipated that the use of αGC-based glycolipids can lead to conclusive benefits in terms of enhancement of the immunogenicity of the RBD-Fc Protein as well as a reduction in the dose of antigen (Fig. 30) [176].

**Fig. 31:**
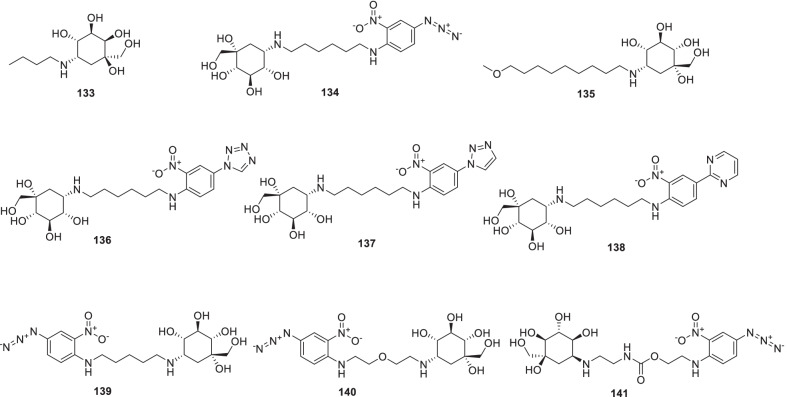
Glycomimetic valiomine derivatives as potent inhibitors of ER α-Glucosidases I and II

Literature precedents indicate that endoplasmic reticulum (ER) α-glucosidases (α- Glu) I and II are attractive targets for developing broad-spectrum antivirals as the enveloped viruses for proper folding of glycoproteins rely on the host cell ER quality control machinery. In the quest to capitalize on the aforementioned disclosures, a series of glycomimetic valiomine derivatives as potent inhibitors of ER α‑Glucosidases I and II were accomplished by Karade et al. The results of the biological evaluation were overwhelmingly positive as some compounds were identified that demonstrated striking inhibitory potential towards α-GluI and α-GluII. The adducts were also evaluated for their potential to reduce the replication of SARS-CoV-2 England/2/2020 in a human lung epithelial cell line (Calu-3). Pleasingly, some of the compounds were found to be endowed with inhibitory activity towards SARS-CoV-2. The representative structures (**133–141)** are shown in Fig. 31 [177].

**Fig. 32:**
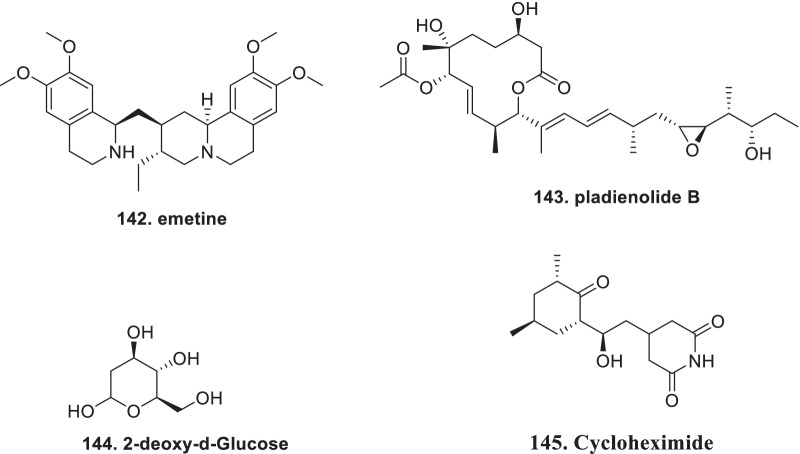
40S ribosomal protein S14 inhibitor, spliceosome inhibitor, inosine monophosphate dehydrogenase inhibitor), hexokinase inhibitor) and translation elongation inhibitor as anti-COVID19 scaffolds

Bojkova et al. developed a model of human cell culture with a SARS-CoV-2 clinical isolate for infection and evaluated the SARS-CoV-2 infection profile by proteome proteomics and translatome3 at regular intervals. The study results reported that small-molecule inhibitors target these pathways and inhibit viral replication such as **142 (emetine**, an inhibitor of the 40S ribosomal protein S14), **143** (**pladienolide B,** a spliceosome inhibitor of SF3B1), **101** (Ribavirin, inosine monophosphate dehydrogenase inhibitor), **144** (2-deoxy-d-Glucose, hexokinase inhibitor), **145 (cycloheximide**, translation elongation inhibitor) (Fig. 32). Overall, the proposed molecular mechanisms of SARS-CoV-2 infected host cells were investigated for identifying better treatment options [178].

**Fig. 33:**
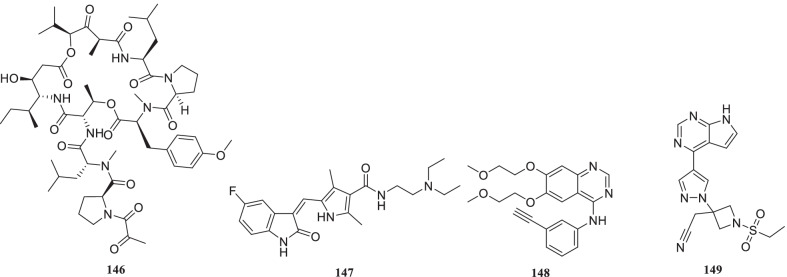
Structure of eEF1A inhibitor and AAK1 inhibitor as anti-COVID19 scaffolds

White et al. reported preclinical efficiency of **146** (**Plitidepsin**) against SARS-CoV-2 through Eukaryotic translation elongation factor 1A **(**eEF1A) inhibition with IC_90_ = 0.88 nM and was found 27.5 times more potent than **89 (remdesivir)**, with reduced toxicity in cell culture. They observed the inhibition of nucleocapsid protein expression by 20 nM (**146, plitidepsin**) after 4 h of infection in the infection cycle of 8 h. These findings suggested the inhibition of replication, expression of N subgenomic RNA (initial stages of infection), and translation of virus by the drug. The research group also determined the drug potency in two SARS-CoV-2 infected mouse models in vivo (IC_90_ = 3.14 nM, SI = 40.4) and observed a decline in replication of the virus in the lungs. Thus, this drug can emerge as an excellent candidate for COVID-19 treatment (Fig. 33) [179].

Richardson et al. proposed the use of adaptor associated kinase 1 (AAK1) inhibitors in 2019-nCoV, as AAK1 regulates the receptor mediated endocytosis of virus entry into host cells. The lung AT2 alveolar epithelial cells are the most affected host cells and the receptor present on the surface of these cells is ACE 2. Screening of 378 AAK1 inhibitors (out of 378, 47 are approved drugs) was carried out and it was observed that 6 inhibited AAK1 with high affinities such as **147 (sunitinib)** and **148 (erlotinib)**. In the study, **149 (baricitinib)** was found to reduce viral infection in lungs due to disruption of AAK1, viral passage into cells and its particles assembly intracellularly. Compound **149** also demonstrated binding affinity to cyclin G-associated kinase (endocytosis regulator) and was found to be endowed with janus kinase inhibitory activity. As AAK1 inhibition was achieved with **149 (baricitinib**, dose = 2/4 mg OD), this group suggested its clinical trial in 2019-nCoV patients with acute respiratory disease. Noteworthy to mention that an early warning model (MuLBSTA score) was utilized to predict mortality in viral pneumonia (Fig. 33) [180].

**Fig. 34:**
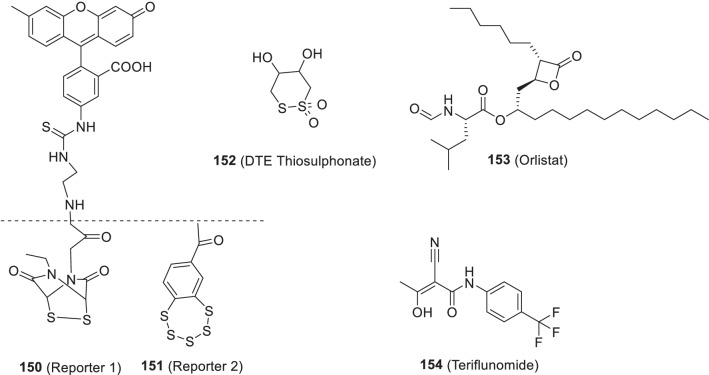
Thiol-reactive antivirals and VPS-34 inhibitors as anti-COVID19 scaffolds

Cheng et al. used the mechanism of swapping thiol-disulfide between substrates disulfides and thiols (exofacial) on cell surfaces to develop strained disulfides as inhibitors of thiol mediated uptake to be used as antiviral agents. Fluorescent labelled cyclic oligochalcogenides (COCs), **150** and **151** were designed as cell-penetrating reporters for the screening of thiol mediated uptake inhibitors due to the entry of COCs into the cells through this uptake. The screening was done employing a fluorescent microscopy image-based high-content HTS assay (fully automated). The inhibitors designed were benzopolysulfanes (BPS), epidithiodiketopiperazines (ETP), heteroaromatic sulfones, gamma-turned peptides bridged through disulfide, cyclic disulfides, thiosulfinates and thiosulfonates. The results of biological evaluation revealed that treatment with these inhibitors caused an approximately 15% decreased uptake of a reporter in cells. The best inhibitors identified were > 5000 times better (at nanomolar concentration) in comparison to Ellman's reagent disclosing this mechanism as an intricate process with multiple targets due to the dependency of the efficiency of inhibitors on the type of transporters. DTE thiosulfonate **152** exhibited IC_50_ = 50 μM, and was found toxic at 500 μM in A549 human lung alveolar basal epithelium cell line in preliminary studies with pseudo-lentivectors that code for a luciferase reporter gene and express the D614G mutant of the spike protein of SARS-CoV-2, in the infected cells. Compounds **150** and **151** (COCs) were observed as the best inhibitors due to their reversibility in covalent exchange. Thus, it was concluded that thiol-reactive antivirals might act through thiol-mediated uptake inhibition by **ETPs** (MIC = 20 µM), **31 (ebselen)** and **152**. In light of these optimistic findings, developing efficient antivirals acting through thiol-mediated uptake inhibition appears to be a logical strategy (Fig. 34) [181].

Silvas et al. (2020) investigated small molecule inhibitors of lipid metabolism and membrane dynamics like PI3 kinase (VPS34 inhibitors), playing a role in endocytosis, autophagy and other processes which can prevent the replication and production of SARS-CoV-2. The drugs assessed in the study were **153 (Orlistat)** and **154 (Triacsin C)**. This study demonstrated the effect of VPS34 inhibitors as autophagy and replication inhibitors of SARS-CoV-2/-CoV and MERS CoV. The results of the evaluation revealed that VPS34 inhibitors, **153 (Orlistat)** and **154 (Triacsin C)** inhibited the growth of virus in Vero E6 and Calu-3 cells, at a post-entry step. VPS34 inhibitors (**153** and **154**) also disrupted the replication centres of this virus and the availability of membrane for viral organelle formation. Thus, the authors suggested lipid metabolism as an important target to treat this infection and proposed detailed investigations for the elucidation of viral replication mechanisms including its inhibition through VPS34 to identify therapeutic targets of fatty acid metabolism in SARS-CoV-2 growth in calu-3 cells and the relative efficacies of these inhibitors in distinct cell types (Fig. 34) [182].

**Fig. 35:**
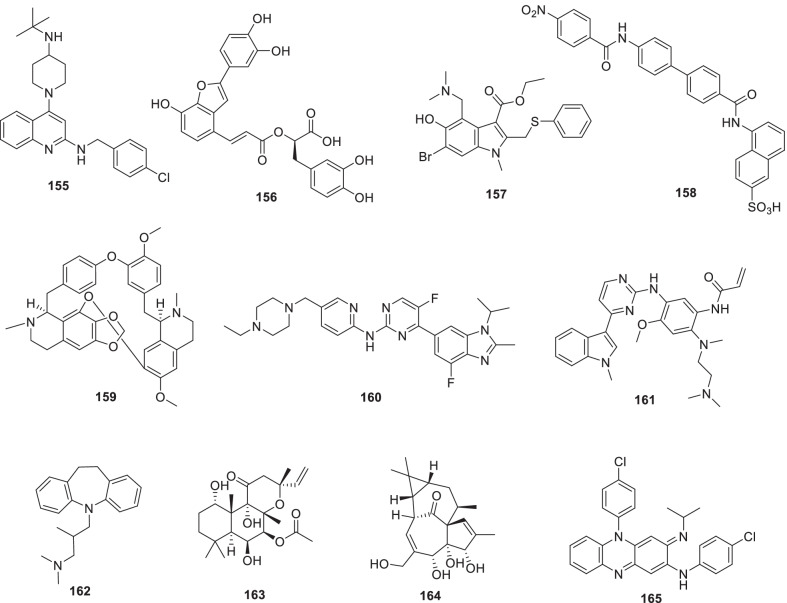
Autophagy inhibitors, S-Protein inhibitors and other chemical architectures as anti-COVID19 scaffolds

In pursuit to develop potent antiviral drugs, Halfon et al. studied and focussed on the mechanism of autophagy inhibition in SARS-CoV-2 to cause the inhibition of viral replication. In the study, **155 (GNS561),** a small basic lipophilic inhibitor of late-stage autophagy that induces dysregulation of lysosomes was explored. It was observed that **155 (GNS561)** exhibited better antiviral effect to treat infections caused by two SARS-CoV-2 strains (IHU-MI3 -MI6, EC_50_ = 0.006, CC_50_ = 2.0 μM and; USA-WA1/2020; EC_50_ = 0.03 μM, CC_50_ = 6.7 μM) than chloroquine and **89** (**remdesivir**, EC_50_ = 0.10 μM and 1.2 μM, CC_50_ = 73.2 μM and > 100 μM) respectively. Using electron microscopy, the potential of **155 (GN561)** to exert autophagy inhibition in SARS-CoV-2 by localisation in LAMP2-positive lysosome and by expanding LC3-II spots size (light chain 3-phosphatidylethanolamine-conjugate) with western blot assay was observed. They also studied the synergism of the different drug combinations and found the combination of **155 (GNS561)** and **89 (remdesivir)** to show strong synergistic efficacy against this virus (in vitro studies*)*. Thus, autophagy inhibition was identified as an alternative route to treat SARS-CoV-2 infection (Fig. 35) [183].

In light of several disclosures, SARS-CoV-2 S protein has been ascertained as an important target for design of drugs for COVID-19 owing to its involvement in recognition of receptor, viral attachment and entry into host cell [184]. Accordingly, several explorations were commenced in this direction that has led to the identification of some promising inhibitors. Recently, **156 (salvianolic acid)** was found to be a potent inhibitor of membrane fusion of S-overexpressed-HEK293T and Vero-E6 cells. Importantly, **156 (salvianolic acid)** demonstrated striking inhibitory effects against SARS-CoV-2 with an EC_50_ value of 3.41 μM [185]. Anti-influenza drug, **157 (arbidol)**, could effectively block or impede the trimerization of SARS-CoV-2 spike glycoprotein (IC_50_ = 4.11 µM). **157 (arbidol)** has also been evaluated at the clinical level as a single agent and in combination with lopinavir/ritonavir [186]. The results of the clinical trials indicated that post-exposure prophylaxis (PEP) using **157 (arbidol)** [187] could reduce infection in individuals exposed to confirmed cases of COVID-19, however, the monotherapy with **157 (arbidol)** could not confer significant benefits to COVID-19 patients (hospitalized) [188, 248]. Another study conducted to design scaffolds capable of inhibiting the hACE2–S protein interaction of SARS-CoV-2 as well as SARS-CoV yielded optimistic results. Several inhibitors were pinpointed through the study that could inhibit the aforementioned interaction with IC_50_ value in the range of 0.2–3.0 μM. Notably, one of the agents (**158**) manifested pronounced SARS-CoV-2-S pseudovirus inhibitory activity with an IC_50_ of 5.6 mmol/L [189]. In the quest to expand the armoury of small molecule inhibitors for the treatment of COVID 19, Chen et al. performed HTS of approved drugs with SARS-S and MERS-S pseudotyped particle entry assays. Resultantly, six broad spectrum spike mediated entry inhibitors (**159—cepharanthine**, **160—abemaciclib**, **161—osimertinib**, **162—trimipramine**, **163—colforsin**, and **164—ingenol)** were identified. A considerable reduction of CPE exerted by SARS-CoV-2 infection in Vero E6 cells was demonstrated by the inhibitors with EC_50_ of 1.41, 3.16, 3.98, 20.52, 23.06 and 0.06 µM, respectively. (Fig. 35) [190].

Yuan et al. reported the efficacy of **165 (clofazimine),** an antileprotic and FDA approved antitubercular drug to treat infection triggered by SARS-CoV-2 (EC_50_ = 0.31 μM, dose = 200 mg d^−1^) and MERS-CoV (EC_50_ = 1.48 ± 0.17 μM) in Vero E6 cells. The drug demonstrated an antagonistic action on replication in lung models (ex vivo) and human primary cell using viral-load reduction assays. It was also found that the drug could cause reduction in viral loads and shedding in lungs and faeces respectively. This drug also prevented the SARS-CoV-2 entry via obstruction of the cell fusion mediated by virus-based spike glycoprotein (S) pseudo-typed virions. Furthermore, where the virus bypassed the cellular entry inhibition mediated by **165 (clofazimine)**, the levels of RNA in the virus were reported to be decreased approximately by 1–1.5 log_10_ in the treated cells. Moreover, **165 (clofazimine)** inhibited the unwinding activity of SARS-CoV-2 helicase (nsp13) utilizing either a double-stranded DNA/RNA substrate. It is assumed that the drug may also revive the transcription process to stimulate the pathways of innate-immunity by causing upregulation of associated genes. During in vivo prophylactic studies, the loss in body weight was found to be reduced with clofazimine (at a dose = 200 mg and C_max_ = 0.41 mg l^−1^) after viral infection, indicating its protective action. Also, after clofazimine administration following virus challenge, the attainment period of pre-infection weight was reduced and the plaque-forming units of virus were decreased by about 1–2 log_10_ in the tissues of lung. In addition, a genome suppression of SARS-CoV-2 was observed in the treated group of hamsters. Moreover, the synergistic action between **165 (low-dose clofazimine)** and **89 (remdesivir)** was evidenced which effectively improved viral control, leading to reduction in loss of body-weight, virus titre in lungs and shedding of virus in nose. It was also observed that decreased dosages of drug were required due to effects on viral replication as prophesied by the Bliss independence model, signifying the synergistic antiviral relationship of above two drugs. Other revelations indicated that 1.25 μM of **165 (clofazimine)** used during an in vitro assay caused an approximately 20-fold reduction of remdesivir concentration necessary for 90% viral replication inhibition and complete absence of cytotoxicity. Thus, the results suggest the use of this drug in the current pandemic and future emergence of COVID-19 diseases (Fig. 35) [191].

**Fig. 36:**
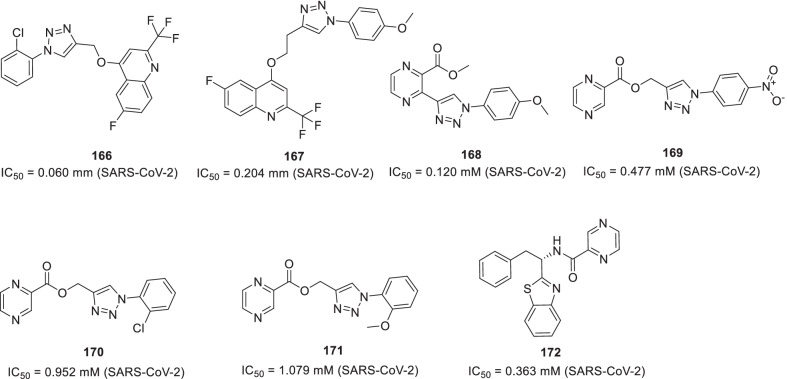
Triazole and pyrazine based Anti-COVID19 scaffolds

Seliem et al. designed and synthesized 1,2,3-triazole tethered conjugates comprising of a quinolone ring via a modified microwave-assisted click chemistry technique. The results of the biological evaluation revealed that two conjugates (**166** and **167**) were significantly potent SARS-CoV-2 [192]. The same research group continued their endeavour to furnish triazole based adducts as potent antiviral drugs for SARS-CoV-2 utilizing the applications of click chemistry. Spurred by the antiviral properties of **95 (favipiravir),** the authors leveraged the pyrazine fragment for the design of the hybrid conjugates. Pleasingly the efforts led to the identification of some pyrazine-triazole conjugates (**168-172**) demonstrating potent activity against SARS-CoV-2 (Fig. 36) [193].

Giovannoni et al. explored the use of immunosuppressants to diminish the immune response in COVID-19 patients with associated multiple sclerosis (MS) like fingolimod (a S1P, sphingosine-1-phosphate modulator in Multiple Sclerosis) to treat ARDS in COVID-19 infection. The disease modifying therapies (DMT) considered in this study were interferon beta Natalizumab, teriflunomide, glatiramer acetate, dimethyl fumarate or ‘lymphodepleting’ DMTs. The authors took into account the potential risk of morbidity and possible mortality for multiple sclerosis patients with SARS-CoV-2 infection and, recommended temporary delay (6 and 12 months) in the initiation/redosing of lymphodepleting DMTs such as rituximab, ocrelizumab, cladribine or alemtuzumab, depending upon activity and disease severity. Also, it is recommended to delay the next dose even beyond 6 months for anti-CD2O DMTs, if lymphocyte counts (CD19 + and CD20 +) are rigorously decreased at the time of the next dose [194].

**Fig. 37:**
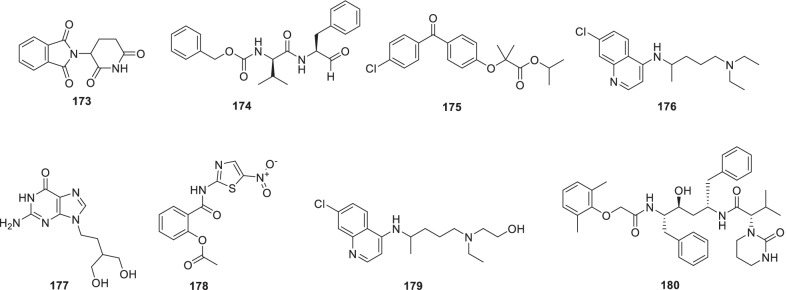
Thalidomide as immunomodulatory agent and structures identified through drug repurposing endeavours

Chen et al. investigated the role of **173** (**thalidomide**) on immune responses in life-threatening organ dysfunction in patients with COVID-19. This drug in combination with low dose short term glucocorticoid was found to inhibit the cytokine surge, reduce pulmonary effusion, relieve digestive symptoms, regulate immune functions, recover the lymphocytes count and decrease O_2_ consumption by inducing calmness in COVID-19 patients. Notably, adverse effects were not observed even with high doses. Thus, the use of thalidomide may be a considered an adjuvant therapy for critical COVID-19 patients (Fig. 37) [195].

## Drug repurposing

Rodon et al.investigated 72 approved drugs in SARS-CoV-2-infection for their ability to inhibit CPE and thus the replication of virus in vitro. It was observed that only a few out of the tested drugs showed IC_50_ < 25 μM such as mesylate hydrate of **63 (nelfinavir), 175 (fenofibrate), 147 (plitidepsin), interferon-γ, interferon 2-α, chloroquine derivatives, 89 (remdesivir)** and **109 (camostat).** Delightfully, **147 (Plitidepsin)** manifested magnificent efficacy at nanomolar concentrations as cathepsin inhibitor leading to prevention of the entry of virus into the cell. Also, cholesterol depleting agents, chloroquine derivatives (IC_50_ below 25 μM) blocked pseudoviral entry into expressing ACE2HEK-293 T cells). It was also observed that cathepsin inhibitors, **174** (**MDL 28,170), NPO-2142, NPO-2143** and **NPO-2260** were endowed with the potential to block endocytosis in ACE2 transfected HEK-293 T and Vero E6 cells in SARS-CoV-2. The research group also evaluated drug combinations to identify synergism or antiviral action at different steps of infection, however, synergism was not observed (Fig. 37) [196].

Wang et al. assessed the potential of **176 (chloroquine), 177** (**penciclovir**), **101** (**ribavirin**), **178** (**nitazoxanide**), **53 (nafamostat), 95 (favipiravir)** and **89 (remdesivir),** against a clinical isolate of 2019-nCoV (BetaCoV/Wuhan/WIV04/20192) in vitro. These drugs were evaluated for their effect on virus yield, infection rates and cytotoxicity in Vero E6 cells through CCK8 assay using DMSO as control. Efficacies were determined using qRT-PCR in terms of quantification of viral copy numbers in the supernatant of cell and confirmed with virus nucleoprotein expression using immunofluorescence microscopy at 48 h post infection. Out of 7 drugs, high concentrations of 3 including **95 (favipiravir)** (EC_50_ = 61.88 μM, CC_50_ > 400 μM, SI > 6.46), **101 (ribavirin**, EC_50_ = 109.50 μM, CC_50_ > 400 μM, SI > 3.65) and **177 (penciclovir)** (EC_50_ = 95.96 μM, CC_50_ > 400 μM, SI > 4.17) were required to decrease the viral infection. Moreover, **53 (Nafamostat)** and **178 (Nitazoxanide)** were found to be endowed with inhibitory potential against the virus with EC_50_ = 22.50 μM, CC_50_ > 100 μM, SI > 4.44 and EC_50_ = 2.12 μM; CC_50_ > 35.53 μM; SI > 16.76 respectively, in Vero E6 cells. Also, chloroquine (EC_50_ = 1.13 μM; CC_50_ > 100 μM, SI > 88.50, EC_90 =_6.90 μM) was found effective against the virus in Vero E6 cells and **89** (**remdesivir**, EC_50_ = 0.77 μM; CC_50_ > 100 μM; SI > 129.87, EC_90 =_1.76 μM) blocked the viral infection both in the Vero E6 and Huh-7 cells. As these drugs were found safe and potential for the treatment of 2019-nCoV infection in vitro*,* further evaluation was recommended (Fig. 37) [197].

Gao et al. based on multi-centwithric clinical trials in China, supported the use of chloroquine phosphate (antimalarial) due to acceptable efficacy in pneumonia associated with COVID-19. This drug was recommended, for treatment, diagnosis, and prevention of pneumonia in COVID-19 patients [198]. Hoffmann et al. reported that SARS-CoV-2 infected Vero cells become chloroquine insensitive due to engineered TMPRSS2 expression and the drug is not able to inhibit this infection in TMPRSS2-expressing human lung cell line Calu-3. Overall, it was deduced that chloroquine targets a viral activation pathway that is not active in lung cells (Fig. 37) [199].

Maisonnasse et al. studied the efficacy of **179 (hydroxychloroquine)** both in vitro and in SARS-CoV-2-infected macaques. Encouragingly, antiviral potency of **179** in Vero E6 (kidney cells of African green monkey) was observed, however, the drug was found ineffective in human airway epithelium model. The evaluation started with the dose of 90 mg kg^−1^, proceeded with 45 mg kg^−1^ (daily maintenance dose) to produce a clinical plasma drug concentration in a group of uninfected NHPs (non-human primates). Subsequently, they also checked a regimen of low dose, (initial dose = 30 mg kg^−1^, maintenance dose = 15 mg kg^−1^). The effect of **179 (hydroxychloroquine)** alone or in combination with azithromycin was evaluated using variable regimens against placebo to check their effect on viral load. Neither of the two drugs displayed any notable effect on viral load in analyzed tissues, either alone or in combination with a high dose regimen of **179 (hydroxychloroquine)** and **azithromycin** (loading dose = 36 mg kg^−1^, daily dose = 18 mg kg^−1^). **179 (hydroxychloroquine)** was not found to be useful for prophylactic and protective treatment against SARS-CoV-2 infection. Thus, the use of **179 (hydroxychloroquine)** was not recommended in monotherapy or with AZTH as a combination, to treat COVID-19 infection in humans (Fig. 37) [200].

A group of experts from the World Health Organization suggested solidarity trials of 4 antiviral drugs (repurposed) namely **180 (lopinavir), 89 (remdesivir), interferon beta-1a and 179 (hydroxychloroquine)** in COVID-19 hospitalized patients. To examine in-hospital mortality, the inpatients were assigned randomly and equally in all treatment groups in comparison to control. While calculating the rate ratios of mortality, age and status regarding mechanical ventilation were considered. 11,330 adult individuals underwent randomization at 405 hospitals in 30 countries; 1411 to **180** (**lopinavir**) (without interferon), 2750 were assigned to receive **89 (remdesivir),** 954 to **179 (hydroxychloroquine**), 2063 to interferon and 4088 to no trial drug. The above drugs were neither found to be effective in reducing the death rate, nor were able to reduce the assistance of ventilation or hospital stay period (Fig. 37) [201].

**Fig. 38:**
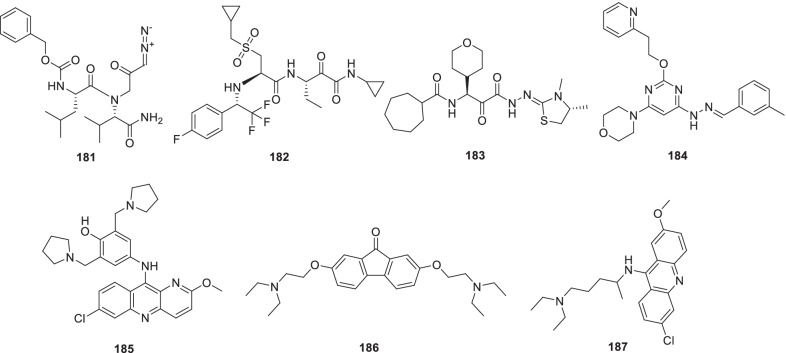
Drug identified through large-scale compound repurposing program

Recently, Riva et al. commenced with a large-scale compound repurposing program (screening of a library of 12,000 small molecules) and identified numerous compounds with potential to inhibit viral replication of SARS-CoV-2, with 21 drugs showing a dose–response relationships. The compounds were also evaluated for their efficacies against SARS-CoV-2 replication supporting human cell lines other than the vero cells. Resultantly, it was observed that thirteen compounds exhibited an EC_50_ < 500 nM in at least one cell line indicating that they can inhibit viral replication with therapeutic administration. Specifically, **174 (MDL-28170), (181) Z LVG CHN2, 182 (VBY-825), 183 (ONO 5334)** and **184, (apilimod, kinase inhibitor)** were found to achieve therapeutic concentrations in patients. The research group assessed the efficiency of these drugs on the inhibition of viral entry, with time-of-addition studies, at different times of study and on infectivity of SAR-CoV-2 spike (S) protein using pseudotyped vesicular stomatitis virus-based virus-like particles, VSV G protein or MERS S protein. These molecules prevented the replication of virus by blocking their entry. The selected protease inhibitors were deprived of inhibitory activity on 3C-like protease of this virus and PLpro suggesting the host protease inhibition as the mechanism of antiviral activity. Moreover, the fusogenic activity of the virus depends on the proteolytic processing of the S protein of the virus via endosome which requires human cysteinyl cathepsins and these cathepsins were used as targets for inhibition by **174 (MDL 28,170, cathepsin B inhibitor), 183 (ONO 5334, cathepsin K inhibitor)** and **182 (VBY-825, reversible cathepsin protease inhibitor).** Moreover, viral replication (primary human lung explant model) was also found to be inhibited by **184 (apilimod), 183 (ONO 5334)** and **174 (MDL-28170)**, in human pneumocyte-like cells derived from induced pluripotent stem cells. The study results are extremely optimistic and accelerated profiling of these drugs appears to be logical for COVID-19 treatment as these drugs are already in public domain and their safety profiles are well established (Fig. 38) [202].

Puhl et al. tested three drugs, **185 (pyronaridine, antimalarial), 186 (tilorone, anti-SARS-CoV-2)** and **187 (quinacrine, antiprotozoal)** in monocytes, Vero76, VeroE6, Calu-3, Caco-2, HUH-7, and A549-ACE2 with SARS-CoV-2, HCoV 229E and MHV infection in vitro. It was observed that the replication of virus was inhibited in A549-ACE2 cells by **185 (pyronaridine, IC**_**50**_** values = 198 nM, respectively)** and **186 (tilorone, IC**_**50**_** values = 180 nM).** The binding capacity of these two drugs to the spike protein was evaluated by employing microscale thermophoresis and K_d_ values of 647 and 339 nM, were observed with **185 (pyronaridine)** and **186 (tilorone)**, respectively. The research group also conducted experiments to elucidate the lysosomotropic mechanism of these compounds. Surprisingly, **187 (Quinacrine)** did not show in vitro activity in Vero76, Calu-3 and Vero E6, but demonstrated activity in MHV (IC_50_ = 2.3 μM), Caco-2 (EC_90_ = 10.54 μM) and A549-ACE2 (IC_50_ = 122 nM) (Fig. 38) [203].

**Fig. 39:**
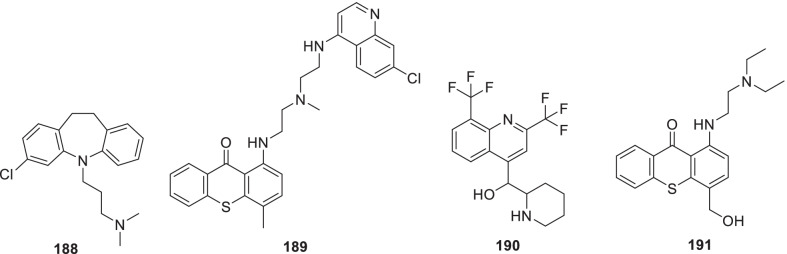
Lysosomotropic compounds as anti-COVID agents

Gorshkov et al. assessed 7 lysosomotropic compounds and found that 6 of these compounds viz **188 (clomipramine), 189 (ROC-325), 177 (Chloroquine), 179 (hydroxychloroquine), 190 (mefloquine)** and **191 (hycanthone)** inhibited the viral CPE in Vero E6 cells (EC_50_ = 2.0 to 13 μM, SI = 1.5- to > tenfold) in comparison to **89 (remdesivir)** (EC_50_ = 7.04 μM) with no apparent cytotoxicity. In the EpiAirway 3D tissue model, treatment with **189 (ROC-325)** led to reduction in viral titres along with blockade of viral entry due to rise in the pH of lysosomes, autophagy. Also, functions of lysosomes were also blocked by these drugs in SARS-CoV-2 infected Vero E6 cells, suggesting a role of the lysosome in the SARS-CoV-2 life cycle. In addition, it was observed that ATP6V0D1 knockdown through siRNA blocked the CPE of HCoV-NL63 in LLC-MK2 cells. Likewise, infected cell lysate showed inhibition to replication of viruses through a maturation blockade of endosomes to the lysosome which is an important step of endocytosis in SARS-Cov-2. Collectively, the outcome of the study confirms lysosomes as an important target in SARS-CoV-2 infections (Fig. 39) [204].

**Fig. 40:**
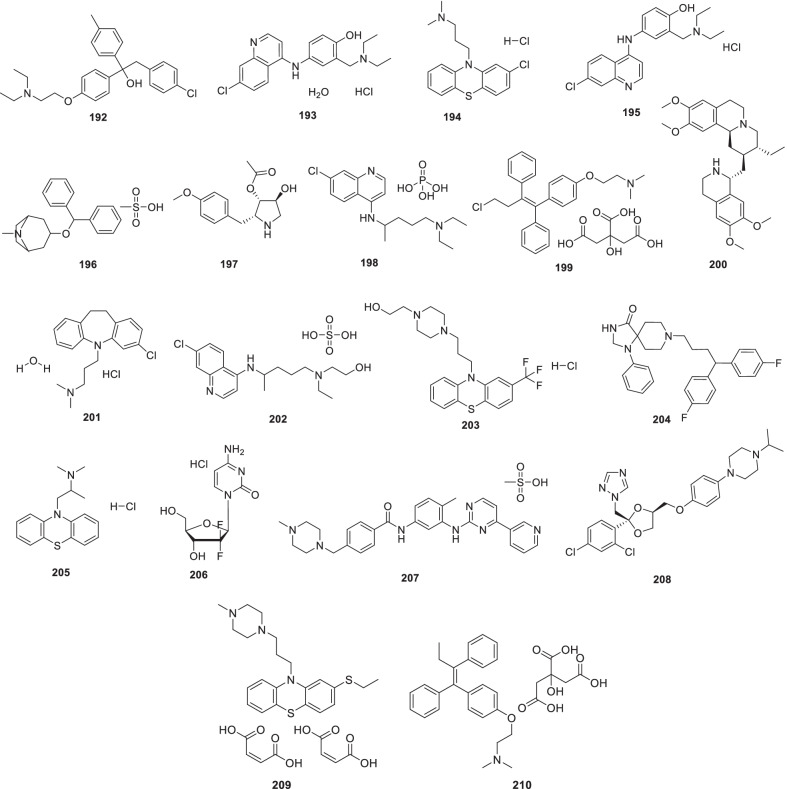
Anti-COVID 19 drugs pinpointed through drug repurposing strategy

Weston et al. (2020) assessed several FDA approved drugs for efficacy against SARS-CoV and MERS-CoV in vitro such as **192 (Triparanol), 193 (amodiaquine dihydrochloride dihydrate), 194 (Chlorpromazine hydrochloride), 195 (Amodiaquine hydrochloride), 196 (Benztropine mesylate), 197 (Anisomycin), 198 (Chloroquine phosphate), 199 (Toremifene citrate), 200 (Emetine dihydrochloride hydrate), 201 (Clomipramine hydrochloride), 202 (Hydroxychloroquine sulfate), 203 (Fluphenazine dihydrochloride), 204 (Fluspirilene) 205 (Promethazine hydrochloride), 206 (Gemcitabine hydrochloride), 207 (Imatinib mesylate), 208 (Terconazole), 190 (Mefloquine hydrochloride), 209 (Thiethylperazine maleate)** and **210 (Tamoxifen citrate)** against SARS-CoV-2 infection*.* Resultantly, seventeen were reported to inhibit SARS-CoV-2 (IC_50_ < 10 µM) and 7 out of 17 inhibited the infectious-SARS-CoV-2 production. In addition, both **194 (chlorpromazine)** and **177 (chloroquine)** were found to inhibit the spike-mediated fusion of SARS-CoV with cell membranes in vitro, protecting against disease without affecting replication of SARS-CoV (MA15) in lungs of mouse but inhibited the coronavirus replication in vitro. The process of endocytosis for entry of virus into the host cell requires clathrin and it was also found to be inhibited by **194 (chlorpromazine)**. With the use of TCID50 assay, all of these drugs were evaluated for inhibitory action on the production of viral particles at non-cytotoxic levels. Seven drugs (chloroquine, hydroxychloroquine, imatinib, amodiaquine dihydrochloride dehydrate, chlorpromazine, mefloquine or amodiaquine hydrochloride), were found to be capable of reducing SARS-CoV-2 replication in A549 cells, reducing the production of RNA, RdRp and thus, virus in vitro (Fig. 40) [205].

**Fig. 41:**
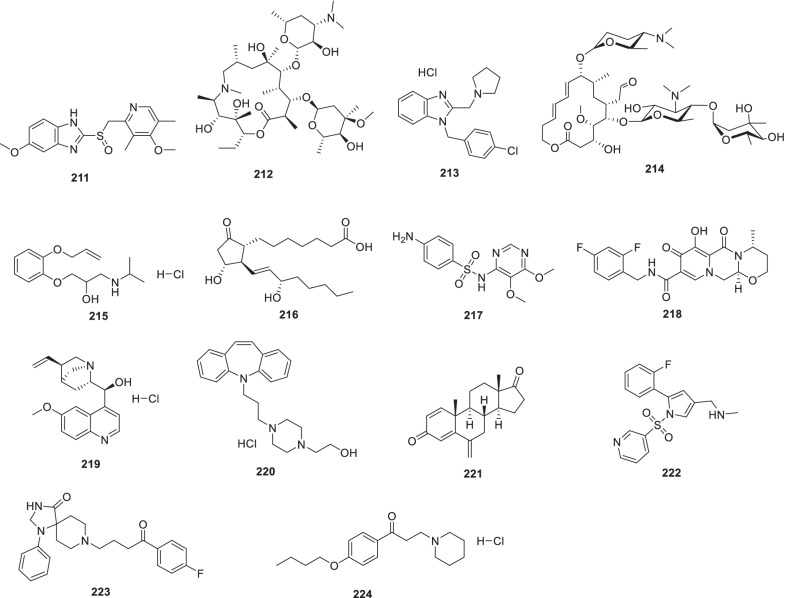
Large scale screening leading to the identification of anti-COVID19 Scaffolds

Touret et al. investigated 1520 approved drugs from PRESTWICK CHEMICAL LIBRARY in a cell‐based assay using Vero E6 and caco-2 cell lines infected with SARS-CoV-2 strain BavPat1 to measure the cell viability 3 days after infection. They showed that 10 μM of **157 (arbidol)** inhibits the SARS-CoV-2 infection causing 70–90% cell viability, with EC_50_ of 10.7 μM and was used as plate-specific reference in calculation of the inhibition index. At 10 μM concentration, they determined the cell viability and calculated the inhibition potency of 1520 drugs in comparison to **157 (arbidol)**. The hits (90 molecules) showed equal or higher inhibition than the reference and were divided into 12 groups depending upon structural similarity and/or therapeutic class with an inhibition index greater than 1. Out of these groups, 15 molecules were selected from different groups with selection criteria of EC_50_ and CC_50_ at 10 μM based on the quantification of the viral genome by Real-Time RT-PCR. Specifically, **211 (Omeprazole), 212 (Azithromycin), 213 (Clemizole hydrochloride), 214 (Spiramycin), 179 (Hydroxy-chloroquine), 215 (Oxprenolol hydrochloride), 216 (Alprostadil), 217 (Sulfadoxine), 218 (Dolutegravir), 219 (Quinidine hydrochloride), 220 (Opipramol dihydrochloride), 221 (Exemestane), 222 (Vonoprazan), 223 (Spiperone), 89 (Remdesivir), 157 (Arbidol)** and **224 (Dyclonine hydrochloride)** showed inhibitory activity towards SARS-CoV-2 in vitro replication. Thus, this study postulated the antiviral potency of the screened drugs to be considered in future for further studies (Fig. 41) [206].

**Fig. 42:**
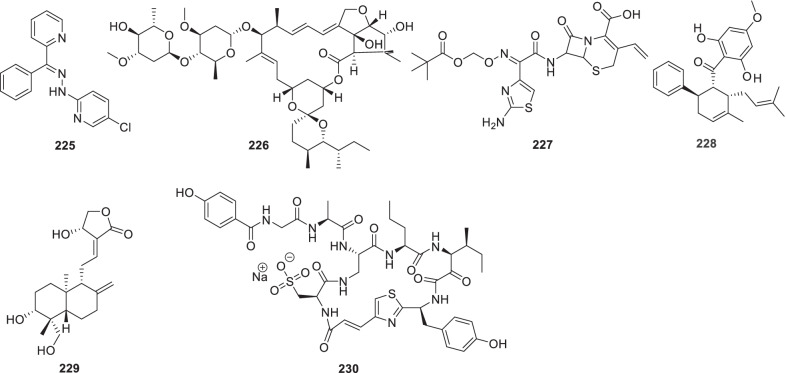
Repurposing approach yielding anti-COVID19 adducts

Son et al. assessed 200 compounds for their inhibitory potential towards SARS-CoV viral replication (Vero E6 cells). The study employed an imaging-based approach and identified **178 (nitazoxanide,** EC_50_ = 4.90 μM)) and **225 (JIB-04**, EC_50_ = 0.69 μM) as potent inhibitors of the viral replication in Vero E6 cells with SI > 150 and, no cytotoxicity at 300 μM. Both the drugs reduced RNA levels of virus intracellularly and were found active against some RNA and DNA viruses (e.g. porcine transmissible gastroenteritis virus) in vitro. Notably, the production of S viral protein was inhibited by **225 (JIB-04)** through suppression of S mRNA transcription. Also, **225** (JIB-04, 0.1–3 μM) exhibited synergistic efficacy with **178 (nitazoxanide)** and **177 (chloroquine, 1–10 μM)** in MA104 cells. Delightfully, **225** (**JIB-04**) also reduced the viral infection in an in vivo porcine model (Fig. 42) [207].

Caly et al. demonstrated the use of an FDA-approved anti-parasitic drug, **226 (Ivermectin,** 5 µM) in SARS-CoV-2 infected Vero-hSLAM cells 2 h post infection. Interestingly, the drug was found to reduce ~ 5000-fold viral RNA after 48 h in vitro (Phase III clinical trial). They proposed inhibition of nuclear import of viral cargo proteins mediated by IMPα/β1 in the cytoplasm and its translocation into the nucleus through the nuclear pore complex where the complex breaks down and the antiviral response of the host cells got reduced by viral cargo, causing enhanced infection. Notably, **226 (Ivermectin)** destabilised the Impα/β1 heterodimer on binding, prevented the viral protein attachment and hence, entrance into the nucleus causing decreased inhibition of the antiviral responses and a more significant antiviral effect **(IC**_**50**_** = 2 μM)**. Overall, **226** (**Ivermectin**) holds enough promise for the treatment of COVID-19 (Fig. 42) [208].

Zhou et al. employed a repurposing methodology to screen antiviral drugs using a pharmacology-based network medicine platform based on HCoV–host interactome and drug targets in the human protein–protein interaction network. 79.7% sequence similarity of nucleotides in 15 HCoV whole genomes was revealed between 2019-nCoV/SARS-CoV-2 and SARS-CoV during the phylogenetic analyses. 96% and 89.6%, sequence similarity was observed between two evolutionarily conserved regions including the envelope and nucleocapsid proteins of 2019-nCoV/SARS-CoV-2 and SARS-CoV respectively. They found 16 potent drugs through repurposing using network proximity analyses of drug targets and HCoV–host interactions in the human interactome (e.g., sirolimus, melatonin, and mercaptopurine). These drugs were further authenticated by enrichment analyses of drug-gene signatures and transcriptomics data induced by HCoV in human cell lines. Thus, this study suggested effective network-based methodologies for quick recognition of potential drugs for 2019-nCoV (Fig. 42) [209].

Huet et al. conducted an Ana-COVID study on March 18, 2020. COVID-19 patients (18 years or older) were selected and admitted to the hospital with severe bilateral pneumonia and typical lung infiltrates confirmed by laboratory tests (lung CT scan). Compound **227 (Anakinra),** a recombinant IL-1 receptor antagonist, was given to the 52 patients in the treatment group subcutaneously (100 mg BOD for 72 h followed by 100 mg OD for 7 days) along with the standard treatments at the institution. In the historical group, patients received only standard treatments and supportive care. In the treatment group, 25% of patients either admitted for invasive mechanical ventilation in the ICU or death prevailed and 13% of patients of this group were observed with increased liver aminotransferases in comparison to the historical group with 9% patients. Overall, it was concluded that **227 (Anakinra)** can be used as an effective drug for the treatment of hyperinflammation in COVID-19 due to less requirement for invasive mechanical ventilation in the ICU and decreased mortality among critical COVID-19 patients (Fig. 42) [210].

Brown et al. (2020) studied the effect of **212 (azithromycin)** and **179 (hydroxychloroquine)** (off-label) in academic and non-academic hospitals in an active comparator, open-label, pragmatic trial design for 300 COVID-19 patients in Utah. For interim monitoring, they used a hybrid Bayesian-frequentist design for contextual assessment and interpretation of the range of possible results from this trial, they developed an inference grid within the context of parallel trials. They started a pragmatic trial to provide treatment options with safety monitoring and informed consent in a structured environment in COVID-19 (Fig. 42) [211].

Kanjanasirirat et al. employed a fluorescence‐based tool for the detection of nucleoprotein in Vero E6 cells along with a plaque reduction assay for the screening of natural product extracts and their phytoconstituents as potential inhibitors of SARS‐CoV‐2. The authors selected one hundred and twenty-two natural products of Thai origin and found the maximum potency in *Boesenbergia rotunda* extract and **228 (panduratin A)** isolated from it, against SARS‐CoV‐2. Also, *Boesenbergia rotunda* extract and **228** were found to suppress the infectivity after viral infection (IC_50_ = 3.62 μg/ mL and 0.81 μΜ and; CC_50_ = 28.06 μg/mL and CC_50_ = 14.71 μM, respectively) in Vero E6 cells. Pleasingly, **228** (**Panduratin A**) showed inhibitory effects both before and after the viral infection in Vero E6 and human airway epithelial cells. Panduratin A and *B. rotunda* extract showed IC_50_ = 5.30 μM, and 20.42 μg/mL; CC_50_ = 43.47 μM and > 100 μg/mL respectively at the pre-entry phase. Similarly, **229 (andrographolide)** and *Andrographis paniculata* extract, also exhibited antiviral potential (IC_50_ = 6.58 μM and 68.06 μg/mL; CC_50_ = 27.77 μM and > 100 μg/mL respectively). Likewise, extract prepared from *Zingiber officinale* also exerted antiviral effects (IC_50_ = 29.19 μg/mL and > 100 μM, CC_50_ = 52.75 μg/mL). Overall, **228 (panduratin A)** and **229 (Andrographolide)** were found to be a potent phytoconstituent for the treatment of COVID-19 (Fig. 42) [212].

In light of the revelations ascertaining elastase as a potential target for the prevention of a common COVID-19 complication known as ARDS, Cui et al.attempted the first total synthesis of **230 (Cyclotheonellazole A)** which is a natural macrocyclic peptide-based elastase inhibitor. Further to establish the structure–activity relationship, the author furnished several analogues of **230 (Cyclotheonellazole A)**. Delightfully, the results of the biological evaluation revealed that treatment with **230 (cyclotheonellazole A)** alleviated acute lung injury and caused reductions in lung edema and pathological deterioration in the in vivo ALI mouse model. Moreover, **230 (Cyclotheonellazole A)** also demonstrated cellular safety (Fig. 42) [213].

Zhang et al. (2020) developed an HTS assay based on CPE in Vero-E6 cells to develop SARS-CoV-2 inhibitors targeting complete life cycle of virus. The CCK-8 assay was used to determine cell viability quantitatively after the reduction of CPE by antiviral drugs (neutralizing human antibody CB62, IFN-α, chloroquine and remdesivir) as positive controls for assay validation. Using validated assay, they assessed a library of 1058 phytoconstituents for the identification of potent SARS-CoV-2 inhibitors in cell culture. 30 hits were found to possess > 50% protection from CPE after the preliminary screening. A dose dependent propagation inhibition of virus was observed for all phytoconstituents (EC_50_ = 0.011 to 11.03 μM). The compounds were also evaluated for CC_50_ values (cell cytotoxicity) and 16 compounds exhibited the selective indexes (SI = [CC_50_]/ [EC_50_]) > 10, suggesting their use for this infection. All these results proved the reliability and robustness of the CPE-based HTS assay for evaluation of SARS-CoV-2 inhibitors [214].

## Peptide based entry inhibitors

### Stapled peptides

In the quest to capitalize on the proven utility of the peptide stapling technique for the improvement of proteolytic stability and membrane permeability, Verdine et al. accomplished a series of stapled analogues that can target SARS-CoV‑2 spike protein HR1 leading to the inhibition of the fusion of virus to its cell receptor. Subsequent evaluation results demonstrated the ability of peptides to block the replication of the pseudotyped SARS-CoV-2 virus. Delightfully, two peptides led to the reduction of authentic virus and dose dependently inhibited the spike protein-mediated cell − cell fusion. As expected, higher helical contents along with enhanced proteolytic stability than their counterparts were observed with these stapled peptides [215].

### Synthetic neutralizing peptides

Recently, screening of peptides was done via quartz crystal microbalance measurement screening by Wang et al. to assess their ability to inhibit the viral entry to the host cell. To accomplish this, the research group designed and furnished three peptide sequences. Pleasingly, the peptides demonstrated high affinity to the S1 subunit of spike protein and SARSCoV- 2 pseudovirus. The peptides could exert conformational changes in the S1 protein as evidenced by the results of circular dichroism spectroscopy. Overall, the peptides manifested inhibitory action towards the infection of SARSCoV-2 pseudovirus and the inhibitory effects were observed to be more pronounced when a cocktail of the three peptides was used. Noteworthy to mention that cellular toxicity (A549 cells and 293 T-ACE2 + cells) was not exerted by the peptides which advocates for the safe use of peptides in the biological system. [216]

### EK1 and EK-14C

EK1 is a peptide-based pan coronavirus inhibitor that targets the HR1 domains of HCoVs proteins. Favourable trends have been evidenced with EK1 in the context of inhibiting the SARS-CoV infection. A study was conducted by Xia et al. to evaluate the efficacy of EK1 and the results were quite optimistic as the peptide treatment (pre as well as post challenge with coronaviruses) led to the protection of the treated mice from HCoV-OC43 or MERS-CoV infection. These results ascertained the prophylactic action as well as the therapeutic potential of EK01 [217]. Also, in another study by Xia et al., the peptide manifested dose dependent efficacy against SARS-CoV-2 S protein mediated membrane fusion and PSV infection [218]. In light of these overwhelmingly positive results, the same group of researchers continued their investigation and furnished various lipopeptides from EK1. Resultantly, EK14C was identified that was endowed with striking inhibitory potential against SARS-CoV-2 S protein-mediated membrane fusion and pseudovirus infection (IC_50_ = 1.3 and 15.8 nM). It is noteworthy to mention that EK1C4 demonstrated 241- and 149-folds higher inhibitory potency than EK1 towards SARS-CoV-2 S protein-mediated membrane fusion and pseudovirus infection. Delightfully, EK14C (intranasally applied) EK1C4 magnificently protected the mice against HCoV-OC43 infection. The aforementioned disclosures indicate that EK-14C is a potent fusion inhibitor that can be utilized for the prevention and treatment of SARS-COV2 infection along with other SARS-CoVs [219].

### IPB02—a HR2 sequence-based lipopeptide fusion inhibitor

Zhu et al. reported IPB02, an HR2 sequence-based lipopeptide fusion inhibitor, endowed with striking inhibitory efficacy against SARS-CoV-2 S protein-mediated cell–cell fusion and pseudovirus transduction. In addition, SARS-CoV pseudovirus was also significantly inhibited by IPB02 [220].

### 2019-nCoV-HR2P

Xia et al. reported the fusion inhibitory potential of 2019-nCoV-HR2P employing a 2019-nCoV S mediated cell–cell fusion assay. The peptide was found to be endowed with significant fusion inhibitory potential with an IC_50_ value of 0.18 µM [218].

### [SARSHRC-PEG4]2-chol

Vries et al. reported a lipopeptide fusion inhibitor that could block the viral spike protein mediated membrane fusion between the viral and host cell membranes. Delightfully, complete prevention of SARS-CoV-2 direct-contact transmission was observed with the intranasal administration of the peptide to ferrets [221]

### SBP1

The strategy of disruption of SARS-CoV-2-RBD binding to ACE2 via peptide-based drugs is presently being considered as a potential approach to inhibit the entry of virus into human cells. Spurred by these revelations, Zhang et al. synthesized a 23-mer peptide fragment of the ACE2 PD α1 helix (SBP1). Resultantly, the biotinylated peptide sequence derived from human ACE2 demonstrated substantial selective binding affinity to Sino Biological insect-derived SARS-CoV-2 spike protein RBD [222].

### Mini proteins

In the quest to capitalize on the aforementioned strategy of preventing the entry of the virus into the cells that is initiated on binding of spikes (present on SARS-CoV-2) to host ACE2 receptor, small and stable proteins were designed by Cao et al*.* that can tightly bind the spikes, thereby blocking its binding to ACE. Some of the designed miniproteins demonstrated high binding affinity and prevented the mammalian vero E6 cells from infection by SARS-CoV-2 (IC_50_ = 24 pM to 10 nM). Noteworthy to mention that the small size and high stability of miniproteins allows their direct delivery to the respiratory system [223].

### ATN-161

Recently, Beddingfield et al. reported the efficacy of ATN-161, an integrin binding peptide, for the treatment of SARS-CoV-2 infection. The study results revealed that ATN-161 could inhibit the binding of SARS-CoV-2 spike protein to both α5β1 and α5β1/hACE2, leading to the disruption of SARS-CoV-2 infection *in vitro*. Moreover, an increase in cell viability in presence of SARS-Cov-2 coupled with decreased CPE associated with viral infection was evidenced with the prophylactic treatment of ATN-161 [224].

### SARS-BLOCK™ peptides

Watson et al. designed SARS-BLOCK™ peptides antidote to COVID 19 to extract dual benefits of a vaccine and a therapeutic. Specifically, the designed peptides were furnished to serve as **antidotes to SARS-CoV-2 spike protein-mediated infection of human ACE2-expressing cells.** Delightfully, the peptides were found to be endowed with substantial binding affinity for ACE2 and a neutralizing antibody against the SARS-CoV-2 RBD. Overall, the outcome of the study revealed that SARS-BLOCK™ peptides could exert a significant reduction in infection and can emerge as effective therapeutic modalities to prevent viral association with ACE2 and infection [225].

### Defensin-like peptide P9R

In a study conducted by Zhao et al., defensin-like peptide P9R demonstrated a magnificent antiviral activity profile against pH dependent viruses (SARS-CoV-2, MERS-CoV and SARS-CoV) leading to inhibition of virus-host endosomal acidification. Also, the peptide exhibited protective effects in mice towards the lethal challenge by A (H1N1)pdm09 virus and reduced the probability of causing drug-resistant virus.

For the in-vivo evaluation of P9R, the mice infected with A (H1N1) pdm09 virus were treated at 6 h after infection, followed by two doses to infected mice for the next day. The P9R-treated mice survival rate was notably better than the PBS-treated group. Moreover, on post infection day 2 and day 6, the body weight loss was remarkably less as compared to the PBS-treated mice. Also, it was observed that P9R markedly impeded mouse lung viral replication. Also, in an antiviral resistance study, P9R prevented the appearance of drug-resistant viruses.

In plaque reduction assay, P9R significantly inhibited the viral replication when it was introduced to the cells post SARS-CoV-2 infection (IC_50_ = 0.9 µg/ml). Additionally, P9R depicted promising results when tested against MERS-CoV, A (H1N1) pdm09 virus, A (H7N9) virus and rhinovirus. In addition to the above assay, P9R was analysed in a multiple growth assay where it inhibited viral replication for viruses like SARS-CoV, SARS-CoV-2 and MERS-CoV by 100-fold, while P9R inhibited > 20-fold viral replication for A (H1N1) pdm09 virus, A(H7N9) virus and rhinovirus. From the aforementioned results, it is evident that P9R strongly inhibits the novel coronavirus SARS-CoV-2 along with more encapsulated and free respiratory viruses.

To test the emergence of resistant virus, (H1N1) pdm09 virus was continually transited 40 times to MDCK cell existing with P9R and zanamivir was taken as a control in this assay. After 10 passages of the virus, zanamivir could not inhibit viral replication and viral resistance was detected. Notably, P9R could effectively inhibit viral replication even when the virus was passaged 40 times (P9R 5.0 µg/ml for 10 passages, 50 µg/ml for remaining 30 passages). Delightfully, viral resistance with P9R was not detected.

Overall, the outcome of the study was overwhelmingly positive, particularly in light of the fact that numerous pathogenic viruses are endosomal pH-dependent viruses. It is also anticipated that the aforementioned findings might also spur other researchers to embark on similar endeavours in pursuit of generating antivirals that can prevent virus-host endosomal acidification [226].

### Dual-functional cross-linking peptide 8P9R

Zhao et al. recently identified 8P9R, an antiviral peptide that demonstrated substantial inhibitory activity towards SARS-CoV-2 and SARS-CoV in vivo and suppressed the SARS-CoV-2 replication in hamsters and SARS-CoV in mice. It is noteworthy to mention that the inhibitory activity of the peptide was attributed to its potential to cross-link viruses leading to the blockade of viral entry on cell surface through the TMPRSS2-mediated pathway and inhibition of endosomal acidification. Thus, 8P9R is a dual-functional cross-linking peptide based inhibitor (TMPRSS2-mediated surface and endocytic pathway of SARS-CoV-2) (Additional file 1).

Noteworthy to mention that the eight branched P9R that is 8P9R displayed a more potent antiviral activity with IC_50_ of 0.3 µg/ml than the previously discussed P9R (IC_50_ of 20.2 µg/ml). The cross-linking ability of 8P9R was exhibited by taking TEM images where cross-linked SARS-CoV-2 was observed to make a large viral cluster. This result was further confirmed with fluorescence-labelled H1N1 virus where previously studied P9R and P9RS did not cross-link virus to get a bigger cluster of virus. The outcome was also supported through the observation of confocal pictures. Moreover, the inhibitory effects of 8P9R towards the endocytic pathway of viral infection) was observed to be similar to bafilomycin A1. Also, 8P9R demonstrated the potential to remarkably increase the antiviral activity of **157 (arbidol)**.

For the evaluation of in-vivo efficacy, a 10-month-old mice infected with SARS-CoV was initially treated with 8P9R at 8 h after infection. Pleasingly, toxicity was not observed in mouse lungs as indicated by body weight tracking and H&E staining for the period of 18 days. The other antiviral drugs taken for the study were **157 (arbidol,** 30 mg/kg), **177 (chloroquine**, 40 mg/kg). The outcome of the biological evaluation indicated that the aforementioned drugs as well as their combination did not inhibit SARS-CoV viral replication, whereas 8P9R significantly inhibited SARS-CoV replication in mouse lungs. Noteworthy to mention that **157 (arbidol)** and **177 (chloroquine)** significantly inhibiting SARS-CoV-2 in Vero-E6 cells without TMPRSS2, while in Calu-3 cells, these drugs could not inhibit SARS-CoV-2 which is dependent on TMPRSS2. In contrast, significant inhibition of SARS-COV-2 was observed in both Ver-E6 cells and Calu-3 cells with 89PR. In a nutshell, the approach of simultaneously interfering with the two entry pathways of coronaviruses appears to be a prudent strategy that can yield conclusive benefits [227].

## Conclusion and future perspective

The pandemic created a state of panic across the globe, which in turn spurred the pharmaceutical industrial sector as well as the academia to run parallel programs for the development of vaccines, small molecules, peptides and monoclonal antibodies for the management of COVID19. The current scenario makes it crystal clear that the vaccination drive is at its full pace and the health agencies of all the countries are leaving no stone unturned to vaccinate the majority of the populace. Indeed, measures are being taken to administer the booster dose to the already vaccinated population. Moreover, efforts have also been directed toward the development of improved vaccine platforms. Resultantly, there are a number of vaccines available belonging to different vaccine formats such as inactivated virus-based vaccines, protein-based vaccines, recombinant adenovirus DNA and messenger RNA-based vaccines. Owing to the demonstrated safety and efficacy for the prevention of viral spread, the messenger RNA vaccines are at the forefront in context of priority towards a vaccine platform. Along with the vaccination strategy, the use of masks and social distancing stands as the most effective strategy for the prevention of viral spread.

There is no denying the fact that the vaccines appear to be the best modality for the prevention of SARS-CoV-2 infections, still, the supplementation of the anti-COVID19 arsenal with small molecules that can broaden the range of therapeutic options, particularly for immunocompromised patients, is presently being considered the most pressing need. Astonishingly, the praise-worthy efforts invested towards vaccine development and administration juxtaposed with attempts to create awareness about the beneficial effects of vaccines have not hurdled the commencement of preliminary, preclinical and clinical drug discovery endeavours for small molecule inhibitors and this very fact is quite evident for the literature covered in this review. Inspiringly, the recent approvals of Molnupiravir and Paxlovid™ for the treatment of COVID-19 have instilled a sustained dose of hope and promise in the circulation of the researchers to continue investing efforts in this direction of research. The medicinal chemist has gone the extra mile to generate a voluminous library of anti-COVID-19 scaffolds libraries composed of chemically and mechanistically diverse inhibitors. Notably, among the various well-established targets, the medicinal chemist has biasedly explored 3CLpro and PLpro as targets and employed numerous robust drug design strategies to furnish an armoury of 3CLpro and PLpro inhibitors. It is worth mentioning that such an expedited approach in the context of small molecule generation was unprecedented till now. In the majority of the studies centred on protease inhibitors, researchers leveraged the computational simulation approach to furnish rationally designed adducts. Albeit, the inclination of the chemist was witnessed towards protease inhibitors in the context of preliminary and preclinical explorations, appropriate attention was also paid to the construction of adducts endowed with inhibitory potential towards S protein, RdRp, ACE2, TMPRSS2 and cathepsin. In addition to the new drug design strategies, drug repurposing as a tool for pinpointing new clinical indications from existing drugs was extensively employed during the COVID-19 outbreak. Also, peptide-based entry inhibitors garnered reasonable attention from the researchers and quite of few of the agents belonging to the class of peptides demonstrated substantial promise for the treatment of COVID-19. Peptide-based pan coronavirus inhibitor, Stapled peptides, Synthetic Neutralizing Peptides, HR2 sequence-based lipopeptide fusion inhibitor, an integrin-binding peptide, SARS-BLOCK™ peptides and defensin-like peptide excellently exemplifies some peptide-based viral entry inhibitors. As far as the therapeutic progress of monoclonal antibodies for the treatment of COVID-19 is concerned, they have outscored the small molecule inhibitors in the context of FDA approvals.

Future work in the field of small molecule drug discovery for the treatment of COVID-19 would require the detailed investigation of a plethora of the scaffolds enlisted in this compilation by interdisciplinary teams comprising chemists and biologists. It is highly anticipated that some of the promising anti-COVID19 scaffolds covered in this review will replicate their potential in higher-stage preclinical and clinical investigations. Moreover, the outcome of the studies covered in this compilation in terms of established activity profile, pharmacokinetic profile as well as a structure–activity relationship can be leveraged to further numerous important pursuits in the anti-COVID-19 drug discovery field. However, to sail the ship further, efforts need to be directed towards: (i) profiling of the inhibitors against the emerging variants of SARS-CoV-2 (ii) determination of the therapeutic index of the new small molecules to optimize the safety and efficacy of drug candidates (iii) rapid screening of the libraries of anti-COVID19 scaffold (iv) expansion of the list of therapeutic targets to confer wider scope to the medicinal chemist in context of the generation of new chemical architectures (v) maximizing the use of machine learning predictive models to accomplish the furnishment of anti-COVID19 adducts (vi) construction of antibody–drug conjugates utilizing the promising small molecules enlisted in this review as payloads. In a nutshell, it is highly anticipated that the comprehensive investigation of the armoury of small molecule anti-COVID19 scaffolds will culminate in a therapeutic in the near future.

## Supplementary Information

**Additional file 1: Table S1.** Activity of profile of small molecules as anti-COVID drugs.

## Data Availability

Not applicable.

## Abbreviations

RBD: Receptor binding domain
3CLpro: 3C-like protease (3CLpro)
PLpro: Papain-like protease
NSPs: Non-structural proteins
RdRp: RNA dependent RNA polymerase
SARS-CoV-2: Severe acute respiratory syndrome coronavirus 2
DHODH: Dihydroorotate dehydrogenase
ER: Endoplasmic reticulum
eEF1A: Eukaryotic translation elongation factor 1A
AAK1: Adaptor associated kinase 1
DMT: Disease modifying therapies
ACE2: Angiotensin converting enzyme 2
TMPRSS2: Transmembrane serine protease 2
mAb: Monoclonal antibody
IL-6: Interleukin-6
FDA: Food and drug administration, MERS-CoV- Middle East respiratory syndrome coronavirus
CPE: Cytopathic effect
FRET: Fluorescence Resonance Energy Transfer, HTS, high-throughput screening
CoV: Coronavirus
HAE: Human airway epithelial cell
ARDS: Acute lung injury/acute respiratory distress syndrome, EUA, emergency use authorization
CTSL: Cathepsin L
CTSB: Cathepsin B
PCR: Polymerase chain reaction
