# Supplementary material for: Beyond the vaccines: a glance at the small molecule and peptide-based anti-COVID19 arsenal
Source: J Biomed Sci. 2022 Sep 6;29:65. doi: 10.1186/s12929-022-00847-6 (PMC9444709; doi:10.1186/s12929-022-00847-6)
Supplement: Supplementary file 1 — Additional file 1: Table S1. Activity of profile of small molecules as anti-COVID drugs. [file 12929_2022_847_MOESM1_ESM.pdf]

Supporting Information

Title: Beyond the vaccines: A glance at the small molecule and peptide-based Anti-COVID19 arsenal

Kunal Nepali [a,b], Ram Sharma[a], Sachin Sharma[a], Amandeep Thakur[a], Jing-Ping Liou [a,b,*]

[a] School of Pharmacy, College of Pharmacy, Taipei Medical University, 250 Wuxing Street, Taipei 11031, Taiwan.

[b] TMU Research Center for Drug Discovery, Taipei Medical University, Taipei 11031, Taiwan

* Corresponding author: Jing Ping Liou

Phone no. 886-2-2736-1661 ext 6130.

E-mail: jpl@tmu.edu.tw.

Table 1S – Activity of profile of small molecules as anti-COVID drugs

Structure	Activity profile	Mechanism
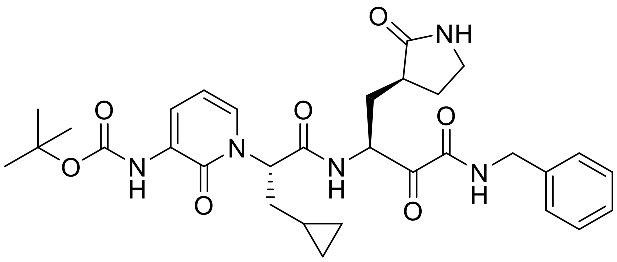 **Peptidomimetic α-ketoamide**	IC_{50} = 0.67 µM (SARS-CoV-2 Mpro)	Protease Inhibitor [50]
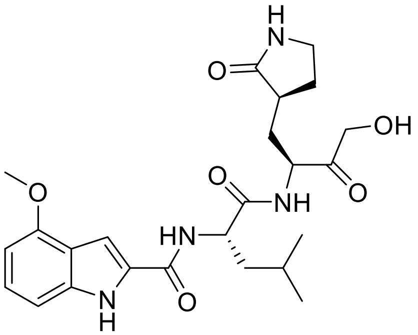 **PF-00835231**	IC_{50} (SARS-CoV2-3CL_{PRO}) = 4 nM	Protease Inhibitor [51]
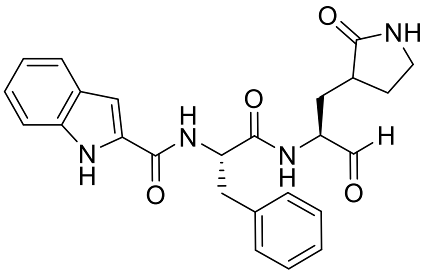 **Peptidomimetic aldehyde**	IC_{50} (SARS-CoV2-3CLPRO) = 0.34 µM<br>EC_{50} (SARS-CoV-2) = 0.29 µM	Protease Inhibitor [54]
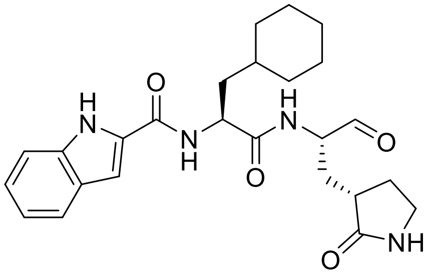 **Peptidomimetic aldehyde**	IC_{50} (SARS-CoV-2) = 0.53 µM	Protease Inhibitor [45]
	IC_{50} (SARS-CoV-2) = 0.72 µM	Protease Inhibitor [45]

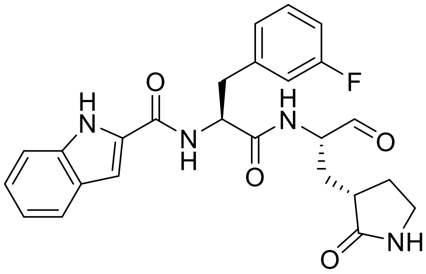<br>**Peptidomimetic aldehyde**		
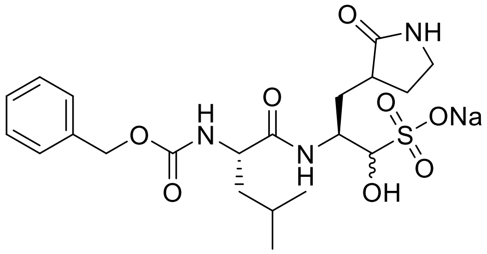<br>**GC376**	IC_{50} = 2.883 µM (CPE reduction assay)<br><br>IC_{50} (SARS-CoV-2 3CLpro) = 151 nM	Protease Inhibitor [57, 60]
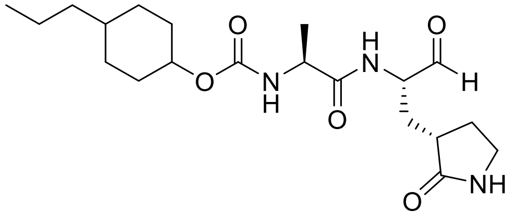<br>**Dipeptide**	IC_{50} (SARS-CoV-2 3CLpro) = 0.17 µM	Protease Inhibitor [57]
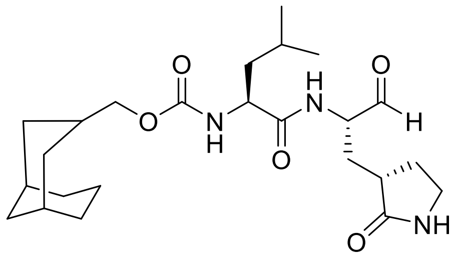<br>**Dipeptide**	EC_{50} (SARS-Cov-2) = 0.035 µM.	Protease Inhibitor [58]
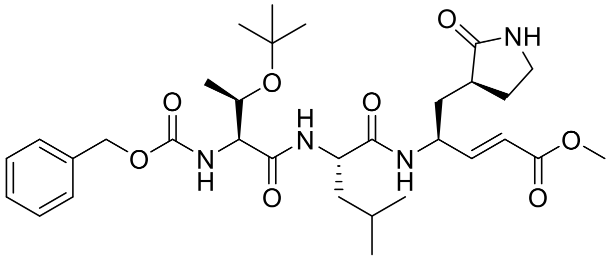<br>**Dipeptide**	IC_{50} = 2.189 µM (CPE reduction assay)<br><br>IC_{50} (SARS-CoV-2 3CLpro) = 160 nM	Protease Inhibitor [60]
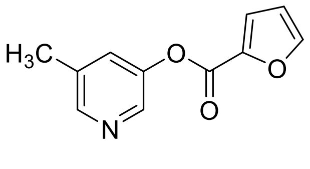<br>**MAC-5576**	IC_{50} (SARS-CoV-2 3CLpro) = 81 nM	Protease Inhibitor [60]

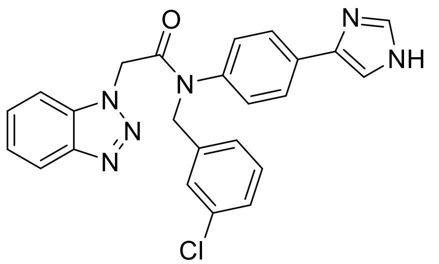<br>**Noncovalent small molecule SARS-CoV-2 inhibitor**	EC_{50} (SARS-Cov2 3CLpro) = 68 nM,<br><br>EC_{50} (anti-viral CPE inhibition) = 497 nM<br><br>EC_{50} = 558 nM (plaque reduction)	Protease Inhibitor [62]
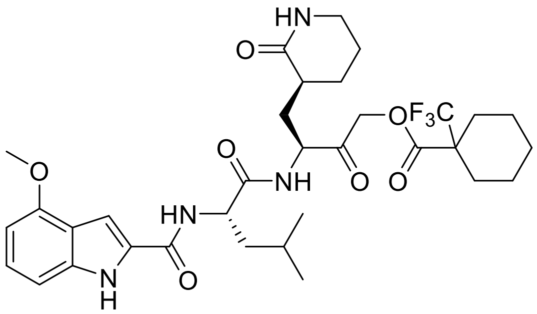<br>**α-acyloxymethylketone warhead peptidomimetic scaffold**	IC_{50} (SARS-CoV-2 3CLpro) = 86 nM	Protease Inhibitor [63]
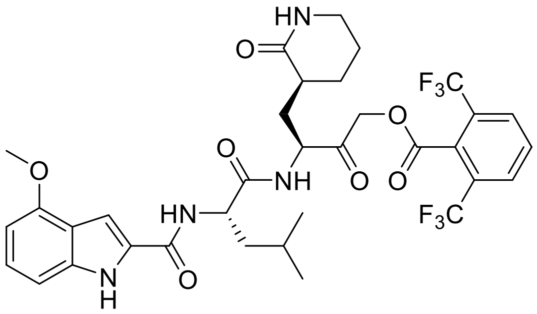<br>**α-acyloxymethylketone warhead peptidomimetic scaffold**	IC_{50} (SARS-CoV-2 3CLpro) = 1 nM	Protease Inhibitor [63]
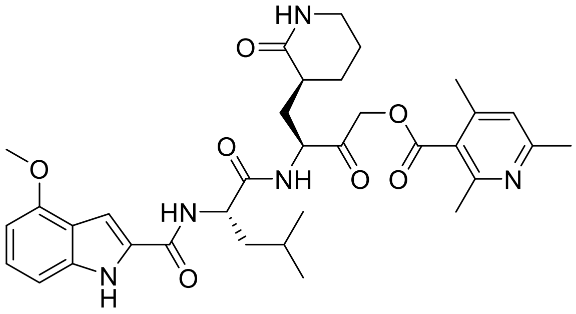<br>**α-acyloxymethylketone warhead peptidomimetic scaffold**	IC_{50} (SARS-CoV-2 3CLpro) = 19 nM	Protease Inhibitor [63]
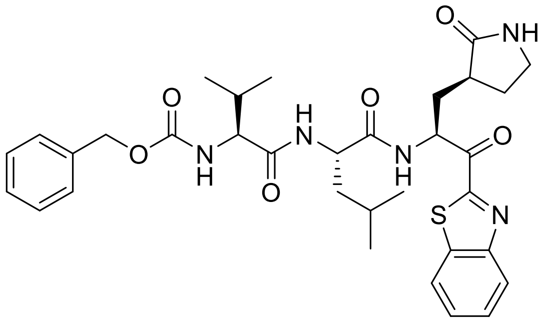<br>**SH-53**	Ki (SARS-CoV-2 3CLpro) = 14.5 nM	Protease Inhibitor [64]

**Benzothiazolyl ketone based inhibitor**		
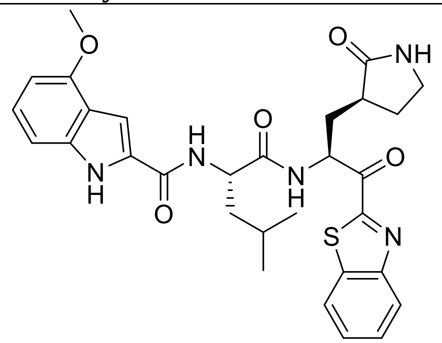<br>**YH 53**<br>**Benzothiazolyl ketone based inhibitor**	Ki (SARS-CoV-2 3CLpro) = 34.7 nM	Protease Inhibitor [64]
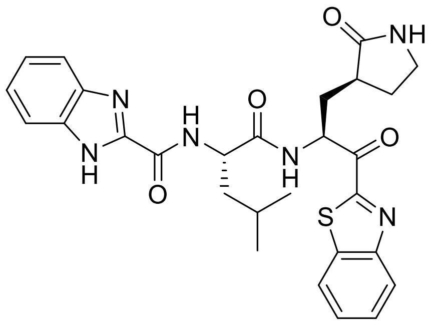<br>**YH 71**<br>**Benzothiazolyl ketone based inhibitor**	Ki (SARS-CoV-2 3CLpro) = 32.1 Nm	Protease Inhibitor [64]
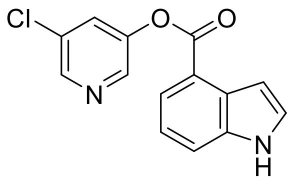<br>**GRL-0920**<br>**Indole-chloropyridinyl-ester**	EC_{50} (SARS-CoV-2) = 2.8 μM	Protease Inhibitor [66, 67]
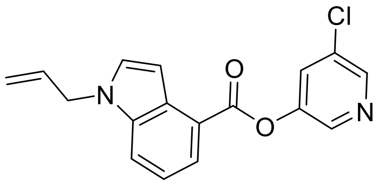<br>**Indole-chloropyridinyl-ester**	IC_{50} (SARSCoV-2 3CLpro) = 73 nM	Protease Inhibitor [67]
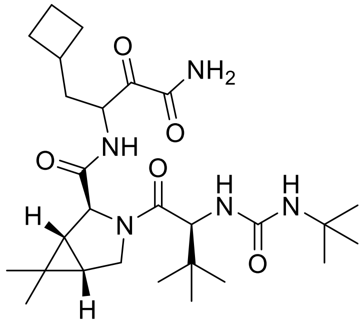<br>**Boceprevir**	EC_{50} (SARS-CoV-2 viral replication) = 0.49 - 3.37 μM	Protease Inhibitor [70]

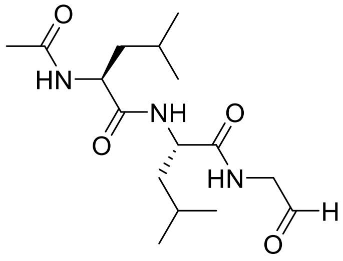 **Calpain inhibitor II**	EC_{50} (SARS-CoV-2 viral replication) = 0.49 - 3.37 μM	Protease Inhibitor [70]
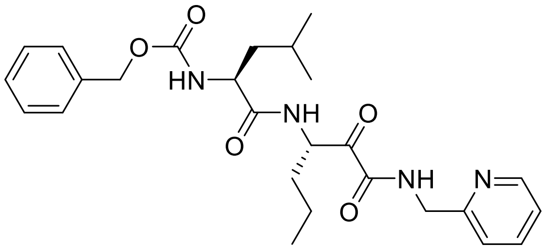 **Calpain inhibitor XII**	EC_{50} (SARS-CoV-2 viral replication) = 0.49 - 3.37 μM	Protease Inhibitor [70]
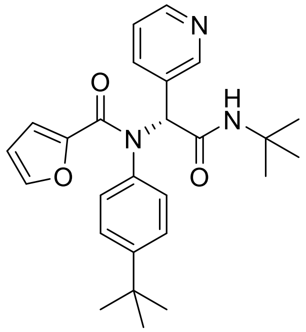 **ML188**	IC_{50} (SARS-CoV Mpro inhibitor) = 1.5 ± 0.3 μM).<br><br>EC_{50} (SARS-CoV viral replication) = 12.9 μM	Protease Inhibitor [73]
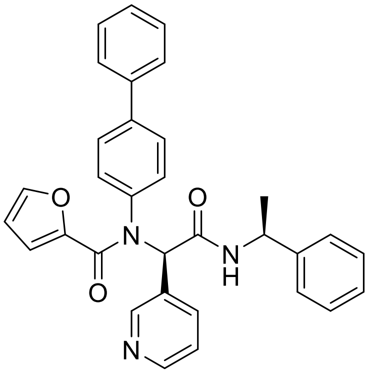 **ML188 analog**	EC_{50} (SARS-CoV Mpro) = 1.27 μM	Protease Inhibitor [73]
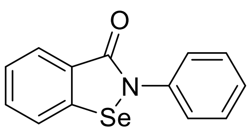 **Ebselen, organoselenium compound**	EC_{50} (SARS-CoV-2) = 4.67 μM	Protease Inhibitor [77]

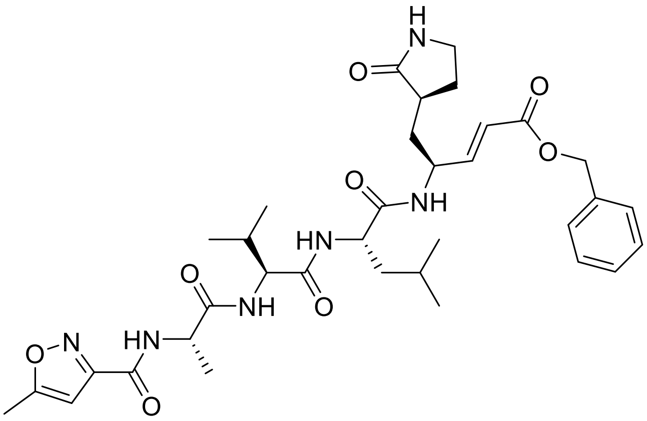 **N3, a michael acceptor inhibitor**	EC_{50} (SARS-CoV-2) = 16.77 µM	Protease Inhibitor [77]
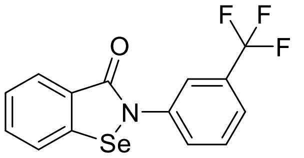 **Ebselen derivative**	EC_{50} value (SARS-CoV-2-infected Vero E6 cells) = 844 nM	Protease Inhibitor [79]
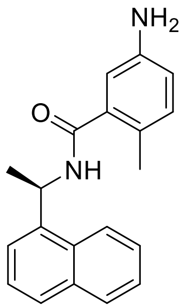 **GRL0617**	IC_{50} (SARS-CoV PLpro inhibitor) = 1.61 µM	Protease Inhibitor [80]
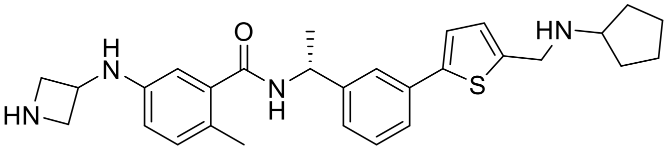 **Thiophene based inhibitor**	IC_{50} (SARS-CoV PLpro inhibitor) = 0.39 µM	Protease Inhibitor [80]
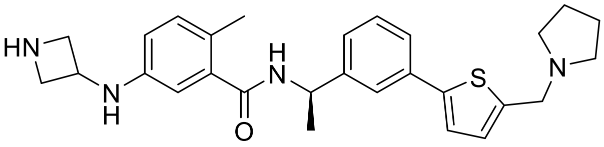 **Thiophene based inhibitor**	IC_{50} (SARS-CoV PLpro inhibitor) = 0.56 µM	Protease Inhibitor [80]
**EC-M** 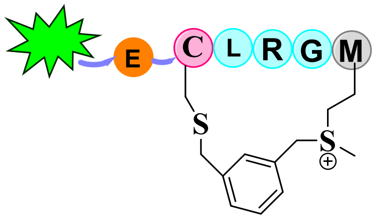	IC_{50} (SARS-CoV PLpro inhibitor) = 3.43 ± 0.54 and 16.38 ± 0.81 µM	Protease Inhibitor [81]

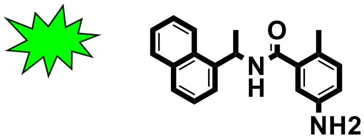 **Peptide−drug conjugate**		
EM-C 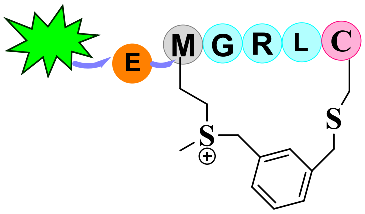 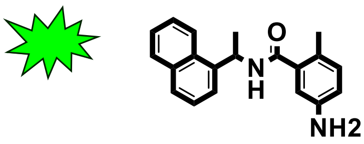 **Peptide−drug conjugates**	IC_{50} (SARS-CoV PLpro inhibitor) = 16.38 ± 0.81 μM	Protease Inhibitor [81]
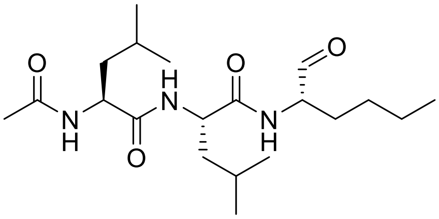 **Calpain inhibitor I**	EC_{50} (inhibition of viral infection in monkey derived vero E6 cells) = 0.28 μM	Protease Inhibitor [82]
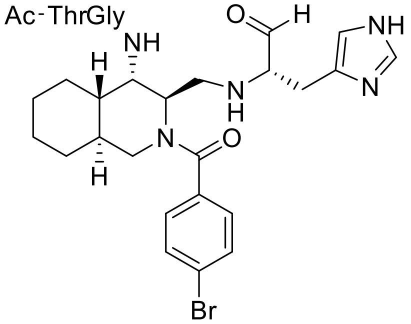 **Decahydroisoquinoline based inhibitor**	IC_{50} (R188I SARS 3Clpro)= 26 μM	Protease Inhibitor [84]
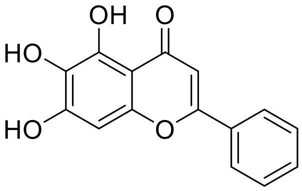 **Baicalein**	IC_{50} (SARS-CoV-2 $3CL^{pro}$ inhibitory activity) = 0.39 μM	Protease Inhibitor [88]

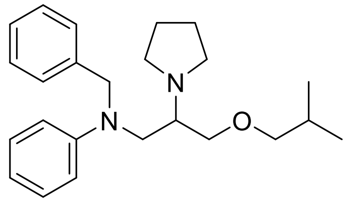 **Bepridil**	IC_{50} value (inhibition of CPE induced by SARSCoV-2 infection in Vero E6) = 0.86 µM	Protease Inhibitor [89]
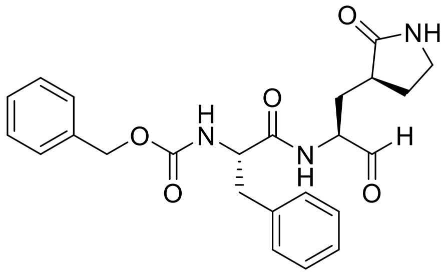 **β-(S-2-oxopyrrolidin-3-yl)-alaninal bearing adduct**	IC_{50} (SARS-CoV-2 Mpro) = 8.5 nM	Protease Inhibitor [90]
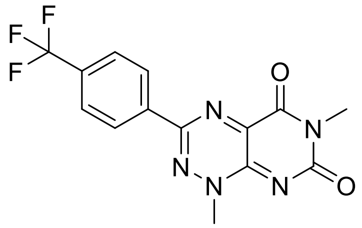 **Walrycin B**	IC_{50} (SARS-CoV-2 3CLpro) = 0.26 µM	Protease Inhibitor [92]
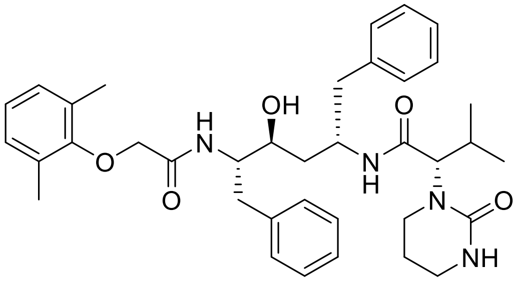 **Lopinavir**	EC_{50} (SARS-CoV-2 virus in Vero E6 cells) = 26.63 µM	Protease Inhibitor [94]
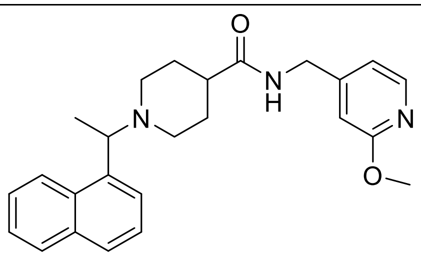 **Naphthalene based inhibitor**	IC_{50} (SARS-CoV-2 PLpro) = 0.81 µM	Protease Inhibitor [98]

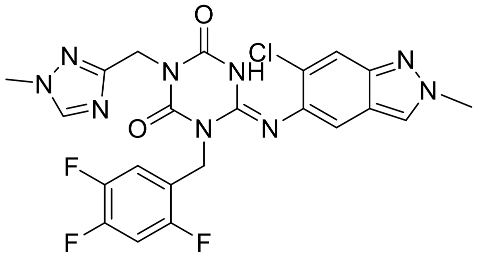 **S-217622**	IC_{50} (SARS-CoV-2 3CLpro) = 0.13 μM	Protease Inhibitor [104]
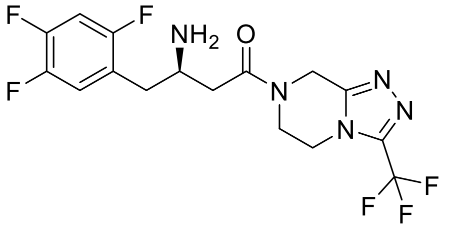 **Sitagliptin**	EC_{50} (SARS-CoV-2) = 0.32 μM	Protease Inhibitor [106]
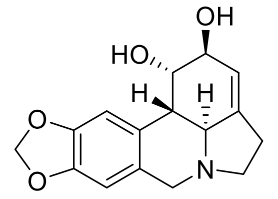 **Lycorine**	EC_{50} (M_{PRO}) = 0.01 μM	Protease Inhibitor [106]
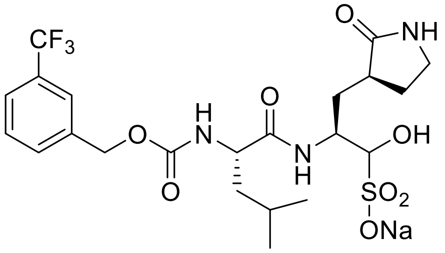 **Coronastat**	IC_{50} (SARS-CoV-2 3CL pro) = 16 nM)	Protease Inhibitor [114]
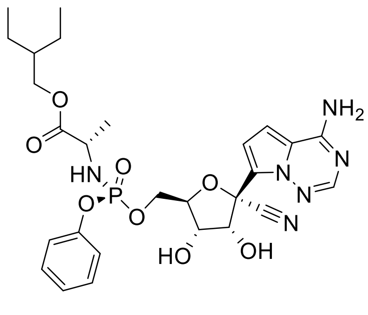 **Remdesivir**	EC_{50} (SARS-CoV-2 infection) = 0.77 μM	RdRp inhibitor [115]

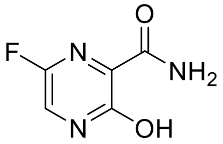 **Favipiravir**	IC_{50} (R_dRP) = 341 nM.	RdRp inhibitor [139]
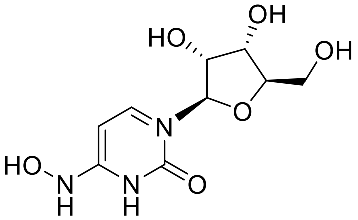 **β-D-N4-hydroxycytidine**	IC_{50} (SARS-CoV-2 in Vero cells) = 0.3 mmol/L	Replication Inhibitor [140]
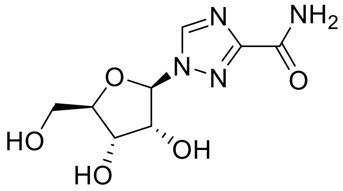 **Ribavirin**	EC_{50} (anti-SARS-CoV-2 activity) = 109.5 μM	Replication Inhibitor [126]
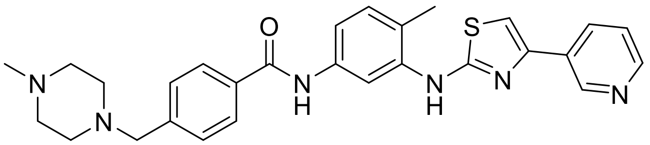 **Masitinib**	IC_{50} (SARS-CoV-2 main protease 3CLpro) = 3.2 µmol/L	Tyrosine kinase inhibitor [149]
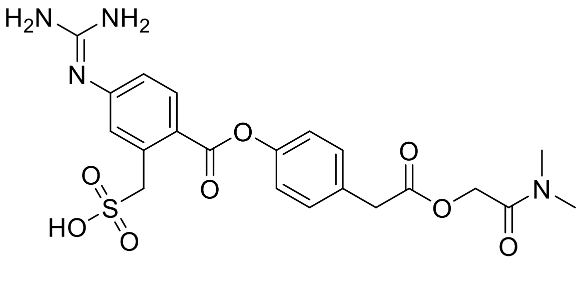 **Camostat mesylate**	IC_{50} (SARS-CoV-2 pseudovirus entry into Calu-3 cells) = 1 µmol/L).	TMPRSS2 inhibitor [162]

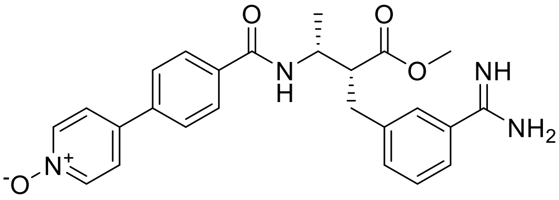 **Otamixaban**	IC_{50} (TMPRSS2 inhibitor) = 0.62 μM.	TMPRSS2 inhibitor [166]
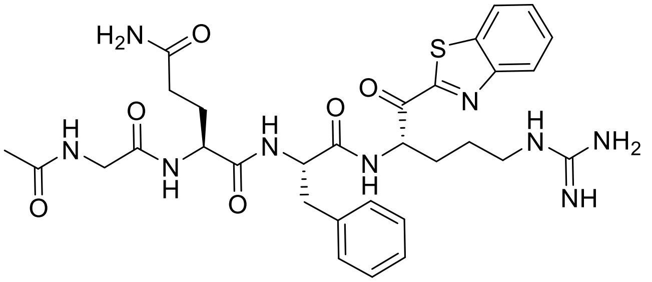 **MM3122**	IC_{50} (VSV- SARS-CoV-2 chimeric viral entry) = 340 pM	TMPRSS2 inhibitor [167]
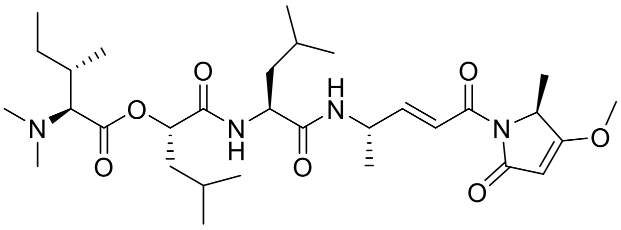 **Gallinamide A analogue**	IC_{50} = 1.76 X 10-5 mM (cat L)<br>EC_{50} = 2.8 mM X 10-2 (SARS-CoV-2)	Cathepsin L [170]
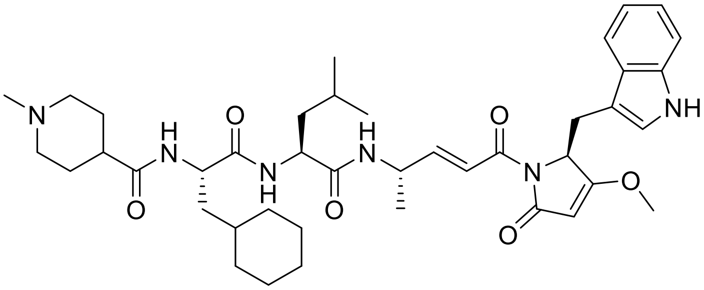 **Gallinamide A analogue**	IC_{50} = 8.57 mM X 10-6 mM (cat L)<br>EC_{50} = 6.07 mM (SARS-CoV-2)	Cathepsin L [170]
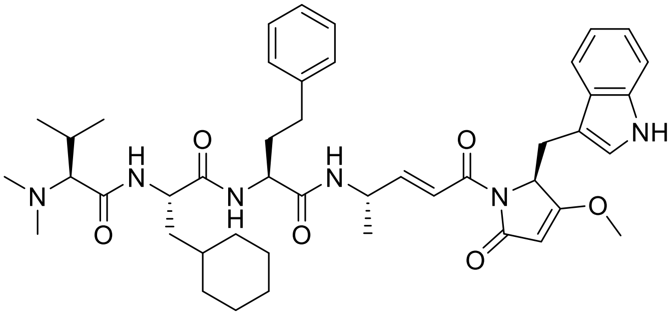 **Gallinamide A analogue**	IC50 = 5.60 mM X 10-6 mM (cat L)<br>EC50 = 1.68 mM x 10-1 (SARS-CoV-2)	Cathepsin L [170]

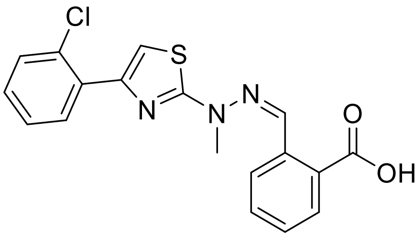 **S416**	EC_{50} (DHODH inhibition) =17 nmol/L)	DHODH Inhibitor [174]
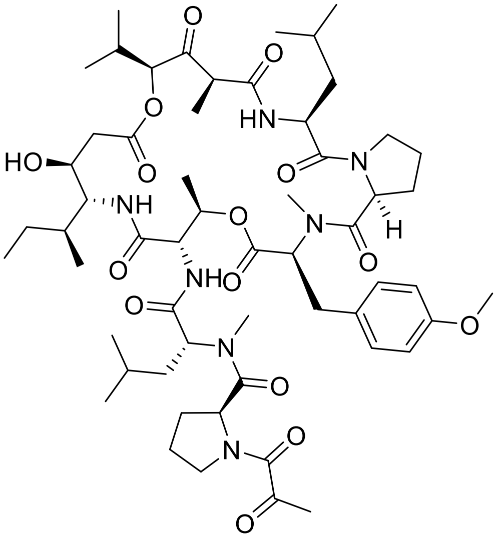 **Plitidepsin**	IC_{90} (eEF1A inhibition) = 0.88 nM	Eukaryotic translation elongation factor 1A (eEF1A) inhibitor [179]
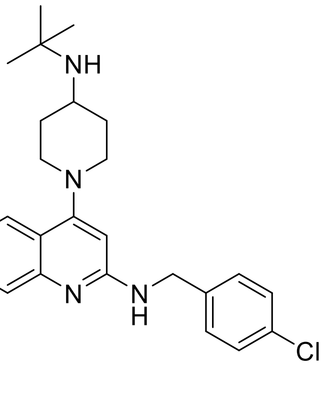 **GNS561**	EC_{50} (SARS-CoV-2 strain, IHU-MI3 -MI6) = 0.006 μM)	Autophagy inhibitor [183]
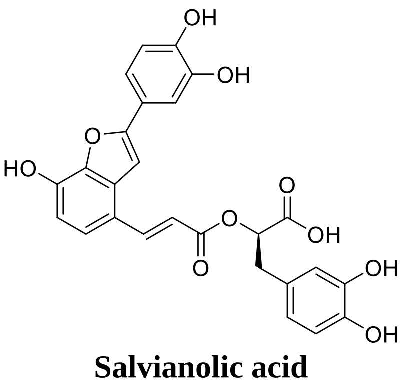 **Salvianolic acid**	EC_{50} (SARS-CoV-2) = 3.41 μM	Inhibitor of membrane fusion of S-overexpressed-HEK293T and Vero-E6 cells [185]

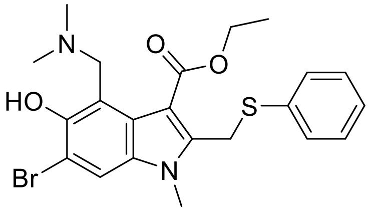<br>**Arbidol**	IC_{50} (SARS-CoV-2) = 4.11 µM	Blocks trimerization of SARS-CoV-2 spike glycoprotein [186]
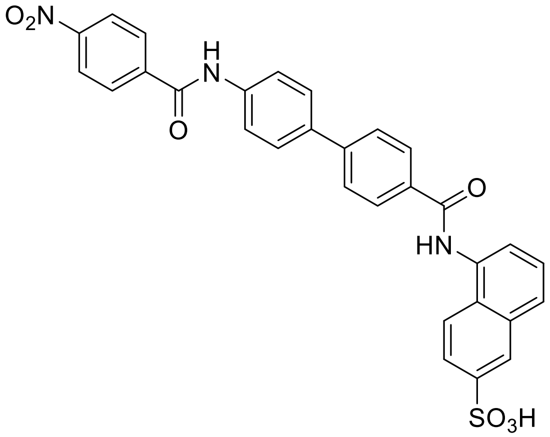<br>**Biaryl scaffold**	IC_{50} (SARS-CoV-2-S pseudovirus inhibitory activity) = 5.6 mmol/L	Inhibitor of the hACE2 – S protein interaction of SARS-CoV-2 [189]
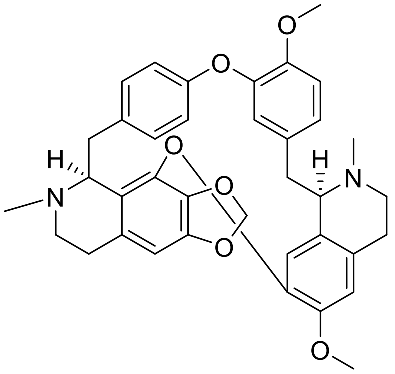<br>**Small molecule spike mediated entry inhibitor**	EC_{50} (CPE reduction) = 1.41 µM	Broad spectrum spike mediated entry inhibitor [190]
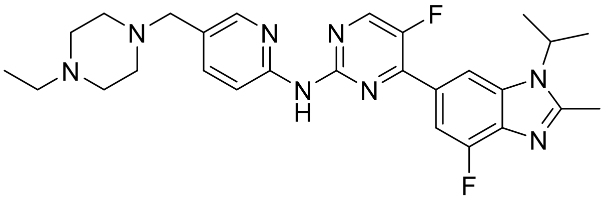<br>**Small molecule spike mediated entry inhibitor**	EC_{50} (CPE reduction) = 3.16 µM	Broad spectrum spike mediated entry inhibitor [190]

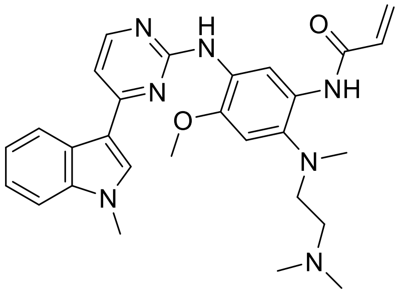<br>**Small molecule spike mediated entry inhibitor**	EC_{50} (CPE reduction) = 3.98 µM	Broad spectrum spike mediated entry inhibitor [190]
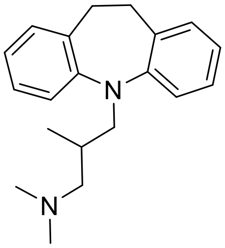<br>**Small molecule spike mediated entry inhibitor**	EC_{50} (CPE reduction) = 20.52 µM	Broad spectrum spike mediated entry inhibitor [190]
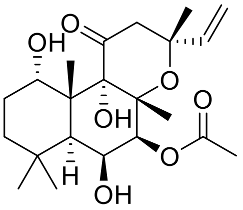<br>**Small molecule spike mediated entry inhibitor**	EC_{50} (CPE reduction) = 23.06 µM	Broad spectrum spike mediated entry inhibitor [190]
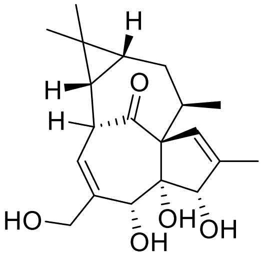<br>**Small molecule spike mediated entry inhibitor**	EC_{50} (CPE reduction) = 0.06 µM	Broad spectrum spike mediated entry inhibitor [190]
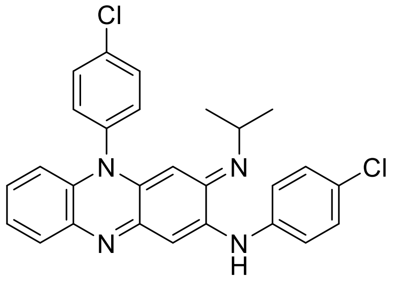<br>**Clofazimine**	EC_{50} (SARS-CoV-2) = 0.31 µM	SARS-CoV-2 entry prevention [191]

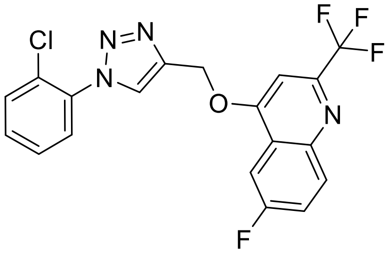 **Triazole based inhibitor**	IC_{50} (SARS-CoV-2) = 0.060 mm	Antiviral drug
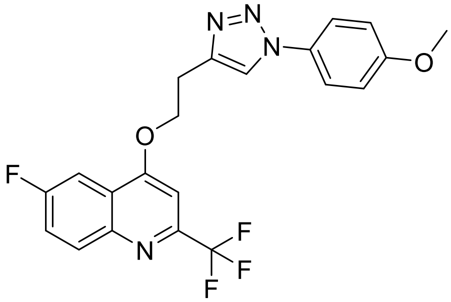 **Triazole based inhibitor**	IC_{50} (SARS-CoV-2) = 0.204 mm	Antiviral drug
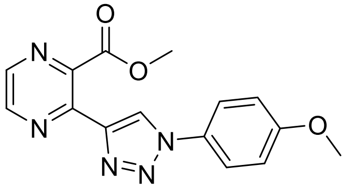 **Triazole based inhibitor**	IC_{50} (SARS-CoV-2)= 0.120 mM	Antiviral drug
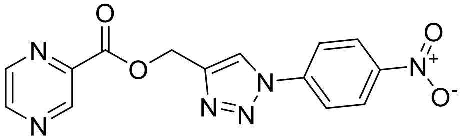 **Triazole based inhibitor**	IC_{50} (SARS-CoV-2) = 0.477 mM	Antiviral drug
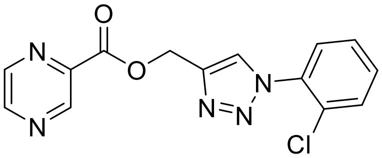 **Triazole based inhibitor**	IC_{50} (SARS-CoV-2) = 0.952 mM	Antiviral drug

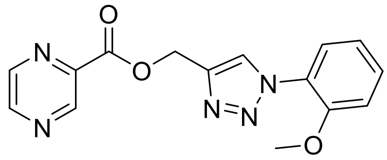 **Triazole based inhibitor**	IC_{50} (SARS-CoV-2) = 1.079 mM	Antiviral drug
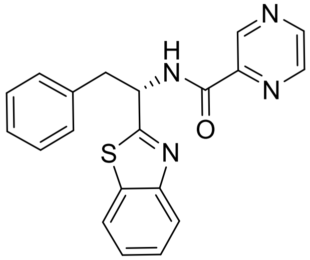 **Triazole based inhibitor**	IC_{50} (SARS-CoV-2) = 0.363 mM	Antiviral drug
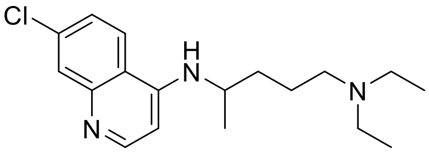 **Chloroquine**	EC_{50} (SARS-Cov-2) = 1.13 μM	Antimalarial [197]
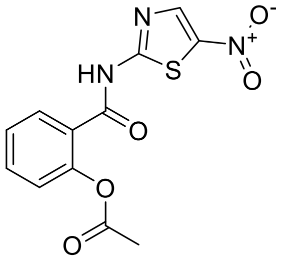 **Nitazoxanide**	EC_{50} (SARS-CoV-2) = 22.50 μM	Anti-parasitic [197]
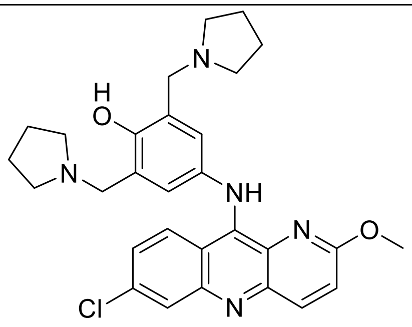 **Pyronaridine**	IC_{50} (replication of virus in A549-ACE2 cells) = 198 nM	Antimalarial [203]

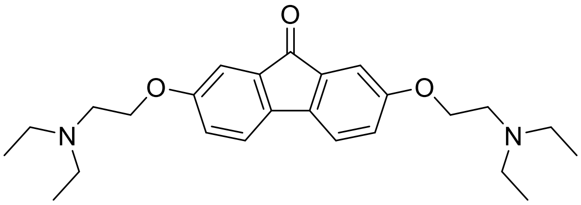 **Tilorone**	IC_{50} (replication of virus in A549-ACE2 cells) = 180 nM	Anti-SARS-CoV-2 [203]
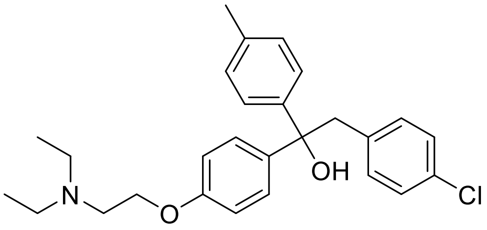 **Triparanol**	IC_{50} (SARS-CoV-2) < 10 µM	Repurposed drug [205]
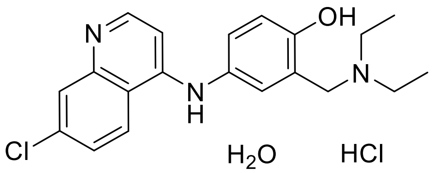 **Amodiaquine dihydrochloride dihydrate**	IC_{50} (SARS-CoV-2) < 10 µM	Repurposed drug [205]
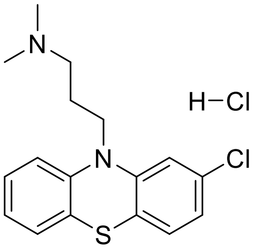 **Chlorpromazine**	IC_{50} (SARS-CoV-2) < 10 µM	Repurposed drug [205]
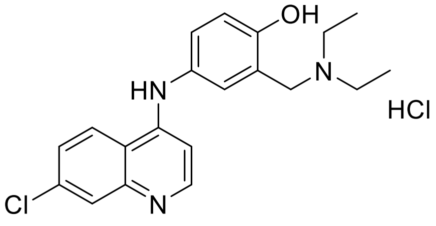 **Amodiaquine hydrochloride**	IC_{50} (SARS-CoV-2) < 10 µM	Repurposed drug [205]

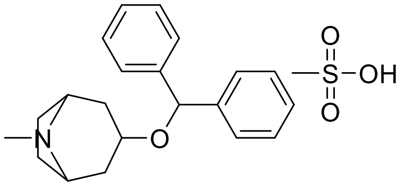<br>**Benztropine mesylate**	IC_{50} (SARS-CoV-2) < 10 µM	Repurposed drug [205]
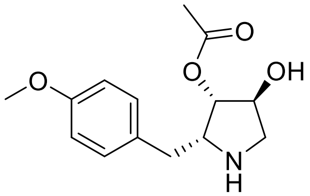<br>**Anisomycin**	IC_{50} (SARS-CoV-2) < 10 µM	Repurposed drug [205]
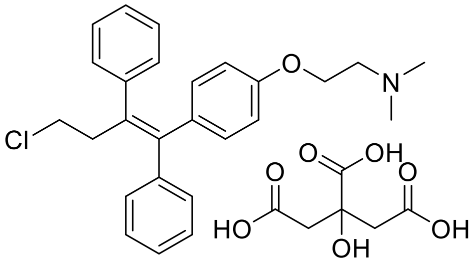<br>**Toremifene**	IC_{50} (SARS-CoV-2) < 10 µM	Repurposed drug [205]
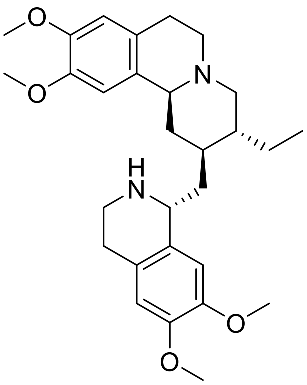<br>**Emetine dihydrochloride hydrate**	IC_{50} (SARS-CoV-2) < 10 µM	Repurposed drug [205]

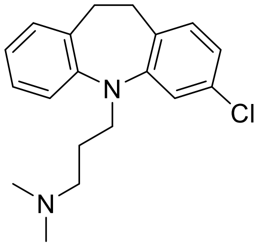 **Clomipramine**	IC_{50} (SARS-CoV-2) < 10 µM	Repurposed drug [205]
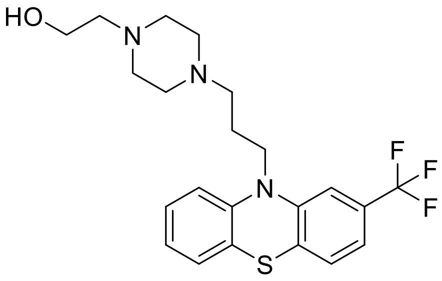 **Fluphenazine**	IC_{50} (SARS-CoV-2) < 10 µM	Repurposed drug [205]
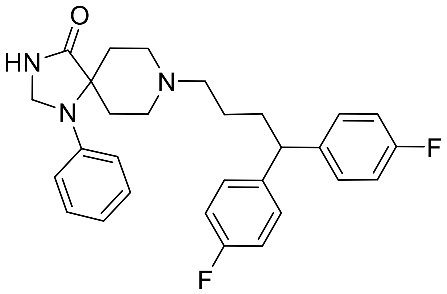 **Fluspirilene**	IC_{50} (SARS-CoV-2) < 10 µM	Repurposed drug [205]
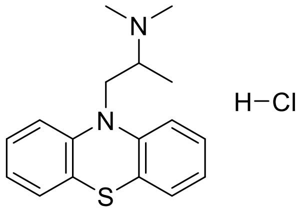 **Promethazine hydrochloride**	IC_{50} (SARS-CoV-2) < 10 µM	Repurposed drug [205]
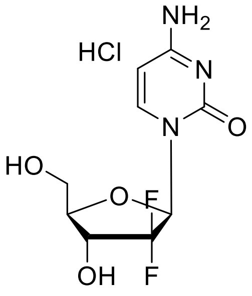	IC_{50} (SARS-CoV-2) < 10 µM	Repurposed drug [205]

**Gemcitabine hydrochloride**		
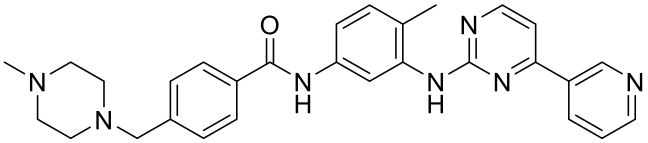<br>**Imatinib**	IC_{50} (SARS-CoV-2) < 10 µM	Repurposed drug<br>[205]
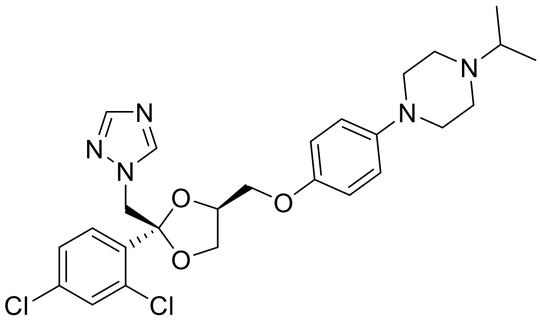<br>**Terconazole**	IC_{50} (SARS-CoV-2) < 10 µM	Repurposed drug<br>[205]
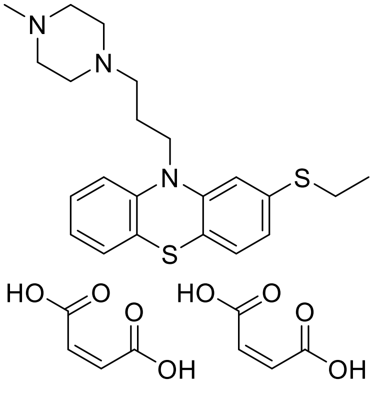<br>**Thiethylperazine maleate**	IC_{50} (SARS-CoV-2) < 10 µM	Repurposed drug<br>[205]

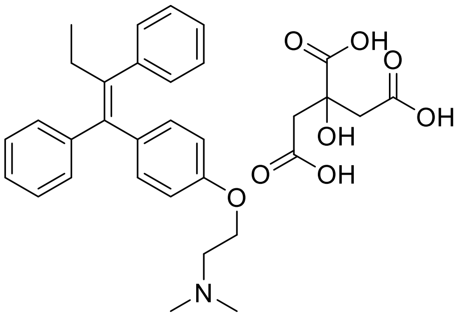<br>**Tamoxifen citrate**	IC_{50} (SARS-CoV-2) < 10 μM	Repurposed drug [205]
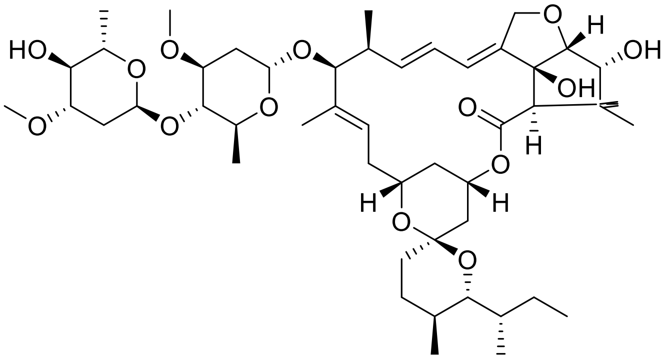<br>**Ivermectin**	IC_{50} (inhibition of the antiviral responses) = 2 μM	Anti-parasitic drug [208]
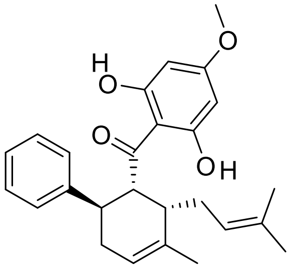<br>**Panduratin A**	IC_{50} (viral infection) = 0.81 μM	Inhibitor of SARS-CoV-2 [212]
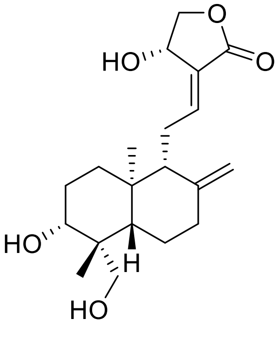<br>**Andrographolide**	IC_{50} (viral infection) = 6.58 μM	Phytoconstituent from *Andrographis paniculata* [212]
